# Shed teeth from Portezuelo formation at Sierra del Portezuelo reveal a higher diversity of predator theropods during Turonian-Coniacian times in northern Patagonia

**DOI:** 10.1186/s12862-024-02249-8

**Published:** 2024-05-10

**Authors:** Jorge Gustavo Meso, Federico Gianechini, Kevin Leonel Gomez, Luciana Muci, Mattia Antonio Baiano, Diego Pol, Jonatan Kaluza, Alberto Garrido, Michael Pittman

**Affiliations:** 1 Consejo Nacional de Investigaciones Científicas y Técnicas (CONICET), Buenos Aires, Argentina; 2 Instituto de Investigación en Paleobiología y Geología (IIPG), Universidad Nacional de Río Negro (UNRN) - Consejo Nacional de Investigaciones Científicas y Técnicas (CONICET), General Roca, Río Negro 8332 Argentina; 3 Universidad Nacional de Río Negro, Sede Alto Valle/Valle Medio, R8332 General Roca, Estados Unidos 750, Río Negro, Argentina; 4 Instituto Multidisciplinario de Investigaciones Biológicas de San Luis (IMIBIO-SL), Universidad Nacional de San Luis, San Luis, Argentina; 5 School of Life Sciences, The Chinese University of Hong Kong, Shatin, Hong Kong SAR, China; 6 DrNatali S/N, Área Laboratorio E Investigación, Museo Municipal ‘Ernesto Bachmann’, 8311 Villa El Chocon, Neuquén, Argentina; 7 Museo Paleontológico Egidio Feruglio, Trelew, Chubut Argentina; 8 Fundación de Historia Natural Félix de Azara, Universidad Maimónides. Hidalgo 775, Ciudad Autónoma de Buenos Aires, C1405 Argentina; 9 Museo Provincial de Ciencias Naturales ‘Prof. Dr. Juan A. Olsacher’. Dirección Provincial de Minería, Zapala, Neuquén, Argentina

**Keywords:** Portezuelo formation, Middle Turonian-late Coniacian, Teeth theropods, Megaraptoridae, Abelisauridae, Alvarezsauridae

## Abstract

The study of thirty-two shed crowns from the Portezuelo Formation (middle Turonian-late Coniacian) at the Sierra del Portezuelo locality, reveals six distinct tooth morphotypes identified through cladistic, discriminant, and cluster analyses. Two morphotypes were identified as belonging to Megaraptoridae, three to Abelisauridae, one to Abelisauroidea, and one to Alvarezsauridae. Additionally, two of the morphotypes exhibit a combination of dental features typically found in megaraptorid and abelisauridtheropods. These results suggest a greater diversity of theropods in the original ecosystem than previously thought, including the presence of a second morphotype of megaraptorid and alvarezsaurid previously undocumented in this formation. Furthermore, the existence of Morphotype 6 indicates the potential coexistence of medium-sized abelisauroids alongside larger abelisaurids in the same ecosystem. These findings underscore the importance of future expeditions to the Sierra del Portezuelo locality to further our understanding of these previously unknown theropod species.

**Supplementary Information:**

The online version contains supplementary material available at 10.1186/s12862-024-02249-8.

## Introduction

The middle Turonian-late Coniacian Portezuelo Formation is a continental unit of the Río Neuquén Subgroup, Neuquén Group located in the Neuquén Basin, a broad region across northern Patagonia [1]. This geological unit has provided a wealth of fossil remains, featuring a diverse fauna. Among the findings of theropod dinosaurs are the eponymous megaraptorid *Megaraptor namunhuaiquii* [2], the alvarezsaurid *Patagonykus puertai* [3, 4], the unenlagiine dromaeosaurids *Unenlagia comahuensis* [5], *Unenlagia paynemili* [6], *Neuquenraptor argentinus* [7], and *Pamparaptor micros* [8], an unnamed early-diverging basal abelisauroid [9], the abelisaurid *Elemgasem nubilus* [10], and neornithine birds [11]. As for the sauropod dinosaurs, two taxa have been formally described, the titanosaur *Futalongkosaurus dukei* [12], and *Muyelensaurus pecheni* [13]. In the Mendoza province, [14] recorded *Malarguesaurus florenciae* in the Portezuelo Formation. Regarding the ornithischian record, recovered remains belong to the early-diverging iguanodont *Macrogryphosaurus gondwanicus* [15].

Through two recent paleontological field expeditions to outcrops of the Portezuelo Formation at the Sierra del Portezuelo locality (Neuquén Province, Argentina), thirty-two shed teeth identified as belonging to theropods, were discovered. This study aims to offer a comprehensive description of this theropod dental material, employing the latest phylogenetic and morphometric techniques for identification, and discussing the paleoecological implications of the discoveries to what we know about middle Turonian-late Coniacian theropods.

### Geological and geographical setting

**Fig. 1:**
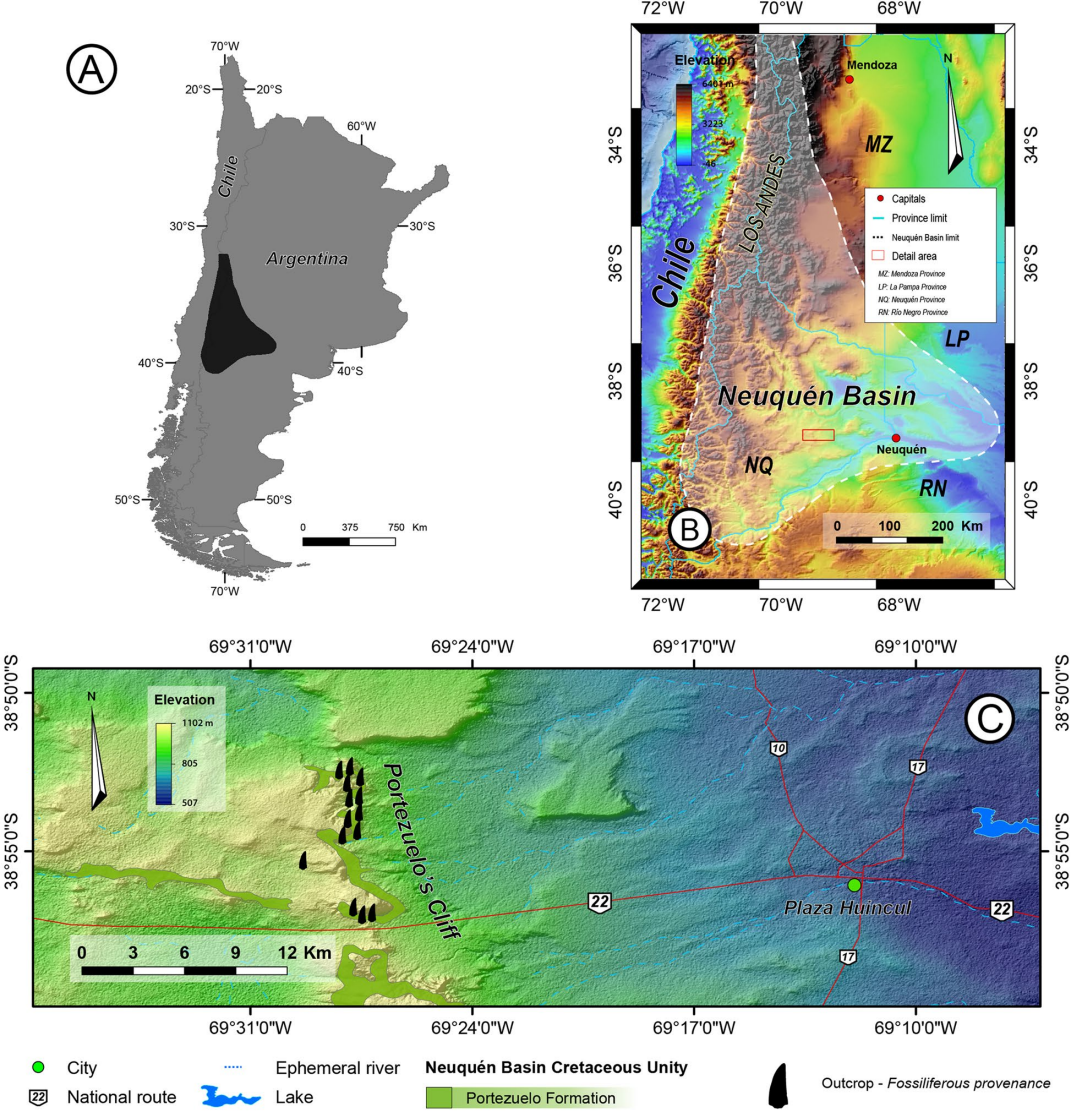
Location maps of the study area within the Neuquén Basin (**A, B**). Geological map indicating the Portezuelo Formation recognized in Sierra del Portezuelo (**C**)

The thirty-two shed teeth described here come from the Portezuelo Formation, which outcrops extended along the Sierra del Portezuelo (lithostratigraphic locality type), a small range situated 20 km to west of the Cutral Có city, Neuquén Province, Argentina (Fig. 1). The specimens examined were recovered from two successive fieldtrips to Sierra del Portezuelo during February and November of 2023.

The Portezuelo Formation is currently assigned to the middle Turonian to late Coniacian (Late Cretaceous) [1], although so far there is no absolute dating that certifies an precise age for this succesion. Lithologically, this unit consists of an approximately 100 m thick succession of yellowish sandy deposits, interbedded with variable thicknesses of red and green mudstones beds. Sandstone beds mainly correspond to fill fluvial channel deposits, as well as crevasse channel fills and/or crevasse splay deposits. The mudstone beds constitute the floodplain fines deposits, frequently associated with paleosoils development. Paleoenvironmentally, this succession is attributed to the development of a sand-bed meandering fluvial system [1].

The teeth collected in deposits of this unit, appear under two taphonomic modes. The first mode corresponds to the occurrence of isolated teeth, associated to fluvial bar deposits. In these cases, the teeth are usually associated to cross-stratified sandstone facies (Sp facies sensu [16]), and arranged in the lower sector of the lee faces (toesets) developed as large-scale bedforms or macroforms that constitutes the river bar system. The second taphonomic mode happens in the form of small teeth concentrations, and constituted the more frequent occurrence style. In these cases, the teeth are associated to small lenticular sandy bodies, granulometrically poorly selected, also being frequent the presence of pelitic intraclasts and bioclasts composed of small, highly eroded bone fragments. These deposits resemble the Se and Ss facies of [16, 17], and are here interpreted as channel lag deposits. In the two mode of occurrences, the teeth present varying degrees of abrasion; which is consistent with its allochthonous origin and its tractive mode of transport developed under the action of the river currents.

## Materials and methods

### Comparative methodology and terminology

The materials examined in this paper accessioned as MCF-PVPH-920 to 951 are housed at the Museo “Carmen Funes” in Plaza Huincul, Neuquén, Argentina. The specimens were examined first-hand using a Nikon SMZ/800 binocular microscope under different magnifications at the Museo “Carmen Funes”. Fourteen measurement variables (i.e., CBL, CBW, CH, AL, CBR, CHR, MC, DC, MCL, MCW, MCR, MDL, DDL, DSDI; Table 1) were taken on the best-preserved tooth crowns with digital calipers with an accuracy of 0.01 mm. We followed the dental nomenclature and protocol proposed by [18] for a comprehensive description of the teeth. The dental material was compared with the teeth of 156 non-avian theropod species, with a specific focus on the Argentine taxa [19]. The orientation of theropod teeth adhered to the positional terminology established by [18, 20]. Additionally, the description and labeling of each dental morphotype was based on the dental terminology proposed by [18, 21]. The phylogenetic definitions of theropod clades were determined following the definitions provided by [19].

**Table 1:** Measurements (in mm) of the teeth studied. Number of denticles per five millimeters

Field trip	Specimen	GPS point	Morphotype	Anatomical placement	Source	CBL	CBW	CH	AL	CBR (CBW/CBL)	CHR (CH/CBL)	MC	DC	MCL	MCW	MCR (MCW/MCL)	MDL (5/MC)	DDL (5/DC)	DSDI (MC/DC)
**February**	MCF PV-PH 920	S 38°56′49.5'' W 69°28′00.8''	Morphotype 1	Lateral teeth	This study	16.22	12.92	35.9	38.9	0.79	2.21	-	15	48.54	41.9	0.86	-	0.33	-
	MCF PV-PH 921	S 38°56′49.5'' W 69°28′00.8''	Morphotype 2	Lateral teeth	This study	14.65	8.93	?	?	0.6	?	-	13	12.78	7.25	0.56	-	0.38	-
	MCF PV-PH 922					9.9	5.52	?	?	0.55	?	-	13	8.95	5.65	0.63	-	0.38	-
	MCF PV-PH 923	S 38°52′36.7'' W 69°28′38.3''	Morphotype 3	Lateral tooth	This study	14.6	6.38	24.82	25.34	0.43	1.7	12	12	11.72	4.9	0.41	0.41	0.41	1
	MCF PV-PH 924	S 38°55′00.20'' W 69°28′10.03''	Morphotype 2	Lateral tooth	This study	?	?	?	?	?	?	-	17	9.4	5.92	0.62	-	0.29	-
	MCF PV-PH 925			Anatomical placement indet	This study	?	?	?	?	?	?	?	12	?	?	?	?	0.41	?
	MCF PV-PH 926	S 38°54′59.50'' W 69°28′21.3''	Morphotype 4	Lateral tooth	This study	2.48	?	?	?	?	?	-	-	1.9	?	?	-	-	-
**November**	MCF PV-PH 927	S 38°54′40.5'' W 69°28′23.3''	Morphotype 3	Lateral teeth	This study	?	6.45	?	?	?	?	12.5	12.5	12.18	5.5	0.45	0.4	0.4	1
	MCF PV-PH 928					?	?	?	?	?	?	?	?	?	?	?	?	?	?
	MCF PV-PH 929	S 38°54′40.5'' W 69°28′23.3''	Morphotype 2	Lateral teeth	This study	7.62	4.38	?	?	0.57	?	-	?	6.25	3.32	0.53	-	?	-
	MCF PV-PH 930					?	?	?	?	?	?	-	12.5	?	?	?	?	0.4	-
	MCF PV-PH 931	S 38°54′31.3'' W 69°27′59.7''	Morphotype 5	Lateral teeth	This study	9.75	4.33	?	?	0.44	?	20	15	8.64	4.04	0.46	0.25	0.33	1.33
	MCF PV-PH 932					6.38	3.33	?	?	0.52	?	25	22.5	6.15	2.79	0.45	0.2	0.22	1.11
	MCF PV-PH 933	S 38°54′31.3'' W 69°27′59.7''	Morphotype 2	Lateral teeth	This study	12.27	6.32	20.12	23.8	0.51	1.63	-	15	9.9	5.62	0.56	-	0.33	-
	MCF PV-PH 934					12	7.27	?	?	0.6	?	-	15	9.82	6.15	0.62	-	0.33	-
	MCF PV-PH 935	S 38°56′06.0'' W 69°29′03.1''	Morphotype 2	Lateral tooth	This study	11.3	6	?	?	0.53	?	-	15	9.89	5.24	0.52	-	0.33	-
	MCF PV-PH 936	S 38°54′31.5'' W 69°27′59.7''	Morphotype 3	Mesial tooth	This study	?	?	?	?	?	?	12	12	10.78	5.7	0.52	0.41	0.41	1
	MCF PV-PH 937	S 38°55′2.4'' W 69°28′17.8''	Morphotype 2	Lateral tooth	This study	11.38	6	?	?	0.52	?	-	18	9.36	5.06	0.54	-	0.27	-
	MCF PV-PH 938	S 38°54′25.6'' W 69°28′01.41''	Morphotype 2	Lateral teeth	This study	10.64	?	?	?	?	?	-	13	9.12	?	?	-	0.38	-
	MCF PV-PH 939					9.33	?	?	?	?	?	-	?	8.25	?	?	-	?	-
	MCF PV-PH 940					7.48	3.95	12.6	14.5	0.52	1.68	-	15	6.17	3.47	0.56	-	0.33	-
	MCF PV-PH 941					?	?	?	?	?	?	-	12	8.4	4.32	0.51	-	0.41	-
	MCF PV-PH 942	S 38°55′03.5'' W 69°28′12.2''	Morphotype 6	Lateral tooth	This study	?	?	?	?	?	?	12	12	13.26	6.1	0.46	0.41	0.41	1
	MCF PV-PH 943	S 38°56′53.5'' W 69°27′57.2''	Morphotype 1	Lateral tooth	This study	12.68	6.82	18.78	23.6	0.53	1.48	-	13	10.32	5.4	0.52	-	0.38	-
	MCF PV-PH 944	S 38°55′06.6'' W 69°28′00.6''	Morphotype 2	Lateral tooth and Mesial tooth?	This study	12.34	7.14	?	?	0.57	?	-	13	10.9	6.74	0.61	-	0.38	-
	MCF PV-PH 945					?	?	?	?	?	?	-	?	?	?	?	?	?	-
	MCF PV-PH 946	S 38°54′21.5'' W 69°27′51.7''	Morphotype 3	Lateral tooth	This study	?	?	?	?	?	?	?	12	10.24	5.42	0.52	?	0.41	?
	MCF PV-PH 947	S 38°56′45.8'' W 69°28′20.9''	Morphotype 2	Lateral teeth	This study	?	?	?	?	?	?	-	15	12.05	7.35	0.61	-	0.33	-
	MCF PV-PH 948				This study	?	?	?	?	?	?	?	?	?	?	?	?	?	-
	MCF PV-PH 949				This study	?	?	?	?	?	?	?	?	?	?	?	?	?	-
	MCF PV-PH 950	S 38°56′45.8'' W 69°28′20.9''	Morphotype 3	Lateral teeth	This study	?	?	?	?	?	?	12	15	8.87	4.52	0.5	0.41	0.33	0.8
	MCF PV-PH 951				This study	?	?	?	?	?	?	?	13	11.4	6.35	0.55	?	0.38	?

*Abbreviations* *AL* Apical length, *CBL* Crown base longitudinal, *CBR* Crown base ratio, *CBW* Crown base width, *CH* Crown height, *CHR* Crown height ratio, *DC* Disto-central denticle density, *DSDI* Denticle size density index, *MC* Mesio-central denticle density, *MCL* Mid crown length, *MCR* Mid-crown ratio, *MCW* Mid-crown width

### Cladistic analysis

The phylogenetic affinities of the dental material were explored after the inclusion of all teeth in the dentition-based data matrix created by [22] that was expanded by [19]. Our data matrix includes 146 scored characters across 106 genus-level operational taxonomic units (OTUs) (see Supplementary Information 1). Rather than individually scoring each tooth, we identified six distinct dental morphotypes within the theropod tooth sample (Table 1) that were treated as separate OTUs.

To carry out the phylogenetic analyses, we followed the methodology detailed by [23, 24] in the software *TNT v1.5* [25], using a backbone tree topology and the positive constraint command, setting the six dental morphotypes as floating terminals. The topological tree was built based on the results of the phylogenetic analyses of [26] for non-neotheropodsaurischians, [27] for non-averostranneotheropods, [28, 29] for Ceratosauria, [30- 32] for non-coelurosauriantetanurans, [33] for Tyrannosauroidea, [34] for Megaraptora, [35] for neocoelurosaurs, and [36] for Alvarezsauria. The search strategy of analysis used a combination of the tree-search algorithms Wagner trees, TBR branch swapping, sectorial searches, Ratchet (perturbation phase stopped after 20 substitutions), and Tree Fusing (5 rounds), until 100 hits of the same minimum tree length were reached (n.b., the TNT command used is “xmult = hits 100 rss fuse 5 ratchet 20”). Recovered trees were subjected to a final round of TBR branch swapping (TNT command used was “bb”). We also performed two additional cladistic analyses, one using the dentition-based dataset without constraints, and the second using a data matrix restricted to crown-based characters [19].

### Discriminant analysis

To classify and predict the optimal classifications of the thirty-two shed theropod teeth inside "family-level" groupings based on quantitative data, we performed a Discriminant Function Analysis (DFA) using the data set compiled by [37] and recently modified by [19]. This great dataset includes eleven measurements (i.e., CBL, CBW, CH, AL, MCL, MCW, MCR, MSL, LAF, LIF, MDL, DDL), taken in 1374 teeth belonging to 91 taxa (86 species and five indeterminate family-based taxa) separated into 20 monophyletic or paraphyletic groups [19, 38]. As in previous studies (*e.g.*, [19, 39]), a second analysis was performed on a dataset restricted to first-hand measurements by [24], since most researchers measure theropod crowns differently (see [24], for further information on this tendency). Considering that various teeth belong to relatively large-sized animals, a third analysis was carried out on a dataset restricted to theropod taxa with crowns of more than 20 mm. These two datasets include 764 and 439 teeth belonging to 55 and 48 theropod taxa respectively, each separated into 14 groups (Supplementary information 2). As an example, an additional analysis was carried out which included all the Argentine taxa from the database of [19]. The discriminant function analysis (DFA) was performed following the protocol of [23], in which all variables were log-transformed to normalize quantitative variables. Finally, the DFA was run in *PAST v3.19* [40] using the discriminant analysis function, and treating each tooth as unknown taxa.

### Cluster analysis

A third approach for the quantitative analysis of the shed teeth and their classification at the family and genus levels was implemented: cluster analysis using *PAST v3.19* [40]. This analysis was based solely on the dataset derived from first-hand measurements made by [24] that was restricted to taxa with teeth larger than two centimeters. The paired group algorithm and a neighbour-joining clustering technique were employed, with Euclidean distances selected as the similarity index. This approach has proven promising as a complementary analysis for the identification of isolated theropod teeth (e.g., [19, 24, 38, 39, 41]), allowing for the visualization and identification of potential taxonomic identifications based on shared quantitative characteristics.

## Results

### Systematic analysis

Theropoda Marsh, 1881.

Tetanurae Gauthier, 1986.

Megaraptora Benson, Carrano, and Brusatte, 2010.

Megaraptoridae Novas, Agnolín, Ezcurra, Porfiri, and Canale, 2013.

Gen. and sp. indet.

### Material

**Fig. 2:**
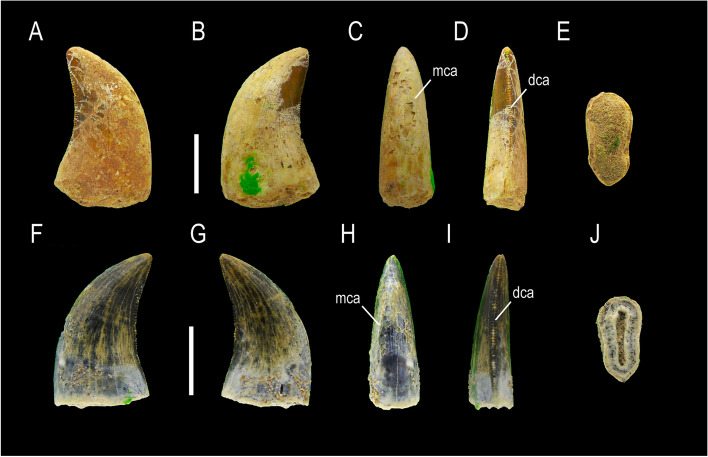
Megaraptorid teeth of Morphotype 1. MCF-PVPH-920 and MCF-PVPH-943 in labial (**A**), lingual (**B**), mesial (**C**), distal (**D**), and basal views (**E**). Scale bar equal to 1 cm

MCF-PVPH-920 and MCF-PVPH-930; Morphotype 1 (Fig. 2).

### State of preservation and general morphology

The recovered teeth are well preserved and are represented by shed crowns without their roots. Specimen MCF-PVPH-920 lacks most of its enamel. The denticles are not worn and show their original shape.

### Crown overall morphology

The teeth are ziphodont type with a labiolingually compressed crown, curved distally, strongly convex mesial margin, and a concave distal margin in lateral view. Labiolingual compression of the crown close to the cervix (CBR) is equal to 0.53 in MCF-PVPH-930 and 0.79 in MCF-PVPH-920, whereas at mid-crown (MCR) is equal to 0.53 and 0.86 in MCF-PVPH-930 and MCF-PVPH-920, respectively. The baso-apical elongation of the crown ratio (CHR) varies between 1.48 to 2.21. The mesial border shows a unserrated carina extending three-quarters apicobasally without reaching the cervix. This carina is curved lingually towards the base. On the distal view, the distal margin possesses a well-developed serrated distal carina, straight or very slightly bowed, and terminates beneath the cervix. The distal carina bears around 14/15 denticles per 5 mm at mid-crown and 19 denticles per 5 mm close to the cervix, whereas around 12/13 denticles per 5 mm are observed close to the apex. The shape of denticles on the distal carina is symmetrically convex to slightly asymmetrical with a parabolic margin. They are horizontal and subrectangular in shape, i.e., longer mesiodistally than apicobasal, and perpendicular to the distal margin. The interdenticular space is broad, whereas the interdenticular sulci, both at the mid- and the base crown, are long and well-developed. Lingual and labial surfaces exhibit a shallow median depression with a triangular shape in their basal half, with the lingual depression being more clearly defined and flanked by subtle ridges. Due to these depressions, the cross-section takes on a figure-eight shape near the level of the cervix. The crown shows arcuate transverse undulations. A braided enamel texture is observed. Marginal undulations, flutes, longitudinal grooves, or ridges (sensu [18]) are absent.

Megaraptoridae Novas, Agnolín, Ezcurra, Porfiri, and Canale, 2013.

Gen. and sp. indet.

### Material

**Fig. 3:**
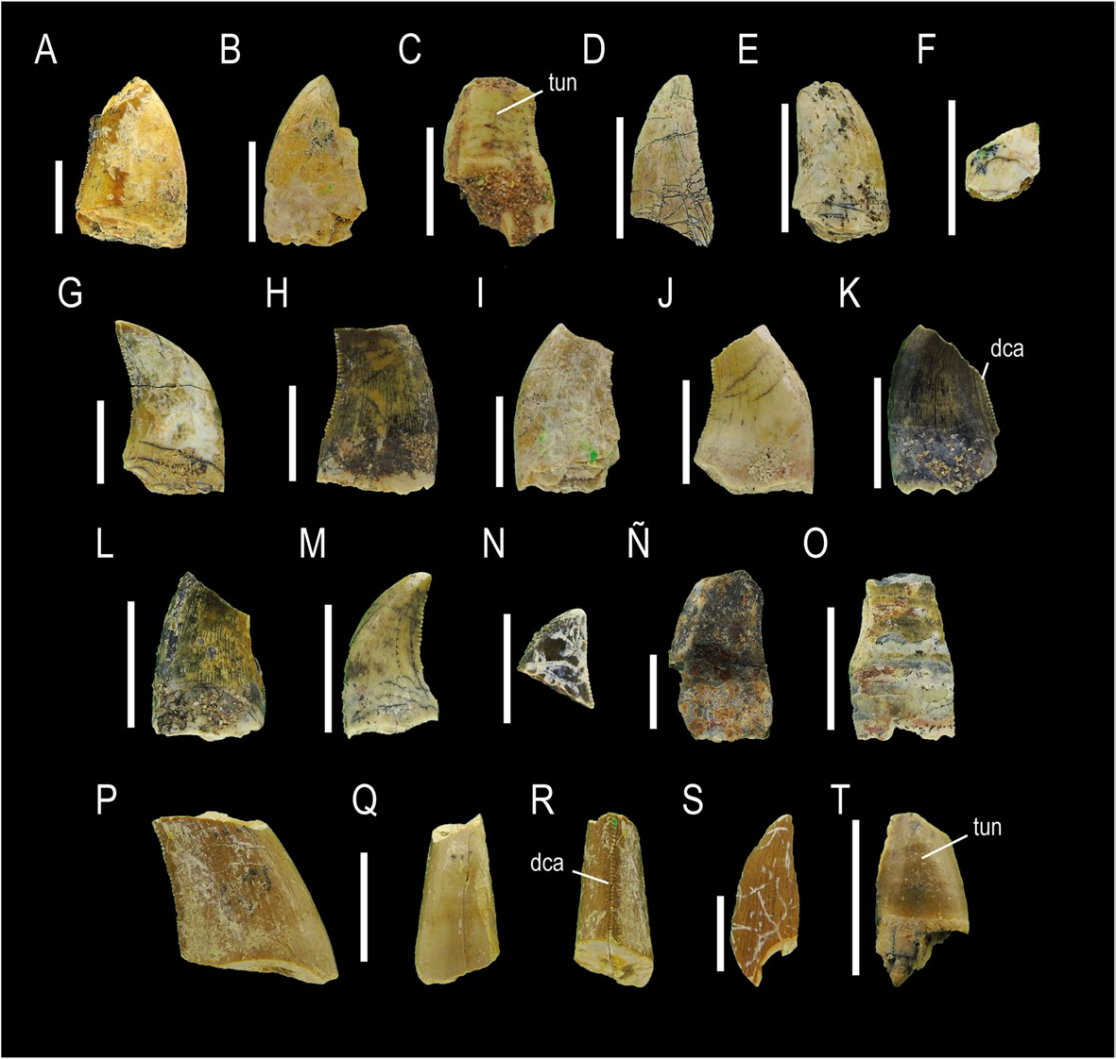
Megaraptorid teeth of Morphotype 2. MCF-PVPH-921, MCF-PVPH-922, MCF-PVPH-924 in labial views (**A-C**); MCF-PVPH-925 in indeterminate side (**D**); MCF-PVPH-929 in lingual view (**E**); MCF-PVPH-930 in indeterminate side (**F**); MCF-PVPH-933, MCF-PVPH-934, MCF-PVPH-935, MCF-PVPH-937, MCF-PVPH-938 in labial views (**G-K**); MCF-PVPH-939 and MCF-PVPH-940 lingual views (**L-M**); MCF-PVPH-941 indeteminate side (**N**); MCF-PVPH-944 lingual view (**Ñ**); MCF-PVPH-945 in mesiolabial view (**O**); MCF-PVPH-947 in labial (**P**), mesial (**Q**) and distal (**R**) views; MCF-PVPH-948 in indeterminate side (**S**); and MCF-PVPH-949 in labial view (**T**). Scale bar equal to 1 cm

MCF-PVPH-921, MCF-PVPH-922, MCF-PVPH-924, MCF-PVPH-925, MCF-PVPH-929, MCF-PVPH-930, MCF-PVPH-933, MCF-PVPH-934, MCF-PVPH-935, MCF-PVPH-937, MCF-PVPH-938, MCF-PVPH-939, MCF-PVPH-940, MCF-PVPH-941, MCF-PVPH-944, MCF-PVPH-945, MCF-PVPH-947, MCF-PVPH-948, and MCF-PVPH-949; Morphotype 2 (Fig. 3).

### State of preservation and general morphology

Two teeth MCF-PVPH-933 and MCF-PVPH-940 from the recovered sample are complete. Specimens MCF-PVPH-921/22, MCF-PVPH-933 and MCF-PVPH-929, MCF-PVPH-934/935, MCF-PVPH-937 and MCF-PVPH-933 and MCF-PVPH-944 are virtually complete, lacking some sectors of their crowns. The rest of teeth are represented by fragments of the crown where only one portion of its central zone is preserved.

### Crown overall morphology

Specimens asigned to this morphotype have a crown labiolingually compressed and distally recurved, with a strongly convex mesial margin, and a concave distal margin in lateral view (zhiphodont type). Both the labiolingual compression of the crown close to the cervix (CBR) and at mid- (MCR) varies in a range from 0.51 to 0.63. The baso-apical elongation of the crown ratio (CHR) varies between 1.63 to 1.68. Unlike Morphotype 1, this one lacks a mesial carina. The serrated distal carina is well-developed, straight or very slightly bowed, and terminates beneath the cervix. The density of denticles close to the cervix (15 to 22.5 per 5 mm; DB) is greater than the density of denticles at mid-crown (12 to 18 per 5 mm; DC). The density of denticles at the apex is equal than that at mid-crown. The shape of denticles on the distal carina is asymmetrically convex with a parabolic margin, and are longer mesiodistally than apicobasally, and perpendicular to the distal margin. The interdenticular space is narrow, whereas interdenticular sulci are not present as in the morphotype previously described. Like Morphotype 1, the lingual and labial surfaces exhibit a shallow median depression with a triangular shape in their basal half, with the lingual depression being more clearly defined and flanked by subtle ridges. Due to these depressions, the cross-section takes on a eight-shape near the level of the cervix. The crown shows well-marked arcuate transverse and marginal undulations. A braided enamel texture is observed.

Theropoda Marsh, 1881.

Ceratosauria Marsh, 1884.

Abelisauroidea Bonaparte, 1991.

Abelisauridae Bonaparte and Novas, 1985.

Gen. and sp. indet.

### Material

**Fig. 4:**
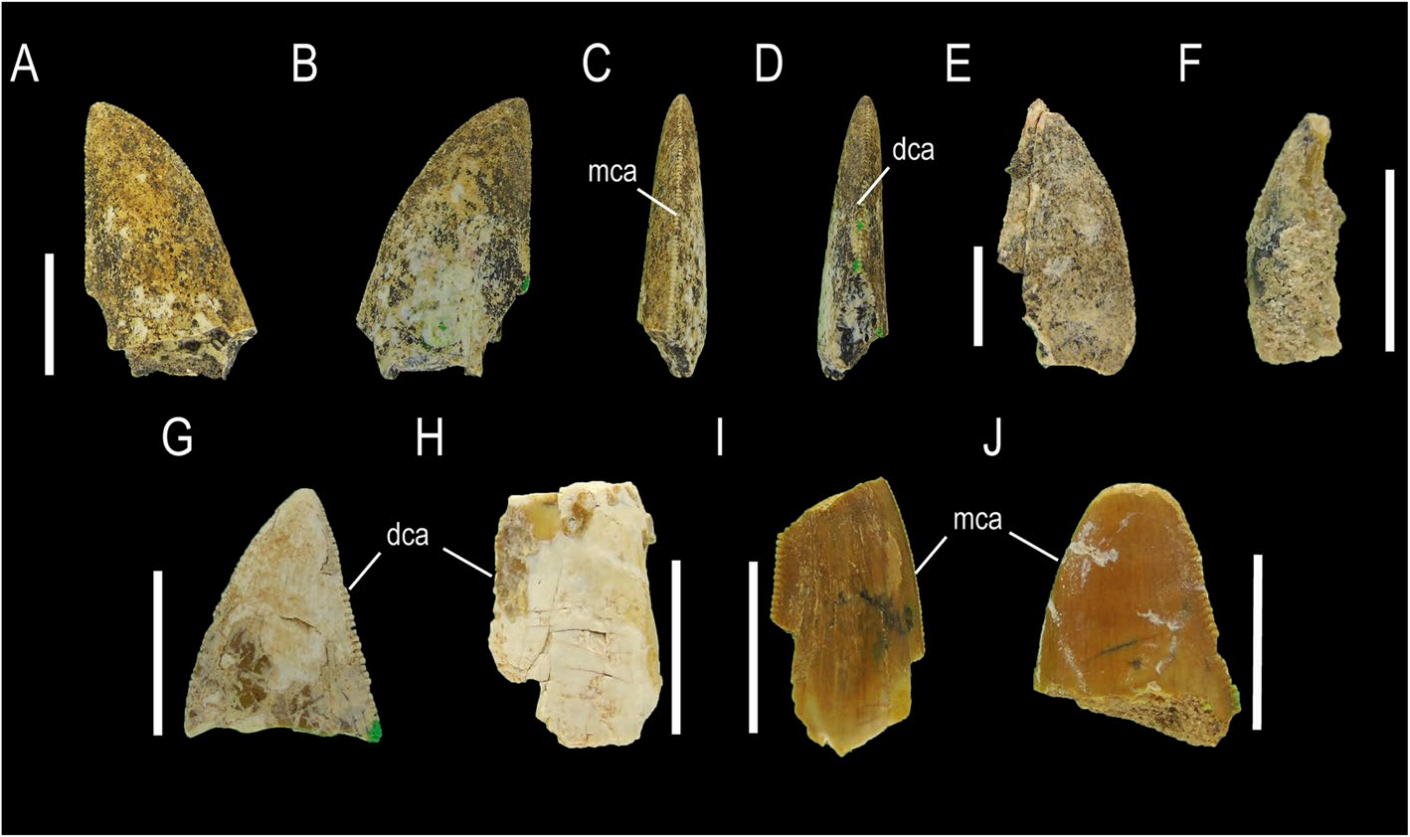
Abelisaurid teeth of Morphotype 3. MCF-PVPH-923 in lingual (**A**), labial (**B**), mesial (**C**), distal (**D**) views; MCF-PVPH-927 and MCF-PVPH-928 in labial views (**E–F**); MCF-PVPH-936 in lingual view (**G**); MCF-PVPH-946 in labial (**H**) view; MCF-PVPH-950 in labial view; and MCF-PVPH-951 in lingual view (**J**). Scale bar equal to 1 cm

MCF-PVPH-923, MCF-PVPH-936 and MCF-PVPH-946; Morphotype 3 (Fig. 4).

### State of preservation and general morphology

The collected crowns are quite well-preserved with MCF-PVPH-923 being the most complete. The remaining two lack their most basal portion (MCF-PVPH-936) or most apical portion (MCF-PVPH-946). All specimens described here, as well as all specimens studied, are considered to be shed teeth.

### Crown overall morphology

The crowns are strongly labiolingually compressed with an MCR equal to 0.52, and are slightly curved in an anteroposterior sense with a straight distal profile. Both lingual and labial surfaces are strongly convex. Both mesial and distal carinae are well-developed, having serrations centrally positioned along the margins. The mesial carina bears 16 denticles per 5 mm close to the cervix, 12/15 denticles per 5 mm at mid-crown, and 14/15 denticles per 5 mm in the apex of the crown. The distal carina possesses 12 denticles per 5 mm at mid-crown and 11/15 denticles per 5 mm close to the apex. The relationship between the number of mesial and distal denticles (DSDI) is 1. The denticles of the mesial carina are asymmetrical and inclined apically from the mesial margin, whereas the denticles of the distal carina also are asymmetrical but perpendicular to the distal margin. The interdenticular space is narrow in the mesial carina and broad in the distal one. The enamel texture is smooth and not oriented in any preferential direction. The cross-section is lenticular almost at the level of the cervix.

Theropoda Marsh, 1881.

Coelurosauria Huene, 1920.

Alvarezsauria Bonaparte, 1991.

Alvarezsauridae Bonaparte, 1991.

Gen. and sp. indet.

### Material

**Fig. 5:**
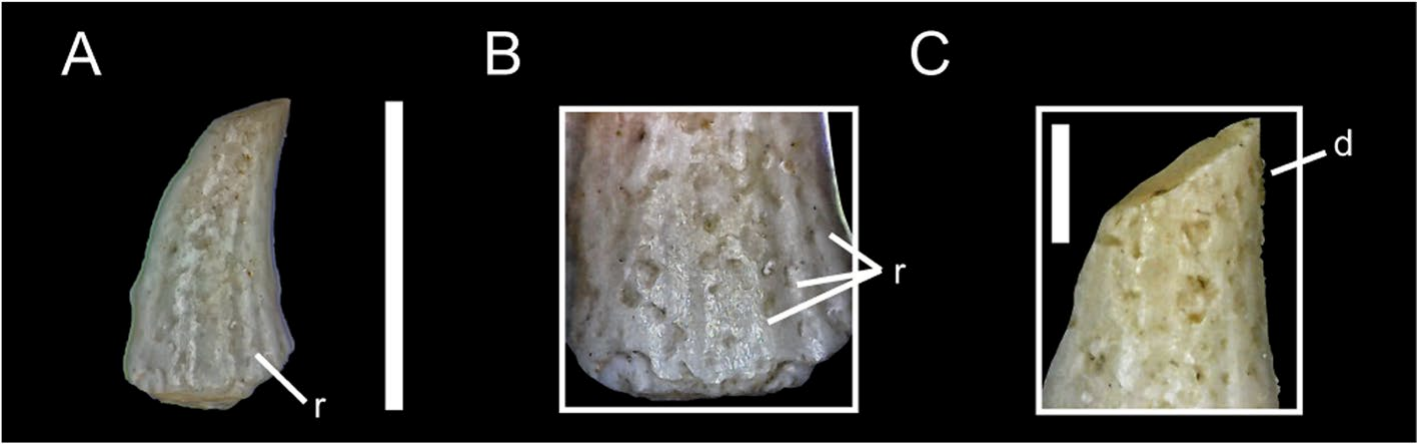
Alvarezsaurid tooth of Morphotype 4. MCF-PVPH-926 in labial (**A**) view; basal denticles of the mesial carina in lingual view (**B**), apical denticles of the distal carina in labial view (**C**). Scale bar equal to 5 mm and 1 mm

MCF-PVPH-926; Morphotype 4 (Fig. 5).

### State of preservation and general morphology

This specimen consists in a small crown that lacks the labial side and the most apical portion.

### Crown overall morphology

Although the labial side is not preserved, it can be inferred that its labiolingual compression (CBR) is weak, and the crown is slightly curved in an anteroposterior direction. In lateral view, its mesial margin is slightly convex, and its distal margin is slightly concave. The mesial and distal carina are present and serrated, although somewhat worn in some sectors. The mesial carina bears approximately 45 denticles per 5 mm close to the cervix, whereas the distal carina, accounts approx 77 denticles per 5 mm in the apex of the crown. The denticles in the mesial carina are asymmetrical and inclined apically from the mesial margin, whereas the denticles of the distal carina are asymmetrical and perpendicular to the distal margin. At least seven fainted ridges are observed about the basal zone of the crown. It is possible to observe in one sector, a constriction between the crown and the root. The enamel texture is smooth and not oriented in any preferential direction.

Theropoda Marsh, 1881.

Ceratosauria Marsh, 1884.

Abelisauroidea Bonaparte, 1991.

Abelisauridae Bonaparte and Novas, 1985.

Gen. and sp. indet.

### Material

**Fig. 6:**
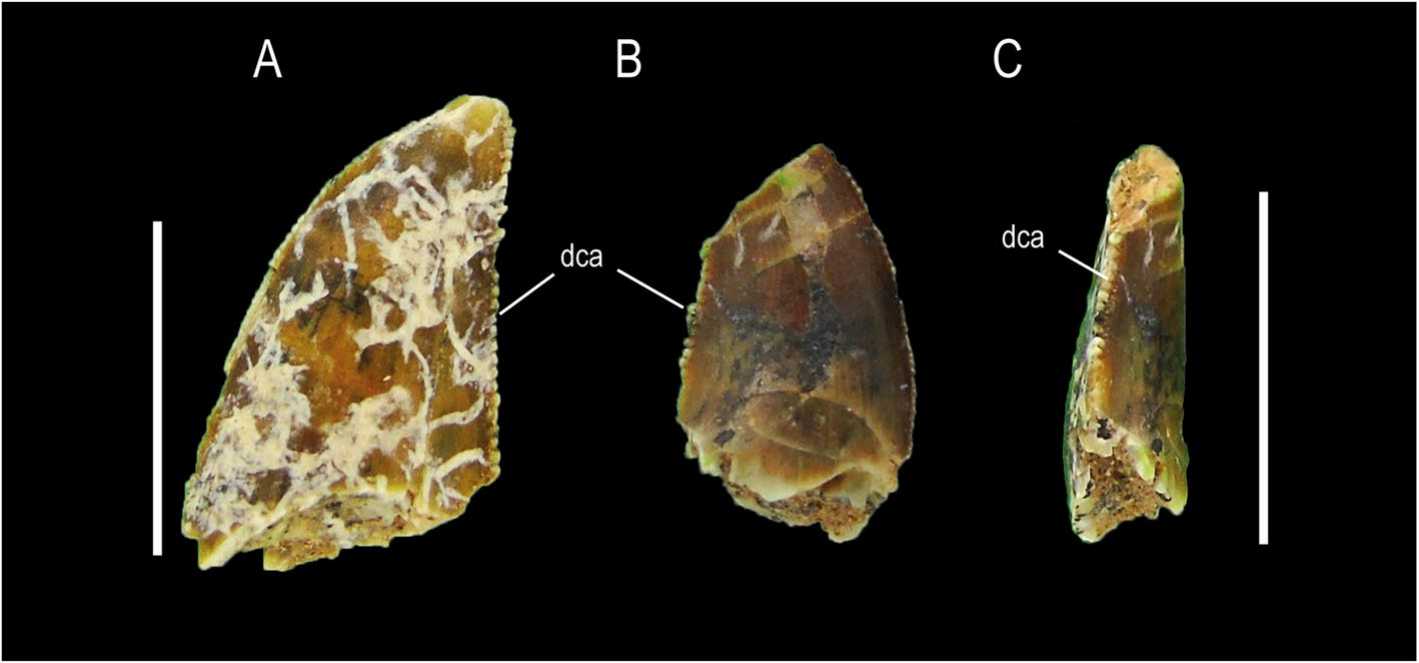
Abelisaurid teeth of Morphotype 5. MCF-PVPH-931 in labial view; and MCF-PVPH-932 in lingual (**B**) and distal (**C**) views. Scale bar equal to 1 cm

MCF-PVPH-931 and MCF-PVPH-932; Morphotype 5 (Fig. 6).

State of preservation and general morphology. Both specimens are well preserved, lacking some basal (MCF-PVPH-931) or apical (MCF-PVPH-932) sectors of the crown.

### Crown overall morphology

Both possess an important labiolingual compression close to the base of the crown (CBR = 0.44 to 0.52 and MCR = 0.45 to 0.46). Distally they are recurved with a strongly convex mesial margin and a straight distal margin in lateral view. The mesial and distal margins possess denticulatedcarinae. In the mesial carina, the density of denticles close to the cervix (27.5 per 5 mm; MB) is greater than the density of denticles at mid-crown (20 to 25 per 5 mm; MC). The density of denticles at the apex is equal to the density at mid-crown (20 to 25 per 5 mm; MA). In the distal carina, the density of denticles close to the cervix (12.5 per 5 mm; DB) is lower than the density of denticles at mid-crown (15 to 22.5 per 5 mm; DC). The density of denticles in the apex is greater than that at mid-crown. The shape of denticles on both carinae is asymmetrically convex with a parabolic margin, and are longer mesiodistally than apicobasal, and inclined from the mesial and distal margin. The interdenticular space is narrow, whereas the interdenticular sulci are present and well-developed. Also, a concave surface adjacent to the distal carina is observed. The outline of the basal cross-section of the crown is lenticular to lanceolate. The texture of the enamel is smooth and not oriented in any preferential direction.

Theropoda Marsh, 1881.

Ceratosauria Marsh, 1884.

Abelisauroidea Bonaparte, 1991.

Gen. and sp. indet.

### Material

**Fig. 7:**
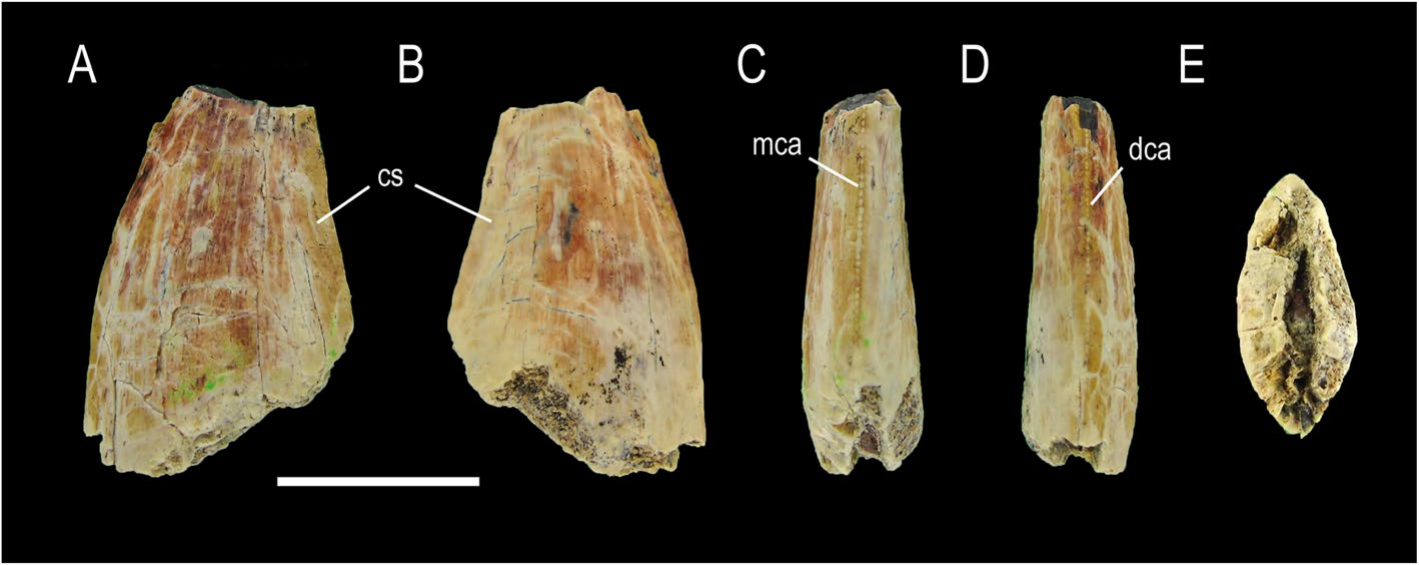
Abelisauroid tooth of Morphotype 6. MCF-PVPH-942 in lingual (**A**), labial (**B**), mesial (**C**), distal (**D**) and basal (**E**) views. Scale bar equal to 1 cm

MCF-PVPH-942, Morphotype 6 (Fig. 7).

### State of preservation and general morphology

This specimen lacks of its most basal and apical portions. The denticles are worn, so it is difficult to visualize their original shape.

### Crown overall morphology

The tooth is ziphodont type, slightly recurved distally with a convex mesial margin and a straight to slightly concave distal one. The MCR is 0.46, which implies a strong labiolingual compression at the level of the mid-crown. Both carinae are present, being denticulated, and centrally positioned along the mesial and distal margins. It is not possible to know whether the distal carina ends just above or below the cervix, although the mesial carina ends well above the cervix. The density of denticles is 2.4 per mm in both carinae. Adjacent to the distal carina, on the labial and lingual sides, there is a long apicobasally extended concave surface. Besides, the outline of the basal cross-section of the crown is lenticular to lanceolate in shape, and the texture of the enamel is smooth without any preferential direction.

### Cladistic analysis

**Fig. 8:**
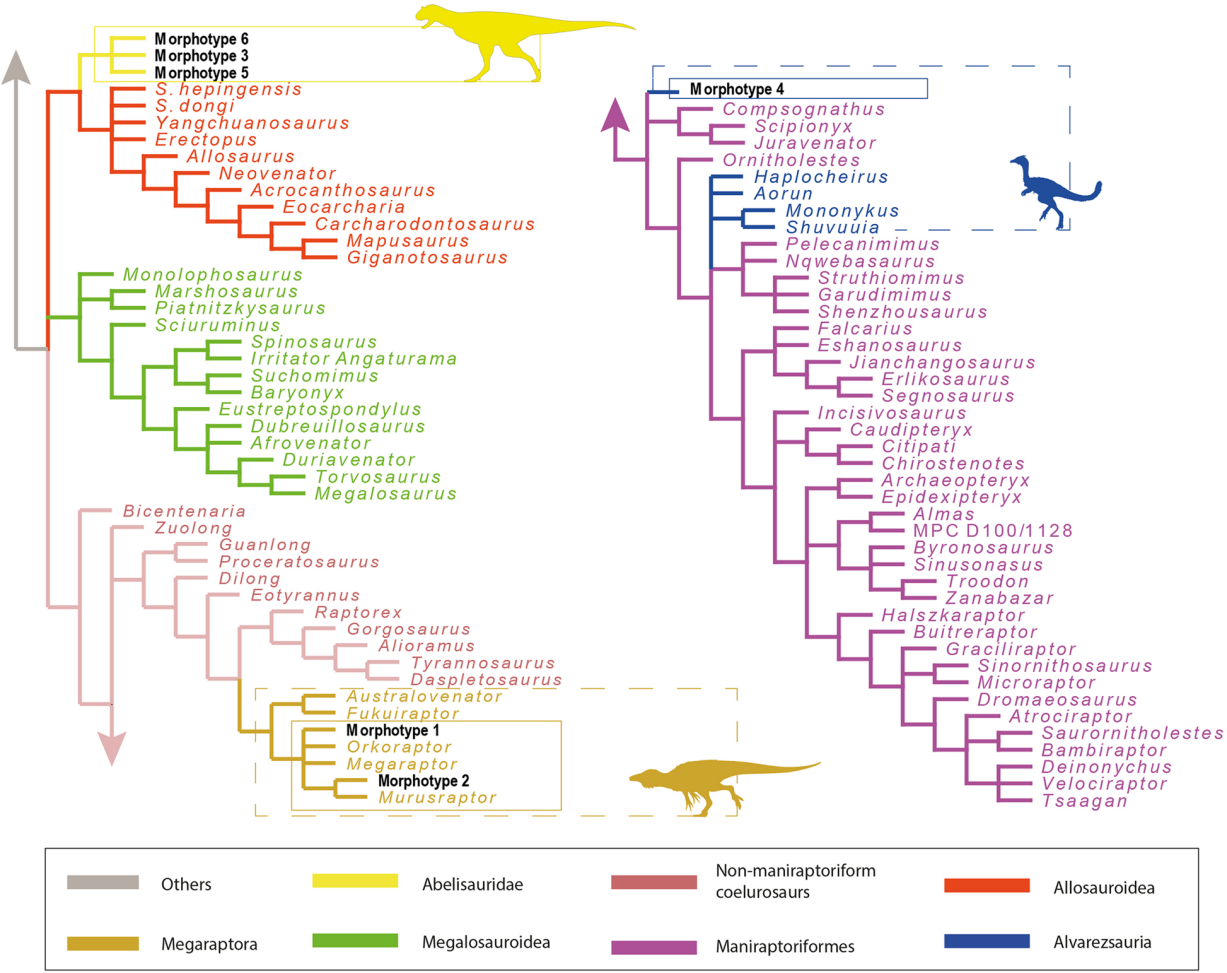
Strict consensus tree of the four most parsimonious trees (CI = 0.194; RI = 0.460; L = 1346 steps) was recovered in the cladistic analysis made from the dentition-based data matrix with constrained search and setting all morphotypes as floating terminals

The cladistic analysis performed from the dentition-based data matrix using a constrained tree topology recovered four most parsimonious trees (MPTs; CI = 0.194; RI = 0.460; L = 1346 steps). Morphotypes 1 and 2 are found within Megaraptoridae (Fig. 8). The latter is recovered as the sister taxon of *Murusraptor*, whereas morphotype 1 was either found in a small polytomy as the sister taxon of *Orkoraptor* or *Megaraptor* (Fig. 8). Morphotypes 3, 5, and 6 are recovered within a small subclade as the sister taxa of Allosauroidea (Fig. 8). Morphotype 4 is recovered as a early-diverging member of Neocoelurosauria (Fig. 8).

**Fig. 9:**
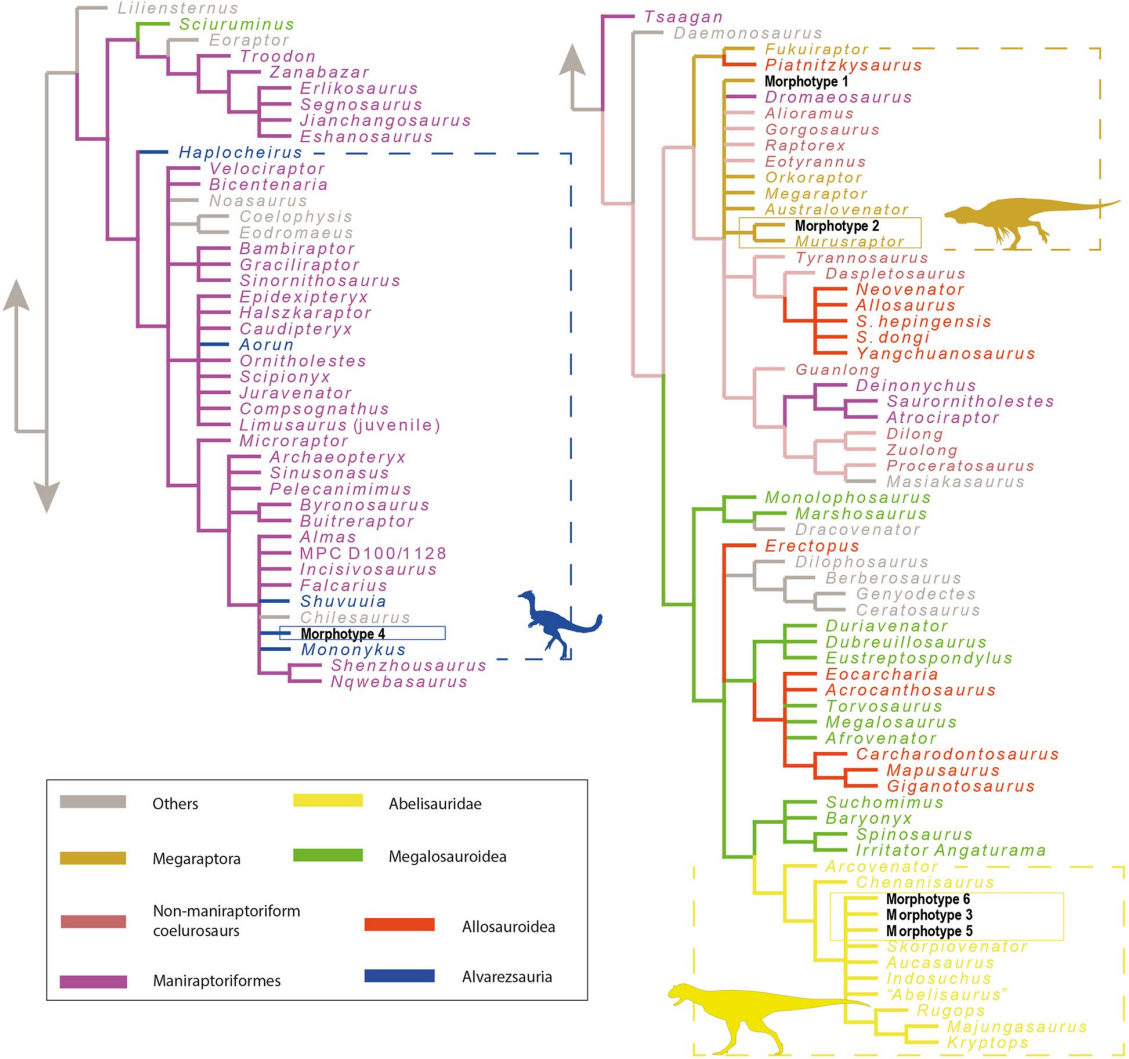
Strict consensus tree of the hundred most parsimonious trees (CI = 0.239; RI = 0.587; L = 1090 steps) recovered in the cladistic analysis made from the dentition-based data matrix with an unconstrained search

The cladistic analysis performed with no constraint found more than a hundred most parsimonious trees (CI = 0.239; RI = 0.587; L = 1090 steps). In the strict consensus tree, Morphotype 2 is recovered as the sister taxon of *Murusraptor*, whereas Morphotype 1 is recovered within a large polytomy together with some megaraptorids, maniraptoriforms, tyrannosauroids, and allosauroids (Fig. 9). This is because Morphotype 1 is recovered as the sister taxon of *Orkoraptor*, which in turn is recovered as a later-diverging tyrannosaurid. Morphotypes 3, 5, and 6 are recovered within Abelisauridae in a polytomy with the brachyrostran forms (Fig. 9). Finally, Morphotype 4 is recovered in a small polytomy formed by some ornithomimosaurians, oviraptorosaurians, therizinosaurians, *Mononykus*, *Shuvuuia*, and *Chilesaurus* (Fig. 9).

**Fig. 10:**
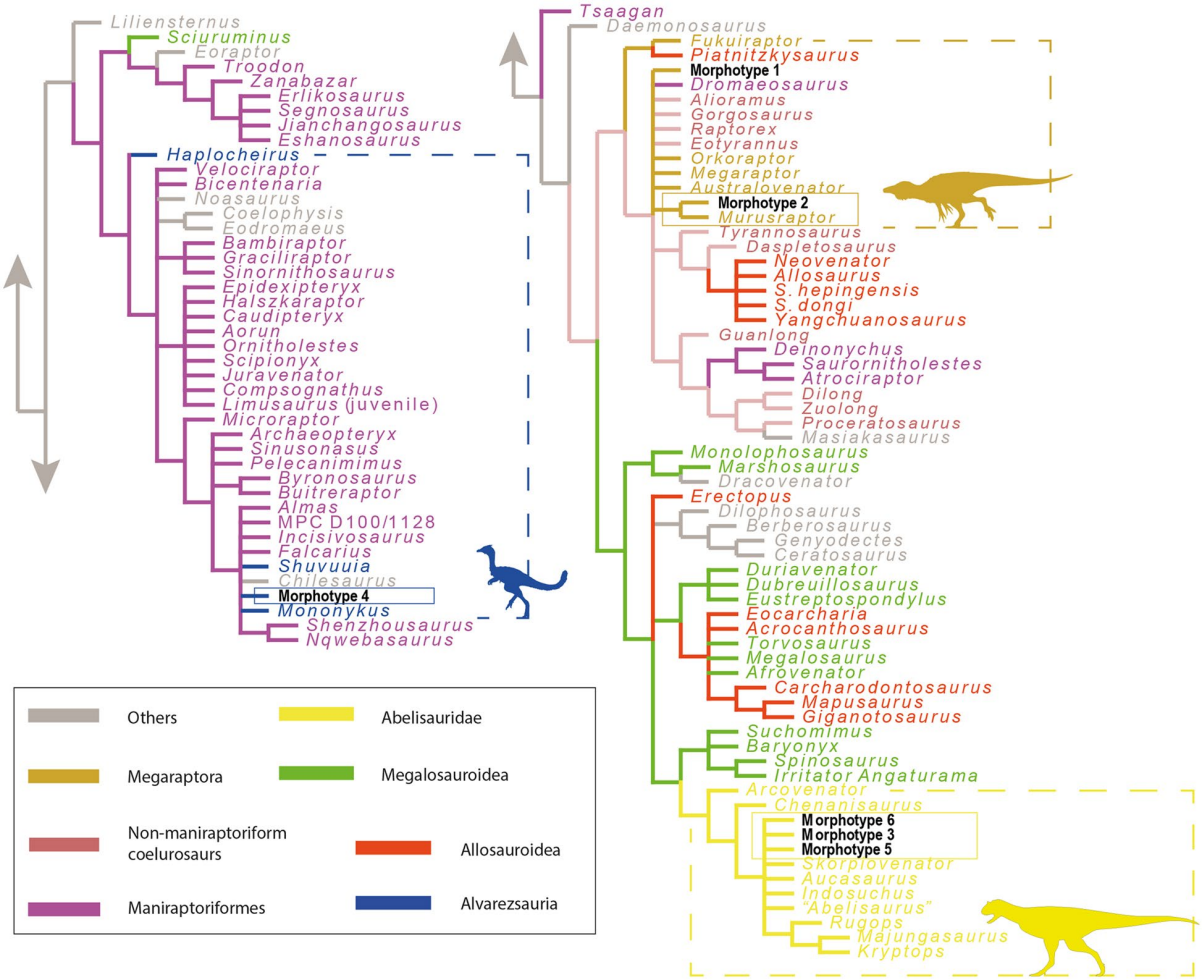
Strict consensus tree of the hundred most parsimonious trees (CI = 0.240, RI = 0.630, L = 667 steps) was recovered in the cladistic analysis made from the tooth-crown-based data matrix

Regarding the cladistic analysis conducted on the crown-based data matrix with no constraints, we found a hundred MPTs (CI = 0.240, RI = 0.630, L = 667 steps), and the strict consensus tree recovered a topology very similar to the previous analysis (Fig. 10).

### Discriminant analysis

**Fig. 11:**
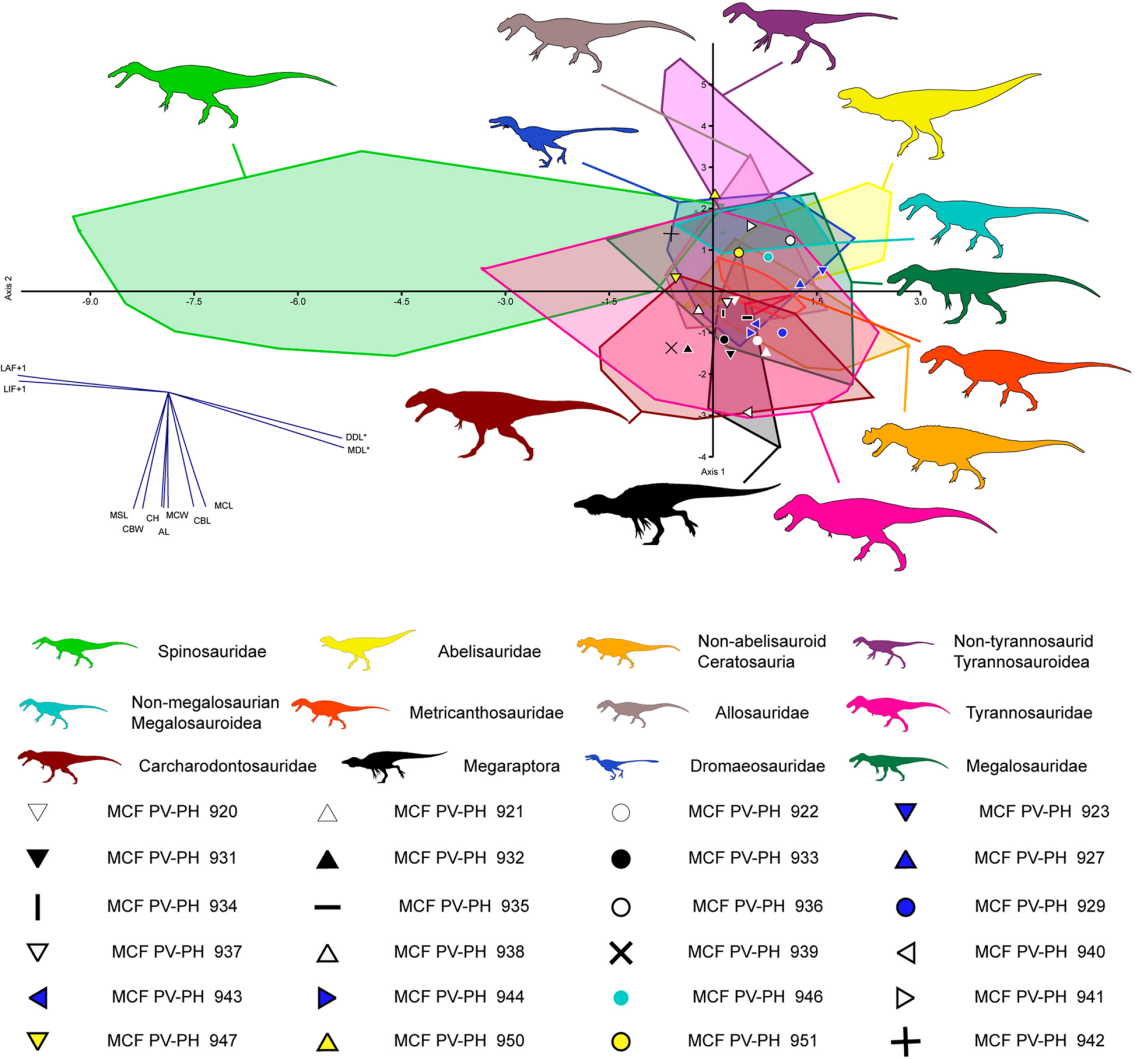
Results of the discriminant analysis performed at the clade-level from the whole dataset with personal measurements of Christophe Hendrickx on 400 teeth belonging to 46 theropod taxa and 12 groupings along the first two canonical axes of maximum discrimination in the dataset (PC1 40.34% and PC2 27.36% of the total variance, respectively)

The Discriminant Function Analysis (hereafter referred to as DFA), carried out on the entire dataset, categorized shed-isolated teeth corresponding to Morphotype 1 as belonging to *Neovenator* and tyrannosaurids. Meanwhile, Morphotype 2 was identified among megaraptorids (Fig. 11). Morphotypes 3, 5, and 6 were classified as abelisaurids and therizinosaurids in the clade-level analysis, with PC1 and PC2 accounting for 48.13% and 19.86% of the total variance, respectively (see Supplementary Information 3). At the taxon level, most teeth were found to be associated with each other. Only one tooth of Morphotype 1 (MCF-PVPH-920) was classified with *Zhuchengtyrannus*, while Morphotype 2 (MCF-PVPH-939) was associated with *Australovenator*. The same pattern emerged for Morphotype 3 (MCF-PVPH-923), closely related to *Aucasaurus* (PC1 and PC2 accounted for 41.28% and 20.69% of the total variance, respectively; see Supplementary Information 3). In both clade-level and taxon-level analyses, the reclassification rate (hereafter RR) was found to be low, at 58.84% and 57.73%, respectively. The reclassification rate slightly improved in the DFA performed with the dataset where absent denticles were coded as inapplicable, reaching 58.12% at the clade level and 56.5% at the taxon level. In these analyses, isolated teeth were consistently classified within the same groups (clade-level: PC1 and PC2 accounting for 47.01% and 18.9%; taxon-level: PC1 and PC2 accounting for 41.76% and 15.84%; see Supplementary Information 3).

The DFA carried out using the whole dataset of [24] based on first-hand measurements, classified the isolated teeth as carcharodontosaurids, abelisaurids, dromaeosaurids and troodontids (clade-level analysis; PC1 and PC2 account for 49.66% and 24.22% of the total variance, respectively; Supplementary information 3). At the taxon-level (PC1 41.24% and PC2 19.14%), the shed teeth were found closely related to members of carcharodontosaurids, abelisaurids, and megaraptorids. The RR is better at the taxon-level (58.69%) than at the clade-level (54.42%). The DFA made when the absence of denticles was coded as inapplicable in dataset resulted in the teeth being grouped with carcharodontosaurids, abelisaurids, dromaeosaurids, tyrannosaurids, and troodontids (clade-level analysis; PC1 and PC2 account for 40.21% and 27.66% of the total variance, respectively; Supplementary information 3). In turn, at taxon-level, these were found closely related to members of carcharodontosaurids, abelisaurids, megaraptorids, and megalosaurids (PC1 34.68% and PC2 21.34%). The RR is slightly higher in clade-level (55.03%) and slightly lower in the taxon-level (55.34%).

In the DFA performed on the datasets restricted to taxa with teeth larger than two centimeters (i.e., the whole dataset of Hendrickx’s first-hand measurements), the isolated teeth are classified as megaraptorids, abelisaurids, megalosaurids, non-megalosauranmegalosaurids, tyrannosaurids, and non-tyrannosauroidtyrannosaurids (clade level; PC1 40.34% and PC2 27.36%; Fig. 11). At taxon-level, the teeth are grouped with the megaraptorids, abelisaurids, non-abelisauroid abelisaurids, and tyrannosaurids clades (taxon-level; PC1 50% and PC2 17.29%). The RR is 56.47% at clade-level and 63.39% at taxon-level, a percent higher than anterior analyses. In the DFA performed when the absence of denticles is considered inapplicable in the dataset, the same results were recovered, although the RR is 57.81% at the clade-level and 63.17% at the taxon-level.

**Fig. 12:**
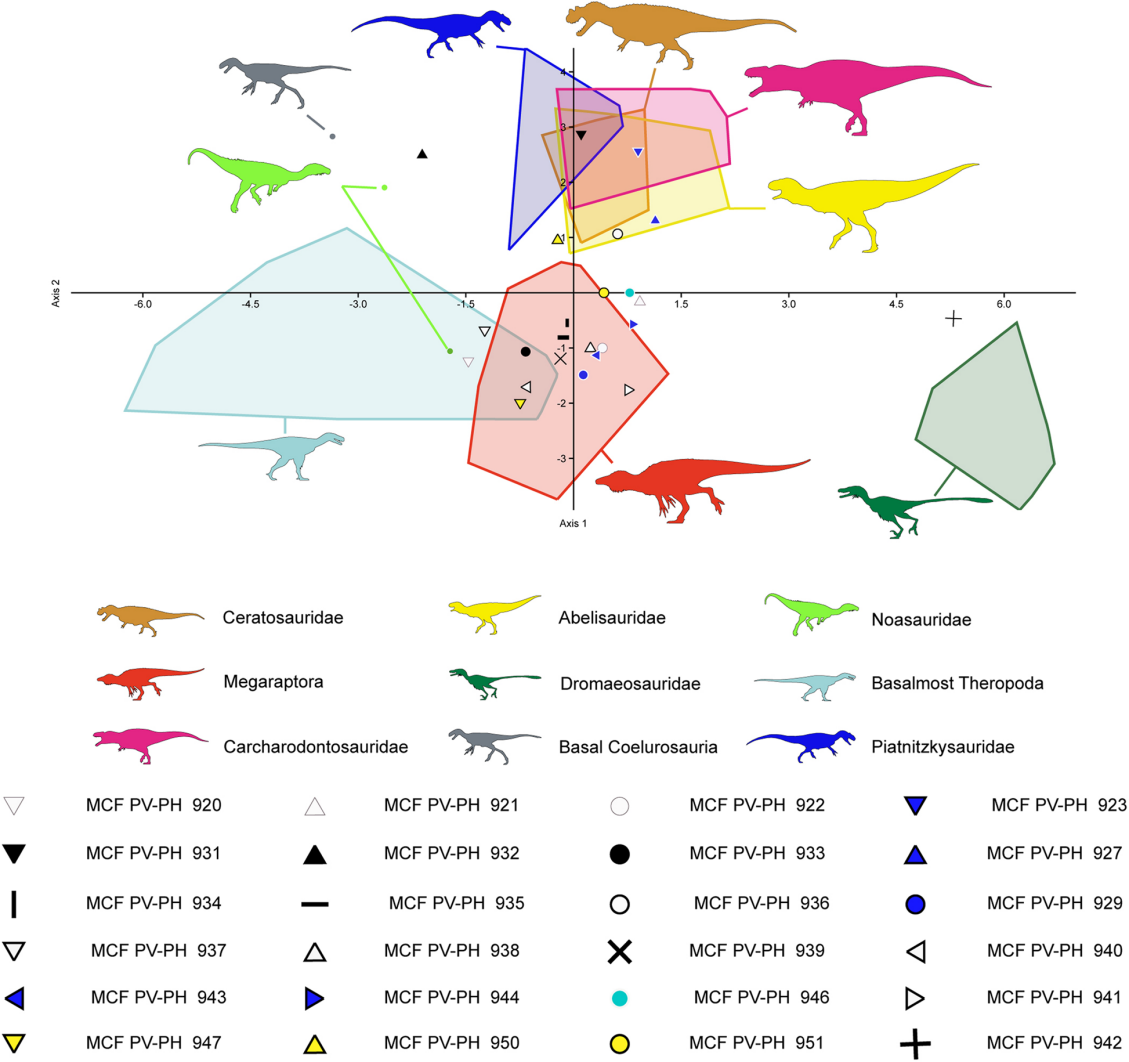
Results of the discriminant analysis performed at the clade-level from the Argentinean taxa dataset with personal measurements of C. H. and J. M. on 99 teeth belonging to 46 theropod taxa and 9 groupings along the first two canonical axes of maximum discrimination in the dataset (PC1 48.63% and PC2 28.76% of the total variance, respectively)

In the DFA conducted on the datasets restricted to Argentine taxa, the different morphotypes are classified as megaraptorids, abelisaurids, non-abelisauroid ceratosaurians, and carcharodontosaurids (clade level; PC1 48.63% and PC2 28.76%; Fig. 12). At taxon-level, the results of the DFA recovered the same results. The RR increased drastically, being 80.25% at the clade-level and 79.63% at the taxon-level.

### Cluster analysis

The cluster analysis, conducted on datasets limited to taxa with teeth larger than two centimeters and employing the hierarchical clustering option, identified the isolated teeth as belonging to neovenatorids, carcharodontosaurids, megaraptorids, megalosaurids, abelisaurids, and tyrannosaurids (See Supplementary information 3). Similarly, the cluster analysis carried out on the dataset where the absence of denticles is deemed inapplicable yielded a classification akin to the previous analysis (see Supplementary information 3).

The cluster analysis, employing the neighbor-joining option, identified the shed teeth as members of neovenatorids, carcharodontosaurids, megaraptorids, megalosaurids, abelisaurids, allosaurids, and tyrannosaurids. When utilizing the dataset where the absence of denticles is considered inapplicable, the results remained consistent, albeit with minor variations in the composition of its members (see Supplementary information 3).

## Discussion

### Cladistics and multivariate analyses

In the cladistics analyses that shows the morphotypes 1 and 2 nested inside the Megaraptoridae subclade, the result is supported by the following synapomorphies: 1) lateral teeth with labial and lingual depressions at the bases of the crowns, giving the crowns a 8-shaped basal cross-section; 2) a mesial carina absent in the lateral teeth. The first feature is also present in *Berberosaurus*, Metriacanthosauridae, Tyrannosauroidea, Troodontidae, and most Dromaeosauridae [19, 42]. The second characteristic is observed in Morphotype 2 but is present in Morphotype 1, albeit with an unserrated carina. Taxa exhibiting an unserrated mesial carina and a serrated distal carina in lateral dentition include *Sciurumimus*, certain megaraptorans, *Ornitholestes*, Compsognathidae, basal Alvarezsauria, some Dromaeosauridae, and many Troodontidae [19, 42].

The synapomorphies that group Morphotypes 3, 5, and 6 as sister taxa of some allosauroids are: 1) mesial teeth with a salinon-shaped cross-section, with labial margin convex and lingual margin biconcave; 2) subquadrangular (i.e., as long mediodistally as apicobasally) mesial denticles at two-thirds of the crown in lateral teeth; 3) interdenticular sulci present between mid-crown denticles on the distal carina in lateral teeth; and 4) enamel surface texture of the crown is smooth or irregular. The first character is present in some abelisauroids, allosaurids, and troodontids (e.g., [24, 42]). Character 2 is widely distributed in theropods, as mentioned by [42]. Character 3 is present in piatnitzkysaurids, megalosaurids, and allosaurids (see [42]). Character 4 is present in Abelisauroidea, Metriacanthosauridae, and Neocoelurosauria [42]. Regarding synapomorphies that recovered Morphotypes 3, 5, and 6 as abelisaurids are: 1) a salinon to J-shaped cross-section outline at the crown base, in mesial teeth; 2) similar or lower number of denticles at the apex than at mid-crown; 3) strongly developed interdenticular sulci; 4) well-visible marginal undulations; 5) an irregular enamel surface texture; 6 and 7) crowns that are short and strongly compressed, indicated by a CBR (Crown Base Ratio) of less than 0.5 and a CHR (Crown Height Ratio) of less than 2; 8) a mesial carina extending towards the cervix; 9) a distal carina centrally positioned on the distal margin of the crown; 10) elongated interdenticular sulci; 11) An irregular enamel surface texture. Characters 1–5 are some of those always recovered as synapomorphies in previous papers when mesial teeth (e.g., [19, 24, 38, 42, 43]). Similar to mesial teeth, characters 6–11 have also been identified as synapomorphies for the lateral dentition of abelisaurids [19, 22, 24, 38, 43].

The position of Morphotype 4 as an early-diverging member of neocoelurosaurians is supported by: 1) presence of denticles on the mesial carina at two-thirds of the crown's height, with a density exceeding 30, in lateral teeth. This characteristic is exclusively found in some early-diverging theropods, noasaurids, some spinosaurids, *Bicentenaria*, *Aorun*, *Haplocheirus*, *Falcarius*, and some dromaeosaurids. 2) the enamel surface texture of the crown is either smooth or irregular. Regarding its position in a small polytomy shared by some ornithomimosaurians, oviraptorosaurians, therizinosaurians, derived alvarezsaurids, and *Chilesaurus*, is supported by: 1) weak constriction between root and crown. This character is observable in all these mentioned forms [42].

So far, and as previously noted in the literature, megaraptorid synapomorphies are limited but are highly diagnostic [19, 44]. In the specific context of this study, two out of three analyses suggest that Morphotype 1 lacks strong affinities with the Megaraptoridae subclade. This divergence is likely attributed to specific characteristics, which will be addressed in subsequent discussions (see below). Regarding to the dental synapomorphies recovered for Abelisauridae, these are numerous and highly diagnostic [19, 24, 38, 39, 42, 43]. The case of Morphotype 4 is intriguing, as despite not demonstrating a robust affinity with a specific subclade, it can be tentatively classified as an alvarezsaurid rather than an unenlaginae (see below).

The multivariate analyses produced mixed results (see Supplementary information 3), but all indicate that Morphotype 1 and 2 shows strong affinities with the Megaraptoridae subclade. This same occurs with Morphotypes 3, 5, and 6, and although they showed some variations, relating to other clades, most analyses support strong affinities with the dentition of abelisaurids. This discrepancy may be linked to the significant absence of metric measurements for numerous teeth, resulting in varied outcomes. Consequently, a decision was made to conduct an analysis based on Argentine forms only, aiming to observe their distribution in morphospace. This approach substantially clarified the classification of isolated teeth, with only a few specimens, namely MCF-PVPH-921, 932, 942, and 946 remaining uncertain. Such uncertainty can be attributed to two factors: 1) incomplete data entry, and 2) limited documentation of teeth from diverse Argentine species, hindering a comprehensive exploration of morphospace.

### Morphological comparison of the teeth from Sierra del Portezuelo

The specimen MCF-PVPH-925 is categorized with an indeterminate anatomical placement. Conversely, MCF-PVPH-927 and potentially MCF-PVPH-945 are identified in this context as part of the mesial dentition, supported by their metrical dimensions, asymmetrical labial and lingual surfaces, and the outline of their cross-section. The rest of the specimens are considered to belong to the lateral dentition based on their labiolingual compression and their symmetrical shape [18].

In Morphotype 1, a mesial unserrated carina is developed just before the cervix. In some lateral teeth of *Megaraptor* and *Murusraptor*, the presence of a mesial carina is limited to the apical third of the crown ([45] and pers. obs.). This morphology could be regarded as a transitional feature between early-diverging megaraptorans such as *Fukuiraptor* and the rest of megaraptorids. However, while *Australovenator* exhibits a denticulated mesial carina, this feature is confined to the apical third [46]. This introduces uncertainty into the evolution of this characteristic and likely signifies a more intricate paleogeographic distribution for this clade. The identified morphology may lend support to the existence of a second megaraptorid morphotype within the Portezuelo Formation and the Sierra del Portezuelo locality. Regarding Morphotype 2, it does not draw significant attention as it demonstrates a clear affinity with the dental characteristics recognized in Patagonian megaraptorids [19]. In both *Murusraptor* and *Megaraptor*, some teeth may or may not have a small development of a mesial carina [45, 47]. Consequently, assuming that Morphotypes 1 and 2 may belong to the same megaraptorid species, our studies propose the presence of a new megaraptorid taxon. In any case, Morphotype 1 implies the existence of a new megaraptorid for the locality and formation. Morphotypes 3 and 5 display features consistent with both the mesial and lateral dentition of abelisaurid teeth, a observation elucidated in previous studies [24, 38, 42, 43]. Morphotype 6, despite being an incomplete crown, is notable for a distinctive feature—the number of denticles per 5 mm. Unlike Morphotypes 3 and 5, this Morphotype is characterized by having a density of 2.4 denticles per mm. Such a low denticle density is typically observed in basal abelisauroids [24]. In the Sierra del Portezuelo locality of the Portezuelo Formation, a humerus is described that exhibits affinities with basal abelisauroids like *Masiakasaurus* [9]. However, it is worth noting that this taxon typically has a higher denticle density [42]. Only *Ceratosaurus*, *Indosuchus*, *Chenaniasaurus*, *Majungasaurus*, *Torvosaurus*, and *Vespersaurus* exhibit a MC and DC of less than 12 denticles per 5 mm [24, 42, 48].

Morphotype 4 poses a significant challenge, as its fragmentary state prevented its inclusion in morphometric analyses and a complete scoring of it in the cladistics matrix. However, in this last analyses, it is grouped with parvicursorine alvarezsaurids, ornithomimosaurians, oviraptorosaurians, and therizinosaurians. The latter three groups are easily ruled out, as there is no evidence of their presence in Patagonia. The only two small body-size clades recorded in the Sierra del Portezuelo locality within the Portezuelo Formation are Unenlagiinae and Alvarezsauridae. Unenlagiine teeth typically exhibit characteristics such as having a crown that is unserrated, strongly distally recurved, and often bearing longitudinal ridges or flutes [38, 42, 49]. Some unenlagiines also possess conidont type teeth, like *Austroraptor*, or strongly compressed lateral teeth with an 8-shaped cross-section outline, as seen in *Buitreraptor* [42, 49]. Consequently, the tooth in question can be ruled out as belonging to an unenlagiine dromaeosaurid due to the presence of tiny denticles, the absence of longitudinal flutes, and the lack of an 8-shaped cross-sectional contour. On the other hand, later-diverging alvarezsaurids dentition typically consists of tiny (< 1 cm) folidont and unserrated crowns [42, 50–52]. In the case of Patagonian forms, our knowledge is limited to a single specimen currently under study that preserves teeth [53]. This comparison is inherently biased due to the significant chronological gap of over 70 million years between the early-diverging forms of the Late Jurassic and the later-diverging forms of the Late Cretaceous. Nevertheless, certain features are discernible that might signify a transitional nature, as the Patagonian forms appear to exhibit features of transitional forms [52, 54, 55]. For instance, a ziphodont type crown with a denticulate carina and a high density of denticles is unique to *Haplocheirus* and *Aorun*, while Parvicursorinae is characterized by folidont crowns and the absence of carinae [42, 54, 56]. The presence of a weak constriction is a common trait among all alvarezsaurians, and small longitudinal ridges are observed in *Haplocheirus*, *Aorun*, *Shuvuuia*, and *Mononykus*, but are notably absent in *Jaculinykus* [57]. If our interpretation is accurate, this suggests the presence of a second alvarezsaurid morphotype within the Portezuelo Formation. This aligns with occurrences in other alvarezsaurid-bearing formations such as *Shishugounykus* [58], *Haplocheirus* [56], *Aorun* [59], *Alvarezsaurus* [60], *Achillesaurus* [61], *Mononykus* [50], *Nemegtonykus* [62], *Shuvuuia* [51], *Kol* [63], *Ceratonykus* [64], *Ondogurvel* [65], *Khulsanurus* [66], and *Parvicursor* [67] that show coexistence of contemporaneous species. Although Morphotype 4 is recovered with forms predominantly from Laurasia, the evidence suggests that alvarezsaurid teeth could have great diagnostic potential despite the large number of missing entries in the matrix for this morphotype. However, at the moment, the dentition of Patagonian alvarezsaurids is quite biased and future discoveries will help to further understand the morphology of the Patagonian forms.

Although it has been observed that the dentition of several species of theropod dinosaurs varies throughout ontogeny, studies have demonstrated that there is a set of morphological characters that remain consistent between juvenile and adult individuals (e.g., [31, 42, 68, 69]). This ontogenetic variation can often create challenges in identifying isolated teeth, as those from immature individuals may resemble those of distantly related taxa due to similar diets or heterochronic processes (e.g., [31, 42]). In this context, tooth morphology, denticles shape, and density are characteristics that persist between juvenile and adult individuals, with some displaying highly diagnostic dentition for identification purposes [42, 70, 71]. Therefore, it can be argued that the morphology of the denticles between Morphotypes 3 and 5 represent two distinct species of abelisaurids, whereas the density and morphology of the denticles in Morphotype 6 differ from Morphotypes 3 and 5, suggesting affinities with a basal abelisauroid. In juvenile *Daspletosaurus* specimens, the carinae are unserrated, indicating ontogenetic and/or ecological changes [42, 72]. This example raises uncertainties regarding whether Morphotypes 1 and 2 represent different species or individuals of the same species at different ontogenetic stages. Unfortunately, there is currently no ontogenetic series of any megaraptorid specimen that can clarify this character variation. However, the dentition of a juvenile specimen of *Megaraptor namunhuaiquii* [45] and a subadult/adult specimen of *Murusraptor barrosaensis* [47, 73] show no variations in dental morphology, indicating potential stability in dentition within this clade. Nevertheless, future discoveries of complete ontogenetic series will provide further evidence supporting these ideas.

## Conclusions

Cladistic, discriminant, and cluster analyses of thirty-two shed crowns from the middle Turonian to late Coniacian Portezuelo Formation at the Sierra del Portezuelo locality has revealed six tooth morphotypes identified as Megaraptoridae (Morphotype 1 and 2), Abelisauridae (Morphotype 3 and 5, exhibit a combination of unequivocal dental features commonly found in the mesial and lateral dentition of megaraptorid and abelisauridtheropods, respectively), Abelisauroidea (Morphotype 6), and Alvarezsauridae (Morphotype4). Based on our results, it can be inferred tentatively that a second previously undocumented tooth morphotype of a megaraptorid and alvarezsaurid could be present in this formation increasing the theropod diversity in the original ecosystem. In the case of Morphotype 6, this could signify a morphotype closely related to a member of a medium-sized abelisauroid that coexisted with larger abelisaurids in the original ecosystem. These results inspire future efforts to undertake further expeditions to the Sierra del Portezuelo locality to learn more about these previously unknown theropod species.

### Supplementary Information

Supplementary Material 1.

Supplementary Material 2.

Supplementary Material 3.

## Data Availability

No datasets were generated or analysed during the current study.

## Abbreviations

MCF-PV PH: Museo “Carmen Funes”, Plaza Huincul, Neuquén, Argentina
AL: Apical length
CBL: Crown base
CBR: Crown base ratio
CBW: Crown base width
CH: Crown height
CHR: Crown height ratio
CI: Consistency index
CTU: Crown transverse undulation density
DA: Disto-apical denticle density
DAVG: Average distal denticle density
DB: Disto-basal denticle density
DC: Disto-central denticle density
DDT: Dentine thickness distally
DFA: Discriminant function analysis
DLAT: Dentine thickness labially
DLIT: Dentine thickness lingually
DMT: Dentine thickness mesially
DSDI: Denticle size density index
FABL: Fore-aft basal length
L: Length
LAF: Number of flutes on the labial surface of a crown
LIF: Number of flutes on the lingual surface of a crown
MA: Mesio-apical denticle density
MAVG: Average mesial denticle density
MB: Mesio-basal denticle density
MC: Mesio-central denticle density
MCE: Mesial carina extent
MCL: Mid crown length
MCR: Mid crown ratio
MCW: Mid-crown width
MDE: Mesio-basal denticles extent
RI: Retention index
