# Supplementary material for: Shed teeth from Portezuelo formation at Sierra del Portezuelo reveal a higher diversity of predator theropods during Turonian-Coniacian times in northern Patagonia
Source: BMC Ecol Evol. 2024 May 10;24:59. doi: 10.1186/s12862-024-02249-8 (PMC11083846; doi:10.1186/s12862-024-02249-8)
Supplement: Supplementary file 1 — Supplementary Material 1. [file 12862_2024_2249_MOESM1_ESM.docx]

Supplementary information 1

## Dentition-based Character List

I. PREMAXILLA ALVEOLI/TEETH

1. Premaxillary teeth (**Ordered**; Modified from Russell and Dong (1993) #2):

(0) present in the anterior and posterior portions of the premaxilla

(1) absent in the posterior portion of the premaxilla

(2) absent in the anterior portion of the premaxilla

(3) absent in the whole premaxilla, toothless premaxilla

2. Number of premaxillary teeth (or alveoli) (**Ordered**; Modified from Harris (1998) #47; Sereno et al. (1998) #19):

(0) 3 or less

(1) 4

(2) 5

(3) 6

(4) 7 or more

3. Premaxillary alveoli, direction of main axis of elongation in palatal view (Unordered; Hendrickx and Mateus (2014) #3):

(0) all alveoli mesio-distally oriented

(1) anterior alveoli labio-lingually oriented, posterior alveoli mesio-distally oriented

(2) all alveoli labio-lingually oriented

4. Premaxillary alveoli, overlap of the first and second alveoli in palatal view (**Ordered**; Hendrickx and Mateus (2014) #4):

(0) absent

(1) present, partial

(2) present, almost complete

5. Premaxillary alveoli, overlap of the second and third alveoli in palatal view (Hendrickx and Mateus (2014) #5):

(0) absent

(1) present

6. Premaxillary alveoli, overlap of the third and fourth alveoli in palatal view (Hendrickx and Mateus (2014) #6):

(0) absent

(1) present

7. Premaxillary teeth (or alveoli), size (Unordered; Modified from Holtz et al. (2004) #261):

(0) all approximately equal in size

(1) posterior teeth (or alveoli) smaller than anterior teeth (or alveoli)

(2) anterior teeth (or alveoli) smaller than posterior teeth (or alveoli)

8. Anterior premaxillary teeth (or alveoli), size (Unordered; Hendrickx and Mateus (2014) #8):

(0) significantly smaller than the first six anterior maxillary teeth (or alveoli)

(1) subequal in size than the first six anterior maxillary teeth (or alveoli)

(2) significantly larger than the first six anterior maxillary teeth (or alveoli)

9. Posterior premaxillary teeth (or alveoli), size (Modified from Holtz (2001) #15):

(0) significantly smaller than the first six anterior maxillary teeth (or alveoli)

(1) subequal in size than the first six anterior maxillary teeth (or alveoli)

(2) significantly larger than the first six anterior maxillary teeth (or alveoli)

10. First premaxillary tooth (or alveolus), size (Unordered; Modified from Sereno et al. (1998) #38):

(0) subequal in size than second tooth (or alveolus)

(1) significantly smaller than second tooth (or alveolus)

(2) significantly bigger than second tooth (or alveolus)

11. Second premaxillary tooth (or alveolus), size (Unordered; Modified from Currie (1995) #4):

(0) subequal in size than third (and fourth) premaxillary tooth (or alveolus)

(1) significantly smaller than third (and fourth) tooth (or alveolus)

(2) significantly larger than third (and fourth) tooth (or alveolus)

12. Posteriormost premaxillary tooth (or alveolus), mesiodistal length in palatal view (Unordered; Hendrickx and Mateus (2014) #12):

(0) subequal in size than more anterior teeth (or alveoli)

(1) significantly smaller than more anterior teeth (or alveoli)

(2) significantly larger than anterior teeth (or alveoli)

13. Distal premaxillary alveoli, shape in palatal view (Hendrickx and Mateus (2014) #13):

(0) oval to subcircular

(1) subrectangular

14. Premaxillary tooth row, posterior extension (position of posteriormost premaxillary tooth): (Modified from Sereno (1999) #36):

(0) aligned (ventral) to external naris

(1) anterior to external naris

15. Premaxilla in palatal view (**Ordered**; Hendrickx and Mateus (2014) #16):

(0) unconstricted

(1) slightly constricted

(2) strongly constricted, terminal rosette of premaxilla

16. Subnarial gap/diastema (i.e., posterior part of premaxillary alveolar margin edentulous, resulting in an interruption of the upper tooth row) (Modified from Gauthier (1986) #36; Sereno (1999) #34; Welles (1984); Rowe 1989; Rowe and Gauthier 1990):

(0) absent

(1) present and short, diastema not extensive enough to host more than one tooth

(2) present and long, diastema extensive enough to host more than one tooth

17. First premaxillary alveoli open (**New**):

(0) ventrally, decumbent teeth

(1) anteroventrally, procumbent teeth

II. MAXILLA ALVEOLI/TEETH

18. Maxillary teeth (**Ordered**; Modified from Holtz (1998b) #56):

(0) present in the anterior and posterior portions of the maxilla (posteriormost portion excluded)

(1) absent in the anteriormost portions of the maxilla

(2) absent in the posterior portion of the maxilla (i.e., more than one fourth of the bone edentulous)

(3) absent in the whole maxilla, toothless maxilla

19. Number of maxillary teeth (or alveoli) (**Ordered**; Modified from Carrano et al. (2002) #58):

(0) >19

(1) 18-19

(2) 16-17

(3) 15

(4) 10-14

(5) 1-9

20. Anterior maxillary teeth (or alveoli), size (Unordered; Modified from Zanno et al. (2009) #340):

(0) subequal in size than posterior teeth (or alveoli)

(1) significantly larger than posterior maxillary teeth (or alveoli)

(2) significantly smaller than posterior maxillary teeth (or alveoli)

21. Mid-maxillary teeth (or alveoli), mesiodistal length (Unordered; Hendrickx and Mateus (2014) #19):

(0) subequal in size than anteriormost maxillary teeth (or alveoli)

(1) significantly larger than anteriormost maxillary teeth (or alveoli)

(2) significantly smaller than anteriormost maxillary teeth (or alveoli)

22. First maxillary tooth (or alveolus), size (Modified from Sereno et al. (1998) #38):

(0) significantly smaller than second tooth (or alveolus)

(1) subequal in size than second tooth (or alveolus)

23. First maxillary teeth (or alveoli) open (Tykoski (2005) #26; Rowe 1989):

(0) ventrally, decumbent teeth

(1) anteroventrally, procumbent teeth

24. Mid-maxillary teeth, inclination (Unordered; Hendrickx and Mateus (2014) #22):

(0) pointing ventrally (decumbent)

(1) pointing lateroventrally (laterocumbent)

(2) pointing anteroventrally (procumbent)

(3) pointing posteroventrally (retrocumbent)

25. Maxillary alveoli, shape in palatal view (Unordered; Hendrickx and Mateus (2014) #23):

(0) oval to lenticular

(1) subrectangular

(2) circular

(3) merged to form an open alveolar groove (interdental septa absent)

26. Maxillary tooth row, posterior extension (position of posteriormost tooth) (**Ordered**; Modified from Gauthier (1986) #38; Harris (1998) #3; Holtz (1998b) #133; Rauhut (2003) #70):

(0) posterior to the anteriormost rim of orbit

(1) anterior or aligned to the anteriormost rim of orbit, posterior to the posteriormost rim of antorbital fenestra

(2) anterior or aligned to the posteriormost rim of antorbital fenestra, posterior to the anteriormost rim of antorbital fenestra

(3) aligned to the anteriormost rim of antorbital fenestra

(4) anterior to the anteriormost rim of the antorbital fenestra

III. DENTARY ALVEOLI/TEETH

27. Dentary teeth (**Ordered**; Modified from Sereno (1999) #175):

(0) present in the anterior and posterior portions of the dentary

(1) absent in the anteriormost portion of the dentary

(2) absent in the posterior portion of the dentary (more than one fourth of the bone edentulous)

(3) absent in the whole dentary, toothless dentary

28. Number of dentary teeth (or alveoli) (**Ordered**; Modified from Norell et al. (2001b) #86; Carrano et al. (2002) #59):

(0) > 25

(1) 18-25

(2) 15-17

(3) < 15

29. Dentary alveoli in dorsal view (Chiappe et al. (1996) #92; Currie 1987):

(0) in separate alveoli

(1) merged to form an open alveolar groove (interdental septa absent)

30. Anteriormost dentary teeth (or alveoli), size (Unordered; Modified from Rauhut (2003) #83; Tykoski (2005) #101):

(0) subequal in size than mid- and posterior dentary teeth (or alveoli)

(1) significantly larger than mid- and posterior dentary teeth (or alveoli)

(2) significantly smaller than mid- and posterior dentary teeth (or alveoli)

31. First dentary tooth (or alveolus), size in comparison to second and third dentary alveoli (Unordered; Modified from Gauthier (1986) #36 and Harris (1998) #48. Based on Holtz et al. (2004) #213 and Sereno et al. (2004) #71):

(0) subequal in size

(1) first tooth (or alveolus) substantially smaller

(2) first tooth (or alveolus) substantially larger

32. Mid-dentary teeth (or alveoli), size (Unordered; Modified from Pérez-Moreno et al. (1994) #3):

(0) subequal in size than anterior maxillary teeth (or alveoli)

(1) significantly smaller than anterior maxillary teeth (or alveoli)

(2) significantly larger than anterior maxillary teeth (or alveoli)

33. Terminal rosette of dentary, number of teeth (or alveoli) (Unordered; Hendrickx and Mateus (2014) #31):

(0) terminal rosette absent

(1) four teeth (or alveoli)

(2) five teeth (or alveoli)

34. First two or three dentary alveoli open (Hendrickx and Mateus (2014) #32):

(0) dorsally, decumbent teeth

(1) anterodorsally, procumbent teeth

35. Mid-dentary teeth, inclination (**New**):

(0) pointing dorsally, decumbent

(1) pointing anterodorsally, procumbent

36. Dentary teeth, spacing (Norell et al. (2001b) #90):

(0) evenly spaced

(1) anterior dentary teeth more closely appressed than those in middle and posterior parts of the tooth row

VI. PALATAL TEETH

37. Palatal teeth on the pterygoid (Sereno (1999) #107):

(0) present

(1) absent

V. MESIAL TEETH

***Crown***

38. Mesial teeth, constriction between root and crown (**Ordered**; Modified from Pérez-Moreno et al. (1994) #4; Martin et al. (1980):

(0) absent

(1) constriction weak, base of crown occupying more than 85% of largest crown width

(2) constriction important, base of crown occupying 85% or less of largest crown width

39. Mesial teeth, constriction between root and crown along the tooth row (Hendrickx and Mateus (2014) #35):

(0) present in some teeth

(1) present in all teeth

40. Mesial teeth, height of the largest crown (CH in centimetres) in subadults/adults (**Ordered**; Modified from Hendrickx and Mateus (2014) #36):

(0) CH ≤ 1

(1) 1 < CH ≤ 6

(2) CH > 6

41. Mesial teeth, labiolingual compression of the crown (CBR = CBW/CBL) (Unordered; Modified from Sereno et al. (1998) #17; Charig and Milner 1997):

(0) CBR < 0.5, lenticular and strongly labiolingually compressed

(1) 0.5<CBR ≤ 0.75, oval to lenticular

(2) weak, 0.75 < CBR < 1.2, tooth subcircular

(3) CBR ≥ 1.2, teeth labiolingually elongated

42. Mesial teeth, baso-apical elongation of the crown (CHR = CH/CBL) (Unordered; Hendrickx and Mateus (2014) #38):

(0) strongly elongated, CHR > 3

(1) important, 2.5 < CHR ≤ 3

(2) normal, 2 < CHR ≤ 2.5

(3) weak, CHR ≤ 2

43. Mesial teeth, crown recurvature (lingually or distally) (Unordered; Modified from Sereno et al. (1998) #35):

(0) present, strongly recurved

(1) present, slightly recurved

(2) absent, tooth crown straight and apex centrally positioned or almost centrally positioned

44. Mesial teeth, distal margin of the crown in lateral view (Unordered; Modified from Canale et al. (2009) #5; Smith 2007):

(0) mainly concave

(1) straight

(2) mainly convex

45. Mesial teeth, outline of basal cross-section of the crown in the mesialmost tooth (Unordered; Modified from Bakker et al. (1988) #1):

(0) subcircular, ovoid or elliptical

(1) lanceolate, with acute and well-developed distal carina

(2) Salinon shape, with labial margin convex and lingual margin biconcave

(3) D-shaped or J-shaped, with lingual margins strongly convex and labial margin convex or sigmoid

(4) U-shaped, with mesial and distal margin subparallel; lenticular, with acute and well-developed distal and mesial carinae

46. Mesial teeth, concave surface adjacent to the carina (Unordered; Hendrickx and Mateus (2014) #42):

(0) absent

(1) on the labial surface and adjacent to the distal carina

(2) on the lingual surface and adjacent to both carinae

(3) on the lingual surface and adjacent to the mesial carina only

(4) on the lingual surface and adjacent to the distal carina only; one main concave surface centrally positioned on the lingual side of the crown

***Carinae***

47. Mesial teeth, mesial carina (Hendrickx and Mateus (2014) #43):

(0) absent

(1) present

48. Mesial teeth, mesial carina (Modified from Senter et al. (2004) #20):

(0) non-serrated

(1) serrated

49. Mesial teeth, distal carina (Hendrickx and Mateus (2014) #45):

(0) serrated

(1) non-serrated

50. Mesial teeth, mesial carina (**Ordered**; Modified from Hendrickx and Mateus (2014a) #46):

(0) straight and centrally positioned on the crown

(1) slightly twisted, curves onto the mesiolingual surface

(2) strongly twisted, curves onto the lingual surface

(3) almost straight and strongly lingually deflected

51. Mesial teeth, mesial carina, and if serrated, mesial serration (Unordered; Modified from Benson (2010) #89):

(0) terminates well-above the cervix

(1) extends to the cervix or just above it

(2) terminates well beneath the cervix

52. Mesial teeth, distal carina (Modified from Hendrickx and Mateus (2014) #48):

(0) centrally positioned or slightly displaced

(1) strongly labially deflected

53. Mesial teeth, position of mesial carina on the crown in articulation in mesialmost teeth (Unordered; Modified from Currie (1995) #2 and Hendrickx and Mateus (2014) #47):

(0) facing mostly labially

(1) facing mostly mesially

(2) facing mostly lingually

54. Mesial teeth, position of distal carina on the crown in articulation in mesialmost teeth (Modified from Hendrickx and Mateus (2014) #48):

(0) facing mostly distally or labiodistally

(1) facing mostly lingually

***Denticles***

55. Mesial teeth, average number of denticles per five millimeters on mesial carina at two-thirds height of crown (MCA) in subadults/adults (Unordered; Modified from Russell and Dong (1993) #20):

(0) ≥ 20

(1) 14-19

(2) 9-13

(3) ≤ 8

56. Mesial teeth, average number of mid-crown denticles per five millimeters on distal carina (DC) in subadults/adults (Unordered; Modified from Russell and Dong (1993) #20):

(0) ≥ 20

(1) 14-19

(2) 9-13

(3) ≤ 8

57. Mesial teeth, denticle size (except in embryos and hatchlings) (**Ordered**; Hendrickx and Mateus (2014) #53):

(0) minute denticles, more than 250 denticles on the distal carina

(1) normal in height, between 15 to 250 denticles on the distal carina

(2) very larges denticles, fewer than 15 denticles on the distal carina

58. Mesial teeth, denticles on mesial carina (Unordered; Modified from Norell et al. (2001b) #88):

(0) rounded and symmetrically convex

(1) rounded and asymmetrically convex

(2) strongly hooked/pointed, denticles with a tip pointing apically

59. Mesial teeth, denticles on distal carina (Unordered; Modified from Senter et al. (2004) #23):

(0) rounded and symmetrically convex

(1) rounded and asymmetrically convex

(2) strongly hooked/pointed, denticles with a tip pointing apically

60. Mesial teeth, size of mesial denticles relative to distal denticles (DSDI) (Unordered; Modified from Holtz (1998b) #129; Rauhut and Werner 1995):

(0) mesial and distal denticles of same size, 0.8 < DSDI <1.2

(1) mesial denticles larger than distal ones, DSDI < 0.8

(2) distal denticles larger than mesial ones, DSDI > 1.2

61. Mesial teeth, denticles contiguous over tip or very close to the apex (Modified from Harris (1998) #45):

(0) present

(1) absent

62. Mesial teeth, interdenticular sulci (Unordered; Modified from Benson (2010) #90):

(0) absent

(1) present, short

(2) present, long and well-developed

***Ornamentations***

63. Mesial teeth, flutes (i.e., subparallel longitudinal grooves separated by acute ridges) on the crown (Unordered; Modified from Sereno et al. (1998) #18; Charig and Milner 1997):

(0) absent

(1) present on the lingual surface only

(2) present on both labial and lingual surfaces

(3) present on the labial surface only

64. Mesial teeth, longitudinal groove on the labial and/or lingual side of the crown (Unordered; Hendrickx and Mateus (2014) #60):

(0) absent

(1) present, a single groove centrally positioned

(2) present, a single groove mesially positioned

65. Mesial teeth, longitudinal ridge (differing from flutes) on the lingual side of the crown (Unordered; Hendrickx and Mateus (2014) #61):

(0) absent

(1) present, a single ridge centrally positioned

66. Mesial teeth, basal striations on both lingual and labial sides of the crown (Hendrickx and Mateus (2014) #62):

(0) absent

(1) present

VI. LATERAL TEETH

***Crown***

67. Lateral teeth, constriction between root and crown (**Ordered**; Pérez-Moreno et al. (1994) #4; Martin et al. 1980):

(0) absent

(1) constriction weak, crown base occupying more than 85% of largest crown width mesiodistally

(2) constriction important, crown base occupying 85% or less of largest crown width mesiodistally

68. Lateral teeth, constriction between root and crown along the tooth row (Hendrickx and Mateus (2014) #64):

(0) present in some teeth

(1) present in all teeth

69. Lateral teeth, height of the largest crown (CH in centimetres) in subadults/adults (**Ordered**; Hendrickx and Mateus (2014) #65):

(0) CH ≤ 1

(1) 1 < CH ≤ 6

(2) CH > 6

70. Lateral teeth, labiolingual compression of the crown (CBR = CBW/CBL) (Unordered; Hendrickx and Mateus (2014) #66):

(0) important, CBR ≤ 0.5, tooth strongly flattened

(1) normal, 0.5 < CBR ≤ 0.75

(2) weak, CBR > 0.75, tooth incrassate or subcircular

71. Lateral teeth, baso-apical elongation of the crown (CHR = CH/CBL) (Unordered; Hendrickx and Mateus (2014) #67):

(0) weak, CHR ≤ 1.5

(1) normal, 1.5 < CHR ≤ 2.5

(2) important, CHR > 2.5

72. Lateral teeth, distal margin of crown in lateral view (Unordered; Modified from Hendrickx and Mateus (2014) #68):

(0) strongly concave

(1) slightly concave, roughly straight, or straight, apex positioned at the same level as distal profile

(2) convex, apex positioned mesial to distal profile

(3) sigmoid, basal half concave and apical half convex

(4) sigmoid, basal half convex and apical half concave

73. Lateral teeth, mesial margin of crown in lateral view (Modified from Hendrickx and Mateus (2014) #69):

(0) strongly convex

(1) slightly convex, almost straight

74. Lateral teeth, mesiodistal curvature of the labial surface of the crown at one third of the crown (Unordered; Modified from Hendrickx and Mateus (2014) #70 and #73; Peyer 2006):

(0) convex

(1) surface centrally positioned on the crown roughly flattened

(2) surface centrally positioned on the crown concave, labial depression restricted to the crown base

(3) surface centrally positioned on the crown concave, labial depression extends along the basal half of the crown or more apically

75. Lateral teeth, concave surface adjacent to carinae all along the crown (Unordered; Hendrickx and Mateus (2014) #71):

(0) absent

(1) present on labial surface and adjacent to distal carina

(2) present on lingual surface and adjacent to distal carina

(3) present on labial surface and adjacent to both mesial and distal carinae

(4) present on lingual surface and adjacent to both mesial and distal carinae

76. Lateral teeth, outline of basal cross-section of the crown (Unordered; Hendrickx and Mateus (2014) #72):

(0) subcircular

(1) lenticular or lanceolate

(2) elliptical or bean-shaped (i.e., longitudinal depression centrally positioned on one side only)

(3) 8-shaped (i.e., longitudinal depression centrally positioned on both lingual and labial margins)

(4) subrectangular

***Carinae***

77. Lateral teeth, mesial carina (Hendrickx and Mateus (2014) #74):

(0) present

(1) absent

78. Lateral teeth, mesial carina (Modified from Senter et al. (2004) #20):

(0) serrated

(1) non-serrated

79. Lateral teeth, distal carina (Hendrickx and Mateus (2014) #77):

(0) present

(1) absent

80. Lateral teeth, distal carina (Hendrickx and Mateus (2014) #78):

(0) serrated

(1) non-serrated

81. Lateral teeth, extension of mesial carina relative to distal carina (Hendrickx and Mateus (2014) #79):

(0) mesial carina extends at the same level or terminates more apically than the distal carina

(1) mesial carina extends more basally than the distal carina

82. Lateral teeth, mesial carina, and if serrated, basalmost serration of the mesial carina (Unordered; Modified from Benson (2010) #89):

(0) terminates around mid-height of crown or more apically

(1) extends to base of crown or slightly above the cervix

(2) terminates well beneath the cervix

83. Lateral teeth, twisted mesial carina in some crowns (Modified from Currie (1995) #2):

(0) absent, mesial carina centrally positioned on mesial margin or weakly curved lingually towards the base in all teeth

(1) present, mesial carina strongly twisting onto the mesiolingual surface in some teeth

84. Lateral teeth, split carina in some teeth (Unordered; **New**):

(0) absent

(1) present in the mesial carina

(2) present in the distal carina

85. Lateral teeth, distal carina, and if serrated, basalmost serration of the distal carina (Unordered; Modified from Benson (2010) #89):

(0) extends to the cervix or just above it

(1) terminates well beneath the cervix

(2) terminates well above the cervix

86. Lateral teeth, profile of the distal carina on the crown in distal view (Hendrickx and Mateus (2014) #82):

(0) straight or very slightly bowed

(1) strongly bowed or sigmoid

87. Lateral teeth, position of distal carina on the crown in distal view (Hendrickx and Mateus (2014) #83):

(0) centrally positioned or slightly displaced, crown subsymmetrical

(1) strongly labially deflected, crown asymmetrical

***Denticles***

88. Lateral teeth, average number of denticles per five millimeters on mesial carina at two-thirds height of crown (MCA) in subadults/adults (Unordered; Modified from Russell and Dong (1993) #20):

(0) ≥ 30

(1) 16-29

(2) 9-15

(3) ≤ 8

89. Lateral teeth, average number of mid-crown denticles per five millimeters on distal carina (DC) in subadults/adults (Unordered; Modified from Russell and Dong (1993) #20):

(0) ≥ 30

(1) 16-29

(2) 9-15

(3) ≤ 8

90. Lateral teeth, denticle number on both mesial and distal carinae (except in embryos and hatchlings) (**Ordered**; Hendrickx and Mateus (2014) #86):

(0) more than 250 denticles (minute denticles or very large number of denticles of normal size)

(1) between 15 to 250 denticles (denticles of average size)

(2) fewer than 15 denticles (very large denticles or very small number of small denticles)

91. Lateral teeth, shape of denticles on mesial carina in lateral view (Unordered; Modified from Norell et al. (2001b) #88):

(0) symmetrically convex

(1) asymmetrically convex

(2) hooked/pointed

92. Lateral teeth, shape of denticles on distal carina in lateral view (Unordered; Senter et al. (2004) #23):

(0) symmetrically convex

(1) asymmetrically convex

(2) hooked/pointed

93. Lateral teeth, shape of mesial margin of rounded denticles on mesial carina in lateral view (Hendrickx and Mateus (2014) #89):

(0) parabolic

(1) subrectangular, with flattened surface

94. Lateral teeth, shape of distal margin of rounded denticles on distal carina in lateral view (Unordered; Hendrickx and Mateus (2014) #90):

(0) parabolic

(1) subrectangular, with flattened surface

(2) semi-circular

95. Lateral teeth, shape of denticles at two-thirds height of crown (MC-MA) on mesial carina in lateral view (Unordered; Hendrickx and Mateus (2014) #91):

(0) longer apicobasally than mesiodistally, vertical subrectangular

(1) as long mediodistally as apicobasally, subquadrangular

(2) longer mediodistally than apicobasally, horizontal subrectangular

96. Lateral teeth, shape of mid-crown denticles (DC) on distal carina in lateral view (Unordered; Hendrickx and Mateus (2014) #92):

(0) as long mediodistally as apicobasally, subquadrangular

(1) longer mediodistally than apicobasally, horizontal subrectangular

(2) longer apicobasally than mesiodistally, vertical subrectangular

97. Lateral teeth, denticle size along the carinae (Hendrickx and Mateus (2014) #93; Mateus et al. 2011):

(0) regular, gradual change in denticle size

(1) irregular, sporadic change in denticle size

98. Lateral teeth, biconvex apical denticles (i.e., biconvex external margin of denticle) on mesial carina in lateral view (Hendrickx and Mateus (2014) #94):

(0) absent

(1) present

99. Lateral teeth, orientation of mesiodistal axis of apical denticles on mesial carina in lateral view (Hendrickx and Mateus (2014) #95):

(0) perpendicular to mesial margin

(1) inclined apically from mesial margin

100. Lateral teeth, orientation of mesiodistal axis of mid-crown denticles on distal carina in lateral view (Hendrickx and Mateus (2014) #96):

(0) perpendicular to distal margin

(1) inclined apically from distal margin

101. Lateral teeth, average number of denticles on mesial carina (Unordered; Hendrickx and Mateus (2014) #97):

(0) higher number of denticles basally than at the mid-crown

(1) lower number of denticles basally than at the mid-crown

(2) subequal number of denticles basally than at the mid-crown

102. Lateral teeth, average number of denticles on mesial carina (Unordered; Hendrickx and Mateus (2014) #98):

(0) higher number of denticles apically than at the mid-crown

(1) lower number of denticles apically than at the mid-crown

(2) subequal number of denticles apically than at the mid-crown

103. Lateral teeth, average number of denticles on distal carina (except in embryos and hatchlings) (Hendrickx and Mateus (2014) #99):

(0) higher number of denticles basally than at the mid-crown

(1) subequal or lower number of denticles basally than at the mid-crown

104. Lateral teeth, average number of denticles on distal carina (Unordered; Hendrickx and Mateus (2014) #100):

(0) higher number of denticles apically than at the mid-crown

(1) lower number of denticles apically than at the mid-crown

(2) subequal number of denticles apically than at the mid-crown

105. Lateral teeth, size of mesial denticles relative to distal denticles (DSDI) (Unordered; Rauhut and Werner 1995):

(0) mesial and distal denticles of same size, 0.8 < DSDI <1.2

(1) mesial denticles larger than distal ones, DSDI < 0.8

(2) distal denticles larger than mesial ones, DSDI > 1.2

106. Lateral teeth, distal denticles on the apex (Harris (1998) #45):

(0) contiguous over tip, or very close to the apex

(1) distal denticles disappear well beneath apex

107. Lateral teeth, interdenticular space between mid-crown denticles on the distal carina (Hendrickx and Mateus (2014) #103):

(0) narrow, less than one third of the denticle width

(1) broad, more than one third of the denticle width

108. Lateral teeth, interdenticular sulci between apical denticles on the mesial carina (Unordered; Modified from Benson (2010) #90):

(0) absent

(1) present, short and poorly developed, shorter than proximodistal denticle height

(2) present, long and well-developed, equal or longer than proximodistal denticle height

109. Lateral teeth, interdenticular sulci between mid-crown denticles on the distal carina (Unordered; Modified from Benson (2010) #90):

(0) absent

(1) present, short and poorly developed, shorter than proximodistal denticle height

(2) present, long and well-developed, equal or longer than proximodistal denticle height

110. Lateral teeth, interdenticular sulci between basalmost denticles on the distal carina (Unordered; Modified from Benson (2010) #90):

(0) absent

(1) present, short and poorly developed, shorter than proximodistal denticle height

(2) present, long and well-developed, equal or longer than proximodistal denticle height

***Ornamentations***

111. Lateral teeth, flutes (i.e., subparallel longitudinal grooves separated by acute ridges) on the crown (Unordered; Modified from Sereno et al. (1998) #18; Bakker et al. (1988) #2):

(0) absent

(1) present on the lingual surface

(2) present on labial surface or both labial and lingual surfaces

112. Lateral teeth, average number of flutes on the crown (Unordered; Modified from Hendrickx and Mateus (2014) #108):

(0) 1-7

(1) 7-8

(2) >8

113. Lateral teeth, large transversal undulations on the crown in some teeth (Unordered; Modified from Holtz (1998b) #131):

(0) absent

(1) present, tenuous and barely visible with light

(2) present, pronounced and well visible with light

114. Lateral teeth, large transversal undulations on the crown in some teeth when present (Hendrickx and Mateus (2014) #110):

(0) just a few

(1) numerous and closely packed

115. Lateral teeth, marginal undulations (i.e., short undulations adjacent to carinae) in some teeth (Unordered; Modified from Currie and Carpenter (2000) #42; Brusatte et al. 2007):

(0) absent

(1) present and short, the mesiodistal elongation is less than four times the space separating each undulation

(2) present and elongated, the mesiodistal elongation is longer than four times the space separating each undulation

116. Lateral teeth, marginal undulations in some teeth (Hendrickx and Mateus (2014) #112):

(0) present and shallow, only visible with light

(1) present and pronounced, well visible in lateral view

117. Lateral teeth, marginal undulations in some teeth (Unordered; Hendrickx and Mateus (2014) #113):

(0) present only on the mesial side of the crown

(1) present only on the distal side of the crown

(2) present on both mesial and distal sides

118. Lateral teeth, marginal undulations in some teeth (Hendrickx and Mateus (2014) #114):

(0) present and mesio-distally oriented

(1) present and diagonally oriented

119. Lateral teeth, longitudinal groove on the labial and/or lingual surface of the crown (Unordered; Hendrickx and Mateus (2014) #115):

(0) absent

(1) present, a single groove centrally positioned

(2) present, a single groove adjacent to mesial carina

(3) present, two grooves or more

120. Lateral teeth, elongated longitudinal and rounded ridge (differing from flutes) on the lingual surface of the crown (Unordered; Hendrickx and Mateus (2014) #116):

(0) absent

(1) present, a single ridge centrally positioned

(2) present, two or three ridges

(3) present, several fainted ridges

VII. ENAMEL MICROSTRUCTURE

121. Enamel surface texture (Unordered; Hendrickx and Mateus (2014) #117):

(0) smooth or irregular (non-oriented) texture

(1) braided (oriented) texture not clearly visible with light

(2) braided (oriented) texture clearly visible with or without light

(3) deeply veined/anastomosed (oriented) texture

122. Coarse enamel surface texture (Hendrickx and Mateus (2014) #118):

(0) remains baso-apically/diagonally oriented or slightly curved basally close to the carinae

(1) strongly curved basally close to the carinae

123. Enamel microstructure, enamel tubules (Unordered; Hwang (2007) #12):

(0) absent or rare

(1) common only in basal unit layer (BUL) and/or inner potion of enamel

(2) common and extend throughout entire enamel thickness

(3) extremely common and forming an integral structural component of enamel

124. Enamel microstructure, predominant enamel type (Unordered; Modified from Hwang (2007) #13; character state 2 removed):

(0) parallel crystallites

(1) basal unit layer (BUL)

(2) columnar

125. Enamel microstructure, predominant enamel type, percentage of enamel thickness (Hwang (2007) #14):

(0) ≥ 75%

(1) < 75%

126. Enamel microstructure, number of enamel types present in schmelzmuster (Hwang (2007) #15):

(0) one

(1) two

(2) three

(3) four

127. Enamel microstructure, number of different module types present in schmelzmuster (Hwang (2007) #16):

(0) one

(1) two

128. Enamel microstructure, boundary between first and second enamel types from the enamel-dentine junction (EDJ; Hwang (2007) #17):

(0) parallel to EDJ

(1) jagged, varies in distance from EDJ

129. Enamel microstructure, boundary between second and third enamel types from the enamel-dentine junction (EDJ; Hwang (2007) #18):

(0) parallel to EDJ

(1) jagged, varies in distance from EDJ

130. Enamel microstructure, basal unit layer (BUL; Hwang (2007) #19):

(0) present

(1) absent

131. Enamel microstructure, basal unit layer (BUL; Hwang (2007) #20):

(0) poorly developed

(1) well-developed, with distinct planes of separation between adjacent units

132. Enamel microstructure, basal unit layer (BUL), maximum unit diameter (Hwang (2007) #21):

(0) < 10 µm

(1) ≥ 10 µm

133. Enamel microstructure, basal unit layer (BUL; Unordered; Hwang (2007) #22):

(0) < 25% of total enamel thickness

(1) 25-50% of total enamel thickness

(2) ≥ 50% of enamel thickness

134. Enamel microstructure, incremental lines (Unordered; Hwang (2007) #23):

(0) absent

(1) faint, poorly defined

(2) well-defined

135. Enamel microstructure, incremental lines (Unordered; Hwang (2007) #24):

(0) present in one section of the schmelzmuster only

(1) present in more than one section of the schmelzmuster but not throughout entire schmelzmuster

(2) present throughout entire schmelzmuster

136. Enamel microstructure, columnar units closest to the enamel-dentine junction (EDJ), shape of units in cross-sections (Unordered; Hwang (2007) #27):

(0) polygons with sharp corners and more than 4 sides

(1) subcircular or polygons with rounded corners and more than 4 sides

(2) triangles and/or rectangles with sharp corners

137. Enamel microstructure, columnar units closest to the enamel-dentine junction (EDJ; Hwang (2007) #28):

(0) extend straight and unbroken to the outer enamel surface (OES) or to within 20µm below the OES

(1) end, split, or are interrupted less than two-thirds of the distance from the EDJ to OES

138. Enamel microstructure, columnar units closest to the enamel-dentine junction (EDJ), maximum unit diameter (Hwang (2007) #29):

(0) < 15µm

(1) ≥ 15µm

139. Enamel microstructure, columnar units closest to the outer enamel surface (OES; Hwang (2007) #33):

(0) no dominant direction of orientation, planes of separations equally well-developed in all directions

(1) distinct longitudinal orientation, planes of separation better developed in an apicobasal (longitudinal) direction

140. Enamel microstructure, ratio of thickest enamel type in schmelzmuster divided by second thickest enamel type (Unordered; Hwang (2007) #39):

(0) > 7

(1) 1.3 to 7

(2) 1 to 1.3

VIII. ROOT

141. Root, shape in lateral view (Hendrickx and Mateus (2014) #137):

(0) with subparallel mesial and distal margins

(1) with convex margins, root significantly larger than crown base

142. Root, distal shape in lateral view (Sereno et al. (1998) #21; Charig and Milner 1997):

(0) broad

(1) strongly tapered

143. Root, outline of mid-root in cross section (Unordered; Hendrickx and Mateus (2014) #139):

(0) oval to subcircular

(1) 8-shape (i.e., longitudinal depression centrally positioned on both lingual and labial margins)

(2) bean-shaped (i.e., longitudinal depression centrally positioned on one side only)

144. Root, form of the resorption pit in lingual view (Hendrickx and Mateus (2014) #140):

(0) deep and well-delimited depression

(1) shallow concavity

145. Root, transversal undulations below the cervix in some crowns (Hendrickx and Mateus (2014) #141):

(0) absent

(1) present

146. Root, apicobasal length in lateral view (**New**):

(0) less than twice the apicobasal length of the crown

(1) twice or more the apicobasal length of the crown

## Phylogenetic Character Datasets

### Files

The Excel, Mesquite and TNT files are can be obtained by request to the corresponding author.

### Dentition-based data matrix

xread

146 114

Herrerasaurus 01000000000000000011000000020001000010-11[2 3]10000-0--1-0-01-0-?000010-1[0 1 2][0 1][0 1]000[0 2]00000?00000111000[0 1]100000-???0?10000-200---000-????????????????????????

Daemonosaurus 00?000110021?00110412000?00?01010100?0-1?[2 3]000011000010??110?0000000-1?[1 2]0000?00000000000??11000010?000000?0?0000-0-0---000-??????????????????00????

Eoraptor 010100000000?00100110000?00?????????0100[1 2][1 2]100[0 1][0 1]10001101012[1 2]1100000[1 2]00[0 1][0 1]100[1 3]?00000000?001[0 1]1[1 2][1 2]00000001--001000000-0-0---000-????????????????????????

Eodromaeus 01???????????000?0410?000?030??100000?????????????????????????????0-0[0 1]200011[0 1]0000100000001[0 1]000[0 1][0 1]000020000?00000-100---0010????????????????????????

Coelophysis 010?000000000011000101100000010??00010-0[0 1][0 1 2]1000[0 1]1000110-01-0-0030000-1[0 1][0 1]000[1 3]100000[0 1]000??00100[0 1]0[0 1]00000-000000000[0 3]00-0---001000011--1---0------??????

Liliensternus ?????????????????0?????00?010010100010-01?1[0 1]0011000010001?00?000000-1[0 1]1[0 1]000100000000000[0 1][0 1]100[0 1]0[0 1][0 1]0?00-0000010000-0-0---000-??????????????????001?0?

Dracovenator 0111100??10100200??????00???0????????0-1021010110000???21?020000000-10?000010000010010012100000100000?0?2?10000-100---0010??????????????????????0?

Dilophosaurus 011100000101002200411110010201111000?0-11[0 1]0000110001??1[1 2]10020000000-11100[1 2][3 4]1000001000001210000[0 1]100000?0?20?0000-0-0---0010??????????????????001000

Ceratosaurus 00111-000000000000[3 4]11100000[2 3]0011000010-1[1 2]3[0 1][0 1]0011000010[2 3][2 3]10020[0 1]10000-2[0 1]1[0 1 2]0[1 2][0 3 4]10000010000[0 1]22100[0 1]01[0 1 2]001[0 1]0[1 2]0[0 2]001[0 1][0 1 2][0 1 2]0-[1 2]01[0 1][0 1][0 1]001022021000110202000000?000

Genyodectes 011111000000000000??11000?0300110000?0-2[0 1][0 1 2]000011000000221000?000000-2[0 1]1[0 1]0[1 2][2 3 4]2000001000[0 1]12210[0 1]0010001[0 1]-2020010[0 1]00-210---0010????????????????????????

Berberosaurus ??????????????????????????????????????????????????????????????????0-10100[1 2]2300000100?01221[0 1][0 1]???1001?-1020?10000-200---0010????????????????????????

'Limusaurus_juvenile' [1 2]0------[1 2]---0000-[0 1]50100002[1 2]30001000010-0??[0 1]???0-1-------------00000-0??[0 1]0???1-1-------------------------------0-0-0---00????????????????????0[0 1]???0

'Limusaurus_adult' 3----------------3--------3---------1-------------------------------------------------------------------------------------------------------------

Noasaurus ?????????????????04101000?????????????????????????????????????????0-01[0 1]100010000010000001100000000000?1?2?00000-0-0---000-????????????????????????

Masiakasaurus 0?11???1?0???????0?10?000?0300000100?210[2 3][0 1]10231[0 1]02112000100[0 2]0010000-1[0 1][1 2]0000100000100001[0 1][1 2]10[0 1 2][0 1]000000[0 1]000020000010[1 2]00---0010??????????????????001100

Kryptops ?????????????????0??0?001?????????????????????????????????????????0-1?1100010000010000?121[0 1]2[0 1]0000001?0?000022?0-0-0---000-??????????????????0?????

Rugops 0100000?1?00100000?111001???????????????1???2?11?0?010????????????0-1[0 1]?[1 2]000100000100000[1 2]210200000001?0?000000?0-0-0---000-????????????????????????

Abelisaurus 01?000???????00000????00?????????????0-113[1 2][1 2]231100[1 2]0??2210[1 2]00200000-1[0 1][0 1]1000100000100?00221[0 1][1 2]0010011100000000100-101010000-????????????????????????

Arcovenator ??????????????????????????????????????????????????????????????????0-1[0 1]10000100000100101[1 2]21[0 1][0 1]0[0 2][0 1]1000100012000000-0-0---000-??????????????????0?????

Chenanisaurus ????????????????????????????021?0????1?1[1 2]2[0 1][1 2]2311002010331[0 1][0 1]00200001?1[0 1]110??100000200110221[0 1][0 1]00110?01000000012?0-?00---000-??????????????????00???0

Indosuchus 0100000110001000004001001?0?0?100000?0-1[1 2]3[1 2][1 2][2 3][2 3]110[0 1][1 2]010[2 3][2 3]1??0?000000-1[0 1]?[1 2]0001000001000?0221??????????0???0??0110-0-0---000-????????????????????????

Majungasaurus 010000011[0 1]001000002111001102021000001[0 1]01[1 2]3[1 2][1 2][2 3][2 3]110[0 1]2010[2 3][2 3]1[1 2]200200000-1[0 1][0 1][1 2]000100000100000[2 3]21[1 2]2000[0 1]000100000001220-201010000-22021000110102000000?1??

Aucasaurus 01?000??1?00?000004101001?????????????????????????????????????????0-1[0 1][0 1]1000100000100000221[0 1][1 2][0 1]01[0 1]011100?00000000-200---000-??????????????????0?0?1?

Skorpiovenator 01???????????00000110?00?10??????????0-11222?3110020??211???0000000-10[0 1][1 2]000100000100010221000011001[0 1]0?0?0?00000-0-2020000-??????????????????00?1?0

Chilesaurus 01???????????0?01?????02??0????00110?210?122?00-1-------------0000110?12000?1-[0 1][0 1]----200?0??0?0?2?0?0??--?00?0-0-0-0---00????????????????????10??00

Piatnitzkysaurus ?????????????????01?00000?0?0???0000??????????????????????????????0-11[1 2]0010100000[0 1]10[0 1]10[1 2][1 2]10[0 1 2]0[0 2][0 1]1000020002000[1 2][1 2]0-[1 2]1201000[0 1]0??????????????????001100

Marshosaurus 0111100000000000002101000?0100110000?0-1121[0 1]10110000100110020100000-1[0 1][0 1]0000100000000?0[0 1]1[1 2]001[0 1]00[0 1]000000002010100-[1 2][1 2]0---0010??????????????????00?1?0

Monolophosaurus 01??000?0?00?00000410?00?2010001000010-11[2 3][0 1]0101?000110?11?0??000000-1[0 1][0 1][0 1]000100000000[0 1]0[0 1][1 2][1 2]100[0 1][0 2]0[0 1]0000?0?00010000-1010110011??????????????????0??1??

Sciuruminus 01?100000000?0?000?11?03?20300110100?0-0?[1 2][0 1]0??0-[0 1]--?-0-0?-?-100000100?100???1-00---00??-??-[1 2]-0-[0 2]0--0--00-[0 1]1-000-0-0---000-??????????????????1???0?

Eustreptospondylus 011110?0000?000000????000?0300111000???1??10?01100?00122100?0100000-11?0000100000000???[2 3]2100[0 1]0000000-??00000??0-0-0---0010????????????????????????

Afrovenator ?????????????????041010002????????????????????????????????????????0-2010001100000000110321[0 1][0 1][0 1]2010?00-0011001110-0-10000010??????????????????0?1?0?

Dubreuillosaurus 01111000000000000041010002030011000010-11210101100000[0 1]221000?000000-1[0 1][0 1]0000100000000100111[0 1][0 1][0 1]0000000-0010000110-0-0---0010??????????????????000?00

Duriavenator 0?11???0?0??00??00?101000?0?00100000?0-?12??1?110??0?1[2 3]310000?00000-1[0 1]100001000000001??[2 3]210[0 1][0 1]00101[0 1]0-101[0 1]000110-[0 1]00---0010??????????????????00?100

Megalosaurus 0????????????????04101000?03001000001?????????????????????????????0-2[0 1]100001000000001[0 1]0[2 3][2 3]1[0 1][0 1][0 1][0 2]010100-100[0 1]001[1 2][1 2]0-2110[1 2][0 1]0020????????????????????????

Torvosaurus 00111-00000000000041010002030??100001??21???1011000000331???0?00000-2[0 1][1 2]0000100000[0 1]001[0 1]0[2 3][2 3]1[0 1][0 1][0 1]0110?00-101[0 1]0[0 1]1220-2[0 1]10100020??????????????????002100

Baryonyx 0[3 4]1110110011012000?100102?000111100010-12[1 2]1000110020??0000000010000-121[0 1]000000000[1 2]001[0 1]000[0 1]00[0 1][0 1]2111000202[0 2]00000[1 2]00-10100031??????????????????010111

Suchomimus 041110110011012000010010220001111?0010-???1000110020??00000000[1 2]0000-1[1 2][1 2][0 1]000000000[1 2]001[0 1]0[0 1][0 1][0 1]00[0 1][0 1]1010002202[0 2]00000[1 2]00-10[1 2][0 1]0031????????????????????????

'Irritator_Angaturama' 0410001??021012100?????1220?0???????1?????????????????????????????0-12[1 2]2100001010100100-----------------------210-101[0 1]000-????????????????????????

Spinosaurus 0312[0 1]011012101220041001123020111[1 2]10010-?[1 2]??1001010[1 2]001--------20000-2[1 2][1 2][1 2]100001010[0 1]00100-----------------------220-0---0031??????????????????0101?[0 1]

Erectopus ??????????????????????000?????????????????????????????????????????0-10[1 2]001010000010000022100[0 1]011010000000000100-1[0 1]0---000-????????????????????????

Yangchuanosaurus 011110200001?00000[3 4]00[0 1]00?20[2 3]01??000010-1?[1 2]11??110[1 2]?0?0?21?0???????0-2?2[0 1]0??20000???????22?????????????????????????????????????????????????????????

Sinraptor_dongi 01111[0 1]000000000000[3 4]01000020200?1000010-1[1 2 3]?11[2 3][1 3]1101[1 2]0102210[0 1]00[1 2]02000-2[0 1][0 1][0 1]0[1 2][0 1 2][2 3]00000[1 2]00[0 1][0 1][0 1]221[0 1][0 1]00[1 2]1000000000011[1 2][0 1]0-211010000-??????????????????001[0 1]01

Sinraptor_hepingensis 01?11[0 1]000000?00000401000?2020101000010-1?1[1 2][1 2]??110???10????????????0-201[0 1]0[1 2]0?00000[1 2]0?1?12210[0 1]00110?00?000001???0-????????0-??????????????????00?0?1

Allosaurus 021110011000000000[2 3]00100020201[0 1]1000010-1[2 3][0 1 2][0 1][0 1][2 3][2 3]110[1 2]10102[2 3]1[0 1][0 1]00[0 1 2]0[0 2][0 1]00-2[0 1 2][1 2][0 1]000[0 1 2]00000[1 2]11[0 1][0 1]1[1 2][1 2]1[0 1][0 1][0 1]011000[0 1]00000011[1 2][1 2]0-2[0 1]2[0 1]1[0 1]00[0 1]02202110011111200000[0 1][0 1][0 1]1[0 1]

Neovenator 0211112011200000003100000?0?001100001?????????????????????????????0-111001[1 2]20000000011[0 1]2[1 2]11100110010-0000011110-2[0 1]20100020??????????????????10211[0 1]

Fukuiraptor ??????????????????????000?0?0?1??000?0-1[1 2]?0030110210??[0 1]110[0 1]0??00000-1[0 1][0 1 2]00??100000[0 1]00010[1 2][1 2]1[0 1][0 1]000[0 1]0?0000000002220-[1 2]020?000??????????????????????????

Australovenator ??????????????????????????01001?0000?0-1[1 2][2 3]00?3110210??1[1 2]1?????00000-1[0 1]100[2 3][1 2]?000000000?[0 1]221?[0 1]?00[0 1]0??0-000001???0-??????0020????????????????????????

Megaraptor 0?[1 2]11??0?0??00??0030100002????????????????10?3100200??-11-0-00000?0-1[0 1][0 1]002231-00---000[0 1]-11-0-0-100-0--00-01-000-100---0010??????????????????100100

Orkoraptor ??????????????????????????????????????????????????????????????????0-1??003231-00---0[0 1][0 1][0 1]-21-0-0-100-0--0?-?1-110-??0---0[0 2]10??????????????????0?1???

Acrocanthosaurus 01111100000000000031010002020011000010-2[1 2][1 2]100011000[0 1]00[1 2]210020000000-2[0 1][0 1 2]0000?00000[0 1]0011[0 1][1 2][1 2][0 1]001[0 1]110111010[1 2][0 1]010[0 1]00-2[0 1][1 2][0 1]100020????????????????????????

Eocarcharia ?????????????????0310?000?????????????????????????????????????????0-101[0 3]00020000000011[0 1]211[0 1][0 1]0[0 2][1 2]10110-1011001110-100---0010????????????????????????

Carcharodontosaurus ?????????????????0400100020?0?1?0000??????????????????????????????102[0 1]1[1 2 3]000100001200110[2 3][2 3]1110010011[0 1]00001001[0 1][0 1]0-[0 1]021200010??????????????????001100

Giganotosaurus 01111101111?000000??0?000?02001?000010-02?110011001000221?00?10000102[0 1][1 2][0 3]000100000[1 2]00110[2 3][2 3]1[0 1][0 1]001[0 1]00000000101[1 2]220-[1 2]0[1 2]0200010??????????????????00[1 2]100

Mapusaurus ?????????????????04101000?0?0???0000?0-12[2 3]10001101?1??221??0000000102[0 1][0 1][0 1 3]000100000[1 2]00110[2 3][2 3]1[0 1][0 1]001100000000[0 1]01[1 2]220-0-[1 2][0 1]200010??????????????????[0 1]0[1 2]100

Bicentenaria ???000???00?0???0??????0?????????????0-?[1 2]???00??0??0?0?0??0??000000?0?100?0100000100000001000001000000002000000-0-0---0020??????????????????0000??

Zuolong 01?0002001002000?040?1000????????????0?1?[2 3]2140110301??0010020000000??[0 1]?[1 2]0?0?00000000[0 1]??1[0 1]10000?10?01-?0???1?000-1?0---0010??????????????????00[1 2]?10

Proceratosaurus 011111000000000000011100?201021101001100?[2 3]103[0 3]1102011[1 2]0010020000010-1[0 1][0 1]00[1 2]0[1 3]00000[0 1]100[0 1]10[0 1]10000000000-0002000[0 1][0 1]0-0-0---0010????????????????????????

Guanlong 011111200?1??00000311100?2??????????10-1[1 2 3]3[1 2]13[0 3]11021121[1 2]11[0 1][0 1]00000000-1[0 1][0 1]00001[0 1]0000[0 1][0 1]00011110[0 1]1[0 2]010000-0?02010[0 1]?0-100---0010????????????????????????

Dilong 011110000002?00000410000??030[0 2]11010010-0[2 3][0 1 2 3][1 2][0 1]4[0 3][0 1]11[1 3]012000100[0 2]0000100-1[0 1 2][0 1 2][0 1]0[0 2]0?000000000011110[0 1][0 1][0 2]0[0 1 2]0000-0002010[0 1]0[0 1]2100---00[1 2]0??????????????????11[1 2]10[0 1]

Eotyrannus 01211100000000000???10001?0?02110000?0-1311[0 1]43110311??[1 2][1 2]10000100000-111[0 1]0001000000001001[1 2]10000000000-0002000110-0-0---0010????????????????????1???

Raptorex 01211100000000000040010002020211000010-03[0 1]104211031121??10020000100-?[0 1][0 1]00[0 1]0100000[0 1]10[0 1][0 1]1??100[0 1][0 2]1[0 1]000000000000100-100---0010??????????????????0?1000

Gorgosaurus 012211000000000000[3 4]0110002020011000010-1[2 3][0 1 2 3]1142110[2 3]1[0 1][1 2][0 1]1[1 2]1?000000[0 1]00-1[0 1 2][1 2][0 1]000[0 1]00000[0 1]10[0 1]01[1 2][1 2]100[0 1]01[0 1]00000[0 2]0[0 2]00111[0 1]0-1[0 1]0---0010220310101001011112001000

Alioramus ?????????????????02111000201021100001?????????????????????????????0-?[0 1][0 1]00[1 2]0[1 3]00000[0 1]10[0 1]01??100001[0 1]000[0 1]00000000100-210---0010??????????????????001000

Daspletosaurus 012211000000000000[2 3 4][0 1]100002020011000010-13[0 1]11421[0 1][0 1][2 3][0 1][0 1]2[0 1]221[0 1]0[0 2]0100100-2[0 1 2][0 1 2][0 1]000[0 1]00000111[0 1]0[0 1][2 3]2111[0 1]01[0 1]00000[0 2]0[0 2]0011[1 2]10-[1 2]11000001022021101---1011112[0 1][0 1][0 1][0 1]1[0 1]

Tyrannosaurus 01221100000000000041110002030211000010-233[0 1]04[0 2]11031[0 1][1 2][0 1][2 3][2 3]10000000[0 1]00-2[1 2][0 1 2][0 1]000000000[0 1]11001[2 3][2 3]100001[0 1]0100020200122?0-2[0 1]112[0 1]00[0 1]022021101---0-01112001[0 1]0[0 1]

Compsognathus 01?100?000?1?0??00210?00?20100?1000010-0?3[0 1 2]0?00-1-------------00000-0[0 1][0 1]0000?1-0[0 1]---02??-01-0-[0 1]-20--0--00-10-000-0-0---000-??????????????????10?000

Juravenator 01??00?00?01?0?000510000?2?3?????????0-0?[0 1 2]00??0-[0 1]--?-0-0--0-1000000-0?[1 2]00?011-01---02??-01-0-0-[0 2]0--?--[0 1]0-[0 1]0-000-0-0---000-??????????????????10??00

Scipionyx 02?100-11122?00100510000?2030011000010-??2000?0-1-------------10000-??[0 1 2][0 1]02011-00---0[0 2]??-?--0-0-[0 2]0--0--?0-10-000-0-0---000-????????????????????????

Ornitholestes 01110012[0 2]021?000104101000203000101001100[1 2 3]21[0 1][3 4][0 3][0 1]0111010--------00000-0[1 2][0 1]0[0 1]001[0 1]10[0 1]?100201-01-0-0-00--0--?0-1?-0-0-[0 1]00---0010??????????????????0?[0 1]??1

Nqwebasaurus ?????????????????2????0234????????????????????????????????????????0-022210001-1-------------------------------0-0-0---000-????????????????????????

Pelecanimimus 04??????????0000020???00340011?100001110??1[0 1]3?0-1-------------0000[1 2]10?0[0 1][0 1]00?1-1-------------------------------0-0-0---000-??????????????????0?0?0?

Shenzhousaurus 3----------------3--------230-?-011010-02?22000-1-------------00000-021210001-1-------------------------------0-0-0---000-????????????????????????

Garudimimus 3----------------3--------3---------1-------------------------------------------------------------------------------------------------------------

Struthiomimus 3----------------3--------3---------1-------------------------------------------------------------------------------------------------------------

Aorun 011100011000?00002410?00?3000??10?0010-0?[0 2]00[0 1]?0-1-------------00000-0[0 1]?000?11-[0 1]1---0???-01-0-0-[0 2]0--0--?--10-0?0-0-0---0020????????????????????????

Haplocheirus 01??00000000000000012?00[0 2]2000?1?00001000?210000-1--0-0--------00000-0[1 2][1 2]0000?[0 1]00000000??0[0 1]1000000000[0 1]-??0?[0 1]10000-0-0---030-????????????????????????

Shuvuuia ?????????????????2000100340010000000?1102?22000-1-------------00?011021210001-1-------------------------------0-0-0---000-??????????????????0?0???

Mononykus ??????????????????????????????????????????????????????????????????1?021210001-1-------------------------------0-0-0---030-??????????????????0?0?0?

Eshanosaurus ??????????????????????????0001??0?00??????????????????????????????21021[1 2]000000000000200112[0 1][0 1 2]00[0 2][0 2]0000-[1 2]-20010000-0-0---0[0 1]0-??????????????????1?0?0?

Falcarius ?????????????????0?100002?000???000011002[2 3]220[0 5]001-------------0000210[1 2][0 1][2 4]00[0 2 3]0000000102000010000[0 1][0 2]0000-1-1[0 2]00[0 2][0 2]00-100---0[0 1]0-000[0 1]?0-[0 1]00022----0[0 1][0 1]010[0 1]

Jianchangosaurus 3----------------0000?00?2?0000-0000?211221004110201201?2220000?0021021[2 4]00[0 3]0000000002002222200000011-2-20010000-0-0---000-??????????????????110?01

Segnosaurus ??????????????????????????110???0?00??????????????????????????????2112[1 2][1 2][0 1][0 2][0 4]0000000[0 1]22[0 2]0222[0 1 2][0 1 2]00[0 1]2[0 1]0[0 1][0 1]-0-00[0 1]10000-100---[0 3][0 1]1-??????????????????0?????

Erlikosaurus 3----------------10???00?210010?010011002222000-0--0-0-02-1-?00000210212000000000000200??2[0 2][0 2]00020011-2-20010000-0-0---000-??????????????????110??1

Incisivosaurus 0100001222010000005101000213[0 1]00?01101001?22[1 2]001010?010--------00001?0?021000[0 1]?[0 1][0 1]??00200-?01?0?0?10??0??-???0?0--0-0---00[0 3]0????????????????????????

Caudipteryx 11?????--????10013--------3---------10-0?0[0 2][0 1]?-0-1-------------0000------------------------------------------------------0-??????????????????10[1 2]00[0 1]

Chirostenotes 3----------------3--------3---------1-------------------------------------------------------------------------------------------------------------

Citipati 3----------------3--------3---------1-------------------------------------------------------------------------------------------------------------

Halszkaraptor 041111000000000000001?0001010???0???11?0??003-0-1-------------00?00-0??000011-1-------------------------------0-??????00????????????????????0[0 1]?0?0

Buitreraptor ?????????????????0?????0020?0??00000??????????????????????????????0-0[0 1][0 1]003[0 1]31-1-------------------------------0-0-0---[1 3][1 2]0-??????????????????1?1?00

Microraptor 011000100[0 1]20?00000401?00?20?0?110[0 1]0010-0?[1 2][0 1]0?[0 3]0-1--0-0--------00[0 1 2]0100?1[0 1]03131-0[0 1]---0[0 2]??-0[1 2]-0-0-[0 2]0--0--1--[0 1]0-00[0 2]00-0---0[0 2][0 1]0??????????????????0[0 1]??00

Sinornithosaurus 011000101020?00000?01000?20?0011010010-0?[2 3][0 1]0?[2 3]1[0 1][0 1][0 1][0 1][0 1]10001[0 1][0 1]?1002[0 1 2]00-[0 1]?1003[0 1]30[0 1]0000000??00100[0 1][0 1]0[0 2]000000002[0 1]0000210-0---0010??????????????????[0 1]01[0 1]00

Graciliraptor ????????????????????1000??????????????????????????????????????????000?100[2 3]?[2 3]0000000000?00100[0 1][0 1]0[0 2]0?00-??02000000-0-0---0010????????????????????[1 2]???

Dromaeosaurus 0????00?1??000000050010002030010000010-1[1 2 3][1 2][0 1]03[1 3]110210201111[0 1]00100000-1[0 1]100[2 3][0 1]300000[0 1]11001[1 2][1 2]100000[0 1]00010000[0 1]000100-100---0010201110-000112----2??????

Atrociraptor 010000000?20000000400103?20300110000?0-[0 1]1[2 3]113[1 3]110[1 2]1010[0 1 2][0 1]1[1 2][1 2]20[0 1]00000-1[0 1][0 1][0 1]020?00000[0 1]00000[0 1][1 2]102[0 1]00[0 1]0001000020001[0 1]0-100---0010??????????????????00?00[0 1]

Bambiraptor 01??00??????0000004???03?2030?100000??????????????????????????????0-0[0 1]1003[0 1]3[0 1]00000000000010[0 1]0001000[0 1]-?002010000-0-0---0[0 2]1000000--1---12-----??????

Saurornitholestes 01?????????????00??101000?0200010100?0-002[0 1][0 1]331[0 1]021[0 1]200[1 2]1[0 1][1 2]20100000-1[0 1]1002[1 2][2 3]0[0 1]000[0 1]10[0 1]00[0 1][0 1]1[0 1][1 2]0001000000002010110-110---0010211110-00020-----1001000

Tsaagan 011100110021?00000400?00?20[2 3]0010000010-1?3[0 1][0 1]000-0--0-0-01-0-0000?00-1?1[0 1]02031-00---0000-11-0-0-10--0--00-00-000-0-0---000-????????????????????????

Velociraptor 011000110?21?00000[3 4]10?00?20[2 3]0011000010-00310300-0--0-0-01-0-?030?00-00[0 1][0 1]021?[0 1]000000000001100000[0 1]0000-0022000000-0-0---0[0 1 2]0-100110-00000-----1??????

Deinonychus 01000011000100000?3?110302020000000010-01[2 3]1[0 1][1 3]3110210?0011[0 1]120002000-1[0 1][0 1]0020200000[0 1]00000[0 1][1 2]1011001000[0 1][0 2]0[0 2][0 2]20101[0 1]0-1[0 1]0---0010200110-000012----0001100

'MPC_D100_1128' 0???1?????0?000?020????0?3001??00?00?1102222000-1-------------0000[1 2]1021[0 1 2][0 1]0001-1-------------------------------0-0-0---000-??????????????????1?0?0?

Almas 01??00000??0?00002221100?30112100001?110?222000-1-------------0000[1 2]10?[0 1][1 2]00001-1-------------------------------0-0-0---000-00000--1---22-----1???0?

Sinusonasus 0????0??1??0?00000121?00?20[0 1]?2010?01?[1 2]10?3[1 2][0 2]??0-1-------------0000[1 2]00?0[0 1 2]00??1-[0 1][0 1]---02?0-02-0-0-[0 2]0--0--1--0[0 1]-000-0-0---000-????????????????????????

Byronosaurus 0101000110?0000002021100?200100?0001?100?211010-1----1--------0000[1 2]00[1 2][1 2][0 1]0[0 1 2 3][0 1][2 4]1-1-------------------------------0-0-0---[1 3][1 2]0-00000--1---22-----??????

Zanabazar 010100000000000000[0 1]211000300100100011110?3112?1102?010222??0--0000[1 2]10[1 2][0 1][0 1]001[1 3][0 1]1000?00000[1 2][1 2]2?2?0?000?1??1221[0 1]0000-0-0---200-????????????????????????

Troodon 01????????????????????????00100?00011210221050[0 1][0 1]0010??332220000000[1 2]111[0 1][0 1]0[0 2][1 3 4]1[0 1][0 1]000100000[2 3][2 3][1 2]22[1 2]00[0 1]001100000010000-100---000-110110-00020-----11[0 1]1101

Epidexipteryx 01?000121020?0?0125--112??23?1200110?0-0?0[0 1]0?00-1-------------00000-0?[0 1]10?0?1-1-------------------------------0-0-0---000-??????????????????00??0[0 1]

Archaeopteryx 01?000011000?000[0 1]0500100?303001000[0 1]01100?310?00-1-------------0000100?[0 1][0 1]000?1-1-------------------------------0-0-0---000-??????????????????10?00?

Murusraptor ???????????????????????????????????????1[1 2][2 3]10?3100200???11???00000?0-1[0 1][1 2]003231-00---000[0 1]-21-0-0-100-0--02-01-000-0-0---0210??????????????????101100

Morphotype_2 ??????????????????????????????????????????????????????????????????0?1[1 2]1003231-00---000[0 1]-21-0-0-100-0--02-01-[0 1][0 1]0-[0 2]10---001???????????????????1?????

Morphotype_4 ??????????????????????????????????????????????????????????????????1100121000[0 1]0000000???0[0 1]1000000000[0 1]-??0?[0 1]10000-0-0---030?????????????????????????

Morphotype_5 ??????????????????????????????????????????????????????????????????0-1[0 1]?1001100000100?001[1 2]112001100110200[0 2]000110-0-0---000?????????????????????????

Morphotype_3 ?????????????????????????????????????0?112212?1100[1 2]0102110100200000-101100010000010000022112001100110[0 2]010001[1 2][1 2]0-0-0---000?????????????????????????

Morphotype_6 ????????????????????????????????????????????????????????????????????10?100?10000?[1 2]00?00331??????001?????0?0???0-0-0---000?????????????????????????

Morphotype_1 ??????????????????????????????????????????????????????????????????0-1100032311000110101-21-0-0-100-0--02-01-110-111010001???????????????????1?????

;

cnames

{0 Premaxillary_teeth present_in_the_anterior_and_posterior_portions_of_the_premaxilla absent_in_the_posterior_portion_of_the_premaxilla absent_in_the_anterior_portion_of_the_premaxilla absent_in_the_whole_premaxilla,_toothless_premaxilla;

{1 Highest_number_of_premaxillary_teeth_(or_alveoli): 3_or_less 4 5 6 7_or_more;

{2 Premaxillary_alveoli,_direction_of_main_axis_of_elongation_in_palatal_view all_alveoli_mesio-distally_oriented mesial_alveoli_labio-lingually_oriented,_distal_alveoli_mesio-distally_oriented all_alveoli_labio-lingually_oriented;

{3 Premaxillary_alveoli,_overlap_of_the_first_and_second_alveoli_in_palatal_view absent present,_partial present,_almost_complete;

{4 Premaxillary_alveoli,_overlap_of_the_second_and_third_alveoli_in_palatal_view absent present;

{5 Premaxillary_alveoli,_overlap_of_the_third_and_fourth_alveoli_in_palatal_view absent present;

{6 Premaxillary_teeth_(or_alveoli),_size all_approximately_equal_in_size distal_teeth_(or_alveoli)_smaller_than_mesial_teeth_(or_alveoli) mesial_teeth_(or_alveoli)_smaller_than_distal_teeth_(or_alveoli);

{7 Mesial_premaxillary_teeth_(or_alveoli),_size significantly_smaller_than_the_first_six_mesial_maxillary_teeth_(or_alveoli) subequal_in_size_than_the_first_six_mesial_maxillary_teeth_(or_alveoli) significantly_larger_than_the_first_six_mesial_maxillary_teeth_(or_alveoli);

{8 Distal_premaxillary_teeth_(or_alveoli),_size significantly_smaller_than_the_first_six_mesial_maxillary_teeth_(or_alveoli) subequal_in_size_than_the_first_six_mesial_maxillary_teeth_(or_alveoli) significantly_larger_than_the_first_six_mesial_maxillary_teeth_(or_alveoli);

{9 First_premaxillary_tooth_(or_alveolus),_size subequal_in_size_than_second_tooth_(or_alveolus) significantly_smaller_than_second_tooth_(or_alveolus) significantly_bigger_than_second_tooth_(or_alveolus);

{10 Second_premaxillary_tooth_(or_alveolus),_size subequal_in_size_than_third_(and_fourth)_premaxillary_tooth_(or_alveolus) significantly_smaller_than_third_(and_fourth)_tooth_(or_alveolus) significantly_larger_than_third_(and_fourth)_tooth_(or_alveolus);
{11 Distalmost_premaxillary_tooth_(or_alveolus),_mesiodistal_length_in_palatal_view subequal_in_size_than_more_mesial_teeth_(or_alveoli) significantly_smaller_than_more_mesial_teeth_(or_alveoli) significantly_larger_than_mesial_teeth_(or_alveoli);

{12 Distal_premaxillary_alveoli,_shape_in_palatal_view oval_to_subcircular subrectangular polygonal;

{13 Premaxillary_tooth_row,_distal_extension_(position_of_distalmost_premaxillary_tooth)_ aligned_(ventral)_to_external_naris mesial_to_external_naris_;

{14 Premaxilla_in_palatal_view unconstricted slightly_constricted strongly_constricted,_terminal_rosette_of_premaxilla;

{15 Subnarial_gap/diastema_(i.e.,_posterior_part_of_premaxillary_alveolar_margin_edentelous,_resulting_in_an_interruption_of_the_upper_tooth_row) absent_ present_and_short,_diastema_not_extensive_enough_to_host_more_than_one_tooth present_and_long,_diastema_extensive_enough_to_host_more_than_one_tooth;

{16 First_premaxillary_alveoli_open ventrally,_decumbent_teeth anteroventrally,_procumbent_teeth;

{17 Maxillary_teeth present_in_the_anterior_and_posterior_portions_of_the_maxilla_(posteriormost_portion_excluded) absent_in_the_anteriormost_portion_of_the_maxilla absent_in_the_posterior_portion_of_the_maxilla_(i.e.,_tooth_row_extending_only_on_the_anterior_75%_of_the_bone_or_less) absent_in_the_whole_maxilla,_toothless_maxilla;

{18 Highest_number_of_maxillary_teeth_(or_alveoli): >19 18-19 16-17 15 10-14 _1-9;

{19 Mesial_maxillary_teeth_(or_alveoli),_size subequal_in_size_than_distal_teeth_(or_alveoli) significantly_larger_than_distal_maxillary_teeth_(or_alveoli) significantly_smaller_than_distal_maxillary_teeth_(or_alveoli);

{20 Mid-maxillary_teeth_(or_alveoli),_mesiodistal_length subequal_in_size_than_mesialmost_maxillary_teeth_(or_alveoli) significantly_larger_than_mesialmost_maxillary_teeth_(or_alveoli) significantly_smaller_than_mesialmost_maxillary_teeth_(or_alveoli);

{21 First_maxillary_tooth_(or_alveolus),_size: significantly_smaller_than_second_tooth_(or_alveolus) subequal_in_size_than_second_tooth_(or_alveolus);

{22 First_maxillary_teeth_(or_alveoli)_open: ventrally,_decumbent_teeth anteroventrally,_procumbent_teeth;

{23 Mid-maxillary_teeth,_inclination: pointing_ventrally_(decumbent) pointing_lateroventrally_(laterocumbent) pointing_anteroventrally_(procumbent) pointing_posteroventrally_(retrocumbent);

{24 Maxillary_alveoli,_shape_of_most_of_them_in_palatal_view: oval_to_lenticular subrectangular circular merged_to_form_an_open_alveolar_groove_(interdental_septa_absent);

{25 Maxillary_tooth_row,_posterior_extension_(position_of_posteriormost_tooth) posterior_to_the_anteriormost_rim_of_orbit anterior_or_aligned_to_the_anteriormost_rim_of_orbit,_posterior_to_the_posteriormost_rim_of_antorbital_fenestra anterior_or_aligned_to_the_posteriormost_rim_of_antorbital_fenestra,_posterior_to_the_anteriormost_rim_of_antorbital_fenestra aligned_to_the_anteriormost_rim_of_antorbital_fenestra anterior_to_the_anteriormost_rim_of_the_antorbital_fenestra;

{26 Dentary_teeth present_in_the_anterior_and_posterior_portions_of_the_dentary absent_in_the_anteriormost_portion_of_the_dentary absent_in_the_posterior_portion_of_the_dentary_(i.e.,_tooth_row_extending_only_on_the_anterior_75%_of_the_bone_or_less) absent_in_the_whole_dentary,_toothless_dentary;

{27 Highest_number_of_dentary_teeth_(or_alveoli): >_25 18-25 15-17 <_15;

{28 Dentary_alveoli_in_dorsal_view: in_separate_alveoli merged_to_form_an_open_alveolar_groove_(interdental_septa_absent);

{29 Mesialmost_dentary_teeth_(or_alveoli),_size subequal_in_size_than_mid-_and_distal_dentary_teeth_(or_alveoli) significantly_larger_than_mid-_and_distal_dentary_teeth_(or_alveoli) significantly_smaller_than_mid-_and_distal_dentary_teeth_(or_alveoli);

{30 First_dentary_tooth_(or_alveolus),_size_in_comparison_to_second_and_third_dentary_alveoli: subequal_in_size first_tooth_(or_alveolus)_substantially_smaller_ first_tooth_(or_alveolus)_substantially_larger;

{31 Mid-dentary_teeth_(or_alveoli),_size subequal_in_size_than_mesial_maxillary_teeth_(or_alveoli) significantly_smaller_than_mesial_maxillary_teeth_(or_alveoli) significantly_larger_than_mesial_maxillary_teeth_(or_alveoli);

{32 Terminal_rosette_of_dentary,_highest_number_of_teeth_(or_alveoli): terminal_rosette_absent four_teeth_(or_alveoli) five_teeth_(or_alveoli);

{33 First_dentary_alveoli_open dorsally anterodorsally,_procumbent_teeth;

{34 Mid-dentary_teeth,_inclination pointing_dorsally pointing_anterodorsally,_procumbent;

{35 Dentary_teeth,_spacing evenly_spaced mesial_dentary_teeth_more_closely_appressed_than_those_in_middle_and_distal_parts_of_the_tooth_row;

{36 Palatal_teeth_on_the_pterygoid present absent;

{37 Mesial_teeth,_constriction_between_root_and_crown_in_most_crowns: absent constriction_weak,_base_of_crown_occupying_more_than_85%_of_largest_crown_width constriction_important,_base_of_crown_occupying_85%_or_less_of_largest_crown_width;

{38 Mesial_teeth,_constriction_between_root_and_crown_along_the_tooth_row: present_in_some_teeth present_in_all_teeth;

{39 Mesial_teeth,_height_of_the_largest_crown_(CH_in_centimetres)_in_subadult/adults: CH_?_1 1_<_CH_?_6 CH_>_6;

{40 Mesial_teeth,_labiolingual_compression_of_the_widest_crown_(CBR_=_CBW/CBL): CBR_<_0.5,_lenticular_and_strongly_labiolingually_compressed 0.5<CBR_?_0.75,_oval_to_lenticular weak,_0.75_<_CBR_<_1.2,_tooth_subcircular CBR_?_1.2,_teeth_labiolingually_elongated;

{41 Mesial_teeth,_baso-apical_elongation_of_the_most_elongated_crown_(CHR_=_CH/CBL): strongly_elongated,_CHR_>_3 important,_2.5_<_CHR_?_3 normal,_2_<_CHR_?_2.5 weak,_CHR_?_2;

{42 Mesial_teeth,_crown_recurvature_(lingually_or_distally) present,_strongly_recurved present,_slightly_recurved absent,_tooth_crown_straight_;

{43 Mesial_teeth,_distal_margin_of_the_crown_in_lateral_view mainly_concave straight mainly_convex,_apex_centrally_positioned_or_almost_centrally_positioned;

{44 Mesial_teeth,_outline_of_basal_cross-section_of_the_crown_in_the_mesialmost_tooth subcircular,_ovoid_or_elliptical lanceolate,_with_acute_and_well-developed_distal_carina Salinon_shape,_with_labial_margin_convex_and_lingual_margin_biconcave D-shaped_or_J-shaped,_with_lingual_margins_strongly_convex_and_labial_margin_convex_or_sigmoid U-shaped,_with_mesial_and_distal_margin_subparalell lenticular,_with_acute_and_well-developed_distal_and_mesial_carinae;

{45 Mesial_teeth,_concave_surface_adjacent_to_the_carina absent on_the_labial_surface_and_adjacent_to_the_distal_carina on_the_lingual_surface_and_adjacent_to_both_carinae on_the_lingual_surface_and_adjacent_to_the_mesial_carina_only on_the_lingual_surface_and_adjacent_to_the_distal_carina_only one_main_concave_surface_centrally_positioned_on_the_lingual_side_of_the_crown;

{46 Mesial_teeth,_mesial_carina absent present;

{47 Mesial_teeth,_mesial_carina non-denticulated denticulated;

{48 Mesial_teeth,_distal_carina denticulated non-denticulated;

{49 Mesial_teeth,_mesial_carina straight_and_centrally_positioned_on_the_crown slightly_twisted,_curves_onto_the_mesiolingual_surface strongly_twisted,_curves_onto_the_lingual_surface almost_straight_and_strongly_lingually_deflected;

{50 Mesial_teeth,_mesial_carina,_and_if_denticulated,_mesial_serration terminates_well-above_the_cervix extends_to_the_cervix_or_just_above_it terminates_well_beneath_the_cervix;

{51 Mesial_teeth,_distal_carina centrally_positioned_or_slightly_displaced strongly_labially_deflected;

{52 Mesial_teeth,_position_of_mesial_carina_on_the_crown_in_articulation_in_mesialmost_teeth facing_mostly_labially facing_mostly_mesially facing_mostly_lingually;

{53 Mesial_teeth,_position_of_distal_carina_on_the_crown_in_articulation_in_mesialmost_teeth facing_mostly_distally_or_labiodistally facing_mostly_lingually;

{54 Mesial_teeth,_average_number_of_denticles_per_five_millimetres_on_mesial_carina_at_two-thirds_height_of_the_crown_(MCA)_in_subadults/adults ?_20 14-19 9-13 ?_8;

{55 Mesial_teeth,_average_number_of_mid-crown_denticles_per_five_millimetres_on_distal_carina_(DC)_in_subadults/adults ?_20 14-19 9-13 ?_8;

{56 Mesial_teeth,_denticle_size_(except_in_embryos_and_hatchlings) minute_denticles,_more_than_250_denticles_on_the_distal_carina normal_in_height,_between_15_to_250_denticles_on_the_distal_carina very_larges_denticles,_less_than_15_denticles_on_the_distal_carina;

{57 Mesial_teeth,_denticles_on_mesial_carina rounded_and_symmetrically_convex_ rounded_and_asymmetrically_convex strongly_hooked/pointed,_denticles_with_a_tip_pointing_apically;

{58 Mesial_teeth,_denticles_on_distal_carina rounded_and_symmetrically_convex_ rounded_and_asymmetrically_convex strongly_hooked/pointed,_denticles_with_a_tip_pointing_apically;

{59 Mesial_teeth,_size_of_mesial_denticles_relative_to_distal_denticles_(DSDI) mesial_and_distal_denticles_of_same_size,_0.8_<_DSDI_<1.2 mesial_denticles_larger_than_distal_ones,_DSDI_<_0.8 distal_denticles_larger_than_mesial_ones,_DSDI_>_1.2;

{60 Mesial_teeth,_denticles_contiguous_over_tip_or_very_close_to_the_apex present absent_;

{61 Mesial_teeth,_interdenticular_sulci absent present,_short present,_long_and_well-developed;

{62 Mesial_teeth,_flutes_(i.e.,_subparallel_longitudinal_grooves_separated_by_acute_ridges)_on_the_crown absent present_on_the_lingual_surface_only present_on_both_labial_and_lingual_surfaces present_on_the_labial_surface_only;

{63 Mesial_teeth,_longitudinal_groove_on_the_labial_and/or_lingual_side_of_the_crown absent present,_a_single_groove_centrally_positioned present,_a_single_groove_mesially_positioned;

{64 Mesial_teeth,_longitudinal_ridge,_different_of_flutes,_on_the_lingual_side_of_the_crown absent present,_a_single_ridge_centrally_positioned present,_more_than_one_ridge;

{65 Mesial_teeth,_basal_striations,_different_of_flutes,_on_both_lingual_and_labial_sides_of_the_crown absent present;

{66 Lateral_teeth,_constriction_between_root_and_crown absent constriction_weak,_base_of_crown_base_occupying_more_than_85%_of_largest_crown_width_mesiodistally constriction_important,_base_of_crown_base_occupying_85%_or_less_of_largest_crown_width_mesiodistally;

{67 Lateral_teeth,_constriction_between_root_and_crown_along_the_tooth_row present_in_some_teeth present_in_all_teeth;

{68 Lateral_teeth,_height_of_the_largest_crown_(CH_in_centimetres)_in_subadults/adults CH_?_1 1_<_CH_?_6 CH_>_6;

{69 Lateral_teeth,_labiolingual_compression_of_the_crown_(CBR_=_CBW/CBL) important,_CBR_?_0.5,_tooth_strongly_flattened normal,_0.5_<_CBR_?_0.75 weak,_CBR_>_0.75,_tooth_incrassate_or_subcircular;

{70 Lateral_teeth,_baso-apical_elongation_of_the_crown_(CHR_=_CH/CBL) weak,_CHR_?_1.5 normal,_1.5_<_CHR_?_2.5 important,_CHR_>_2.5;

{71 Lateral_teeth,_distal_margin_of_crown_in_lateral_view strongly_concave slightly_concave,_roughly_straight,_or_straight,_apex_positioned_at_the_same_level_as_distal_profile convex,_apex_positioned_mesial_to_mesial_profile sigmoid,_basal_half_concave_and_apical_half_convex sigmoid,_basal_half_convex_and_apical_half_concave;

{72 Lateral_teeth,_mesial_margin_of_crown_in_lateral_view strongly_convex slightly_convex,_almost_straight;

{73 Lateral_teeth,_mesiodistal_curvature_of_the_labial_surface_of_the_crown_at_one_third_of_the_crown convex surface_centrally_positioned_on_the_crown_roughly_flattened surface_centrally_positioned_on_the_crown_concave,_labial_depression_restricted_to_the_crown_base surface_centrally_positioned_on_the_crown_concave,_labial_depression_extends_along_the_basal_half_of_the_crown_or_more_apically;

{74 Lateral_teeth,_concave_surface_adjacent_to_carinae_all_along_the_crown absent present_on_labial_surface_and_adjacent_to_distal_carina present_on_lingual_surface_and_adjacent_to_distal_carina present_on_labial_surface_and_adjacent_to_both_mesial_and_distal_carinae present_on_lingual_surface_and_adjacent_to_both_mesial_and_distal_carinae;

{75 Lateral_teeth,_outline_of_basal_cross-section_of_the_crown subcircular lenticular_or_lanceolate elliptical_or_bean-shaped_(i.e.,_longitudinal_depression_centrally_positioned_on_one_side_only) 8-shaped_(i.e.,_longitudinal_depression_centrally_positioned_on_both_lingual_and_labial_margins) Subrectangular;

{76 Lateral_teeth,_mesial_carina present absent;

{77 Lateral_teeth,_mesial_carina denticulated non-denticulated;

{78 Lateral_teeth,_distal_carina present absent;

{79 Lateral_teeth,_distal_carina denticulated non-denticulated;

{80 Lateral_teeth,_extension_of_mesial_carina_relative_to_distal_carina mesial_carina_extends_at_the_same_level_or_terminates_more_apically_than_the_distal_carina mesial_carina_extends_more_basally_than_the_distal_carina;

{81 Lateral_teeth,_mesial_carina,_and_if_denticulated,_basalmost_serration_of_the_mesial_carina terminates_around_mid-height_of_crown_or_more_apically extends_to_base_of_crown_or_slightly_above_the_cervix terminates_well_beneath_the_cervix;

{82 Lateral_teeth,_twisted_mesial_carina_in_some_crowns absent,_mesial_carina_centrally_positioned_on_mesial_margin_or_weakly_curved_lingually_towards_the_base_in_all_teeth present,_mesial_carina_strongly_twisting_onto_the_mesiolingual_surface_in_some_teeth;

{83 Lateral_teeth,_split_carina_in_some_teeth: absent present_in_the_mesial_carina present_in_the_distal_carina;

{84 Lateral_teeth,_distal_carina,_and_if_denticulated,_basalmost_serration_of_the_distal_carina extends_to_the_cervix_or_just_above_it terminates_well_beneath_the_cervix terminates_well_above_the_cervix;

{85 Lateral_teeth,_profile_of_the_distal_carina_on_the_crown_in_distal_view straight_or_very_slightly_bowed strongly_bowed_or_sigmoid;

{86 Lateral_teeth,_position_of_distal_carina_on_the_crown_in_distal_view centrally_positioned_or_slightly_displaced,_crown_subsymmetrical strongly_labially_deflected,_crown_asymmetrical;

{87 Lateral_teeth,_average_number_of_denticles_per_five_millimeters_on_mesial_carina_at_two-thirds_height_of_the_crown_(MCA)_in_subadults/adults: ?_30 16-29 9-15 ?_8;

{88 Lateral_teeth,_average_number_of_mid-crown_denticles_per_five_millimetres_on_distal_carina_(DC)_in_subadults/adults ?_30 16-29 9-15 ?_8;

{89 Lateral_teeth,_denticle_number_on_both_mesial_and_distal_carinae_(except_in_embryos_and_hatchlings) more_than_250_denticles_(minute_denticles_or_very_large_number_of_denticles_of_normal_size) between_15_to_250_denticles_(denticles_of_average_size)_ less_than_15_denticles_(very_large_denticles_or_very_small_number_of_small_denticles);

{90 Lateral_teeth,_shape_of_denticles_on_mesial_carina_in_lateral_view symmetrically_convex_ asymmetrically_convex hooked/pointed;

{91 Lateral_teeth,_shape_of_denticles_on_distal_carina_in_lateral_view symmetrically_convex_ asymmetrically_convex hooked/pointed;

{92 Lateral_teeth,_shape_of_mesial_margin_of_rounded_denticles_on_mesial_carina_in_lateral_view parabolic subrectangular,_with_flattened_surface;

{93 Lateral_teeth,_shape_of_distal_margin_of_rounded_denticles_on_distal_carina_in_lateral_view parabolic subrectangular,_with_flattened_surface semi-circular;

{94 Lateral_teeth,_shape_of_denticles_at_two-thirds_height_of_the_crown_(MC-MA)_on_mesial_carina_in_lateral_view longer_apicobasally_than_mesiodistally,_vertical_subrectangular_ as_long_mediodistally_as_apicobasally,_subquadrangular longer_mediodistally_than_apicobasally,_horizontal_subrectangular;

{95 Lateral_teeth,_shape_of_mid-crown_denticles_(DC)_on_distal_carina_in_lateral_view as_long_mediodistally_as_apicobasally,_subquadrangular longer_mediodistally_than_apicobasally,_horizontal_subrectangular longer_apicobasally_than_mesiodistally,_vertical_subrectangular_;

{96 Lateral_teeth,_denticle_size_along_the_carinae regular,_gradual_change_in_denticle_size irregular,_sporadic_change_in_denticle_size;

{97 Lateral_teeth,_biconvex_apical_denticles_(i.e.,_biconvex_external_margin_of_denticle)_on_mesial_carina_in_lateral_view absent present;

{98 Lateral_teeth,_orientation_of_mesiodistal_axis_of_apical_denticles_on_mesial_carina_in_lateral_view perpendicular_to_mesial_margin inclined_apically_from_mesial_margin;

{99 Lateral_teeth,_orientation_of_mesiodistal_axis_of_mid-crown_denticles_on_distal_carina_in_lateral_view perpendicular_to_distal_margin inclined_apically_from_distal_margin;

{100 Lateral_teeth,_average_number_of_denticles_on_mesial_carina higher_number_of_denticles_basally_than_at_the_mid-crown lower_number_of_denticles_basally_than_at_the_mid-crown subequal_number_of_denticles_basally_than_at_the_mid-crown;

{101 Lateral_teeth,_average_number_of_denticles_on_mesial_carina higher_number_of_denticles_apically_than_at_the_mid-crown lower_number_of_denticles_apically_than_at_the_mid-crown subequal_number_of_denticles_apically_than_at_the_mid-crown;

{102 Lateral_teeth,_average_number_of_denticles_on_distal_carina_(except_in_embryos_and_hatchlings) higher_number_of_denticles_basally_than_at_the_mid-crown subequal_or_lower_number_of_denticles_basally_than_at_the_mid-crown;

{103 Lateral_teeth,_average_number_of_denticles_on_distal_carina higher_number_of_denticles_apically_than_at_the_mid-crown lower_number_of_denticles_apically_than_at_the_mid-crown subequal_number_of_denticles_apically_than_at_the_mid-crown;

{104 Lateral_teeth,_size_of_mesial_denticles_relative_to_distal_denticles_(DSDI) mesial_and_distal_denticles_of_same_size,_0.8_<_DSDI_<1.2 mesial_denticles_larger_than_distal_ones,_DSDI_<_0.8 distal_denticles_larger_than_mesial_ones,_DSDI_>_1.2;

{105 Lateral_teeth,_distal_denticles_on_the_apex contiguous_over_tip,_or_very_close_to_the_apex distal_denticles_disappear_well_beneath_apex;

{106 Lateral_teeth,_interdenticular_space_between_mid-crown_denticles_on_the_distal_carina narrow,_less_than_one_third_of_the_denticle_width broad,_more_than_one_third_of_the_denticle_width;

{107 Lateral_teeth,_interdenticular_sulci_between_apical_denticles_on_the_mesial_carina absent present,_short_and_poorly_developed,_shorter_than_proximodistal_denticle_height_ present,_long_and_well-developed,_equal_or_longer_than_proximodistal_denticle_;

{108 Lateral_teeth,_interdenticular_sulci_between_mid-crown_denticles_on_the_distal_carina absent present,_short_and_poorly_developed,_shorter_than_proximodistal_denticle_height_ present,_long_and_well-developed,_equal_or_longer_than_proximodistal_denticle_;

{109 Lateral_teeth,_interdenticular_sulci_between_basalmost_denticles_on_the_distal_carina absent present,_short_and_poorly_developed,_shorter_than_proximodistal_denticle_height_ present,_long_and_well-developed,_equal_or_longer_than_proximodistal_denticle_;

{110 Lateral_teeth,_flutes_(i.e.,_subparallel_longitudinal_grooves_separated_by_acute_ridges)_on_the_crown absent present_on_the_lingual_surface present_on_labial_surface_or_both_labial_and_lingual_surfaces;

{111 Lateral_teeth,_average_number_of_flutes_on_the_crown 1-7 7-8 >8;

{112 Lateral_teeth,_large_transverse_undulations_on_the_crown_in_some_teeth absent present,_tenuous_and_barely_visible_with_light present,_pronounced_and_well_visible_with_light;

{113 Lateral_teeth,_large_transverse_undulations_on_the_crown_in_some_teeth_when_present just_a_few numerous_and_closely_packed;

{114 Lateral_teeth,_marginal_undulations_(i.e.,_short_undulations_adjacent_to_carinae)_in_some_teeth absent_ present_and_short,_the_mesiodistal_elongation_is_less_than_four_times_the_space_separating_each_undulation present_and_elongated,_the_mesiodistal_elongation_is_longer_than_four_times_the_space_separating_each_undulation;

{115 Lateral_teeth,_marginal_undulations_in_some_teeth present_and_shallow,_only_visible_with_light present_and_pronounced,_well_visible_in_lateral_view;

{116 Lateral_teeth,_marginal_undulations_in_some_teeth present_only_on_the_mesial_side_of_the_crown present_only_on_the_distal_side_of_the_crown present_on_both_mesial_and_distal_sides;

{117 Lateral_teeth,_marginal_undulations_in_some_teeth present_and_mesio-distally_oriented present_and_diagonally_oriented;

{118 Lateral_teeth,_longitudinal_groove_on_the_labial_and/or_lingual_surface_of_the_crown absent present,_a_single_groove_centrally_positioned present,_a_single_groove_adjacent_to_mesial_carina present,_two_grooves_or_more;

{119 Lateral_teeth,_elongated_longitudinal_and_rounded_ridge_(differing_from_flutes)_on_the_lingual_surface_of_the_crown absent present,_a_single_ridge_centrally_positioned present,_two_or_three_ridges present,_several_fainted_ridges;

{120 Enamel_surface_texture smooth_or_irregular_(non-oriented)_texture_ braided_(oriented)_texture_not_clearly_visible_with_light braided_(oriented)_texture_clearly_visible_with_or_without_light deeply_veined/anastomosed_(oriented)_texture;

{121 Coarse_enamel_surface_texture remains_baso-apically/diagonally_oriented_or_slightly_curved_basally_close_to_the_carinae strongly_curved_basally_close_to_the_carinae;

{122 Enamel_microstructure,_enamel_tubules absent_or_rare common_only_in_basal_unit_layer_(BUL)_and/or_inner_potion_of_enamel common_and_extend_throughout_entire_enamel_thickness extremely_common_and_forming_an_integral_structural_component_of_enamel;

{123 Enamel_microstructure,_predominant_enamel_type parallel_crystallites basal_unit_layer_(BUL) columnar;

{124 Enamel_microstructure,_predominant_enamel_type,_percentage_of_enamel_thickness ?_75% <_75%;

{125 Enamel_microstructure,_number_of_enamel_types_present_in_schmelzmuster one two three four;

{126 Enamel_microstructure,_number_of_different_module_types_present_in_schmelzmuster one two;

{127 Enamel_microstructure,_boundary_between_first_and_second_enamel_types_from_the_enamel-dentine_junction_(EDJ) parallel_to_EDJ jagged,_varies_in_distance_from_EDJ;

{128 Enamel_microstructure,_boundary_between_second_and_third_enamel_types_from_the_EDJ parallel_to_EDJ jagged,_varies_in_distance_from_EDJ;

{129 Enamel_microstructure,_basal_unit_layer_(BUL) present absent;

{130 Enamel_microstructure,_basal_unit_layer_(BUL) poorly_developed well-developed,_with_distinct_planes_of_separation_between_adjacent_units;

{131 Enamel_microstructure,_basal_unit_layer_(BUL),_maximum_unit_diameter <_10_µm ?_10_µm;

{132 Enamel_microstructure,_basal_unit_layer_(BUL) <_25%_of_total_enamel_thickness 25-50%_of_total_enamel_thickness ?_50%_of_enamel_thickness;

{133 Enamel_microstructure,_incremental_lines absent faint,_poorly_defined well-defined;

{134 Enamel_microstructure,_incremental_lines present_in_one_section_of_the_schmelzmuster_only present_in_more_than_one_section_of_the_schmelzmuster_but_not_throughout_entire_schmelzmuster present_throughout_entire_schmelzmuster;

{135 Enamel_microstructure,_columnar_units_closest_to_the_EDJ,_shape_of_units_in_cross-sections polygons_with_sharp_corners_and_more_than_4_sides subcircular_or_polygons_with_rounded_corners_and_more_than_4_sides triangles_and/or_rectangles_with_sharp_corners;

{136 Enamel_microstructure,_columnar_units_closest_to_the_enamel-dentine_junction_(EDJ) extend_straight_and_unbroken_to_the_OES_or_to_within_20_µm_below_the_OES end,_split,_or_are_interrupter_less_than_two-thirds_of_the_distance_from_the_EDJ_to_OES;

{137 Enamel_microstructure,_columnar_units_closest_to_the_enamel-dentine_junction_(EDJ),_maximum_unit_diameter <_15_µm ?_15µm;

{138 Enamel_microstructure,_columnar_units_closest_to_the_outer_enamel_surface_(OES) no_dominant_direction_of_orientation,_planes_of_separations_equally_well-developed_in_all_directions distinct_longitudinal_orientation,_planes_of_separation_better_developed_in_an_apicobasal_(longitudinal)_direction;

{139 Enamel_microstructure,_ratio_of_thickest_enamel_type_in_schmelzmuster_divided_by_second_thickest_enamel_type >_7 1.3_to_7 1_to_1.3;

{140 Root,_shape_in_lateral_view with_subparallel_mesial_and_distal_margins with_convex_margins,_root_significantly_larger_than_base_crown;

{141 Root,_distal_shape_in_lateral_view broad strongly_tapered_apically;

{142 Root,_outline_of_mid-root_in_cross_section oval_to_subcircular 8-shape_(i.e.,_longitudinal_depression_centrally_positioned_on_both_lingual_and_labial_margins) bean-shaped_(i.e.,_longitudinal_depression_centrally_positioned_on_one_side_only);

{143 Root,_form_of_the_resorption_pit_in_lingual_view deep_and_well-delimited_depression shallow_concavity_or_absent;

{144 Root,_transversal_undulations_below_the_cervix_in_some_crowns: absent present;

{145 Root,_apicobasal_height_in_lateral_view less_than_twice_the_apicobasal_height_of_the_crown twice_or_more_the_apicobasal_height_of_the_crown;

;

ccode + 0 1 3 14 15 18 25 27 37 39 49 56 66 68 89 *;

force + [0 1 2 3 4 5 6 7 8 9 10 11 12 13 14 15 16 17 18 19 20 21 22 23 24 25 26 27 28 29 30 31 32 33 34 35 36 37 38 39 40 41 42 43 44 45 46 47 48 49 50 51 52 53 54 55 56 57 58 59 60 61 62 63 64 65 66 67 68 69 70 71 72 73 74 75 76 77 78 79 80 81 82 83 84 85 86 87 88 89 90 91 92 93 94 95 96 97 98 99 100 101 102 103 104 105 106 107 (108 109 110 111 112 113)] [1 2 3 4 5 6 7 8 9 10 11 12 13 14 15 16 17 18 19 20 21 22 23 24 25 26 27 28 29 30 31 32 33 34 35 36 37 38 39 40 41 42 43 44 45 46 47 48 49 50 51 52 53 54 55 56 57 58 59 60 61 62 63 64 65 66 67 68 69 70 71 72 73 74 75 76 77 78 79 80 81 82 83 84 85 86 87 88 89 90 91 92 93 94 95 96 97 98 99 100 101 102 103 104 105 106 107 (108 109 110 111 112 113)] [3 4 5 6 7 8 9 10 11 12 13 14 15 16 17 18 19 20 21 22 23 24 25 26 27 28 29 30 31 32 33 34 35 36 37 38 39 40 41 42 43 44 45 46 47 48 49 50 51 52 53 54 55 56 57 58 59 60 61 62 63 64 65 66 67 68 69 70 71 72 73 74 75 76 77 78 79 80 81 82 83 84 85 86 87 88 89 90 91 92 93 94 95 96 97 98 99 100 101 102 103 104 105 106 107 (108 109 110 111 112 113)] [4 5 6 7 8 9 10 11 12 13 14 15 16 17 18 19 20 21 22 23 24 25 26 27 28 29 30 31 32 33 34 35 36 37 38 39 40 41 42 43 44 45 46 47 48 49 50 51 52 53 54 55 56 57 58 59 60 61 62 63 64 65 66 67 68 69 70 71 72 73 74 75 76 77 78 79 80 81 82 83 84 85 86 87 88 89 90 91 92 93 94 95 96 97 98 99 100 101 102 103 104 105 106 107 (108 109 110 111 112 113)] [5 6 7 8 9 10 11 12 13 14 15 16 17 18 19 20 21 22 23 24 25 26 27 28 29 30 31 32 33 34 35 36 37 38 39 40 41 42 43 44 45 46 47 48 49 50 51 52 53 54 55 56 57 58 59 60 61 62 63 64 65 66 67 68 69 70 71 72 73 74 75 76 77 78 79 80 81 82 83 84 85 86 87 88 89 90 91 92 93 94 95 96 97 98 99 100 101 102 103 104 105 106 107 (108 109 110 111 112 113)] [6 7 8 9 10 11 12 13 14 15 16 17 18 19 20 21 22 23 24 25 26 27 28 29 30 31 32 33 34 35 36 37 38 39 40 41 42 43 44 45 46 47 48 49 50 51 52 53 54 55 56 57 58 59 60 61 62 63 64 65 66 67 68 69 70 71 72 73 74 75 76 77 78 79 80 81 82 83 84 85 86 87 88 89 90 91 92 93 94 95 96 97 98 99 100 101 102 103 104 105 106 107 (108 109 110 111 112 113)] [7 8 9 10 11 12 13 14 15 16 17 18 19 20 21 22 23 24 25 26 27 28 29 30 31 32 33 34 35 36 37 38 39 40 41 42 43 44 45 46 47 48 49 50 51 52 53 54 55 56 57 58 59 60 61 62 63 64 65 66 67 68 69 70 71 72 73 74 75 76 77 78 79 80 81 82 83 84 85 86 87 88 89 90 91 92 93 94 95 96 97 98 99 100 101 102 103 104 105 106 107 (108 109 110 111 112 113)] [8 9 10 11 12 13 14 15 16 17 18 19 20 21 22 23 24 25 26 27 28 29 30 31 32 33 34 35 36 37 38 39 40 41 42 43 44 45 46 47 48 49 50 51 52 53 54 55 56 57 58 59 60 61 62 63 64 65 66 67 68 69 70 71 72 73 74 75 76 77 78 79 80 81 82 83 84 85 86 87 88 89 90 91 92 93 94 95 96 97 98 99 100 101 102 103 104 105 106 107 (108 109 110 111 112 113)] [8 9 10 11 12 13 14 15 16 17 18 19 20 21 22 23] [8 9] [10 11 12 13 14 15 16 17 18 19 20 21 22 23] [11 12 13 14 15 16 17 18 19 20 21 22 23] [11 12 13 14] [11 12] [13 14] [15 16 17 18 19 20 21 22 23] [16 17 18 19 20 21 22 23] [17 18 19 20 21 22 23] [18 19 20 21 22 23] [18 20 21] [20 21] [22 23] [24 25 26 27 28 29 30 31 32 33 34 35 36 37 38 39 40 41 42 43 44 45 46 47 48 49 50 51 52 53 54 55 56 57 58 59 60 61 62 63 64 65 66 67 68 69 70 71 72 73 74 75 76 77 78 79 80 81 82 83 84 85 86 87 88 89 90 91 92 93 94 95 96 97 98 99 100 101 102 103 104 105 106 107 (108 109 110 111 112 113)] [25 26 27 28 29 30 31 32 33 34 35 36 37 38 39 40 41 42 43 44 45 46 47 48 49 50 51 52 53 54 55 56 57 58 59 60 61 62 63 64 65 66 67 68 69 70 71 72 73 74 75 76 77 78 79 80 81 82 83 84 85 86 87 88 89 90 91 92 93 94 95 96 97 98 99 100 101 102 103 104 105 106 107 (108 109 110 111 112 113)] [25 26 27 28 29 30 31 32 33 34 35 36 37 38] [25 26] [27 28 29 30 31 32 33 34 35 36 37 38] [28 29 30 31 32 33 34 35 36 37 38] [29 30 31 32 33 34] [30 31 32 33 34] [30 31] [32 33 34] [33 34] [35 36 37 38] [35 36] [37 38] [39 40 41 42 43 44 45 46 47 48 49 50 51 52 53 54 55 56 57 58 59 60 61 62 63 64 65 66 67 68 69 70 71 72 73 74 75 76 77 78 79 80 81 82 83 84 85 86 87 88 89 90 91 92 93 94 95 96 97 98 99 100 101 102 103 104 105 106 107 (108 109 110 111 112 113)] [39 40 41 42 43 44 49 50 51 52 53] [39 40 41 42] [41 42] [43 44 49 50 51 52 53] [44 49 50 51 52 53] [49 50 51 52 53] [50 51 52 53] [51 52 53] [52 53] [45 46 47 48 54 55 56 57 58 59 60 61 62 63 64 65 66 67 68 69 70 71 72 73 74 75 76 77 78 79 80 81 82 83 84 85 86 87 88 89 90 91 92 93 94 95 96 97 98 99 100 101 102 103 104 105 106 107 (108 109 110 111 112 113)] [45 46 47 48 55 56 57 58 59 60 61 62 63 64 65 66 67 68 69 70 71 72 73 74 75 76 77 78 79 80 81 82 83 84 85 86 87 88 89 90 91 92 93 94 95 96 97 98 99 100 101 102 103 104 105 106 107 (108 109 110 111 112 113)] [45 46 47 48 56 57 58 59 60 61 62 63 64 65 66 67 68 69 70 71 72 73 74 75 76 77 78 79 80 81 82 83 84 85 86 87 88 89 90 91 92 93 94 95 96 97 98 99 100 101 102 103 104 105 106 107 (108 109 110 111 112 113)] [45 46 47 48 56 57 58 59 60 61 62 63 64 107 (108 109 110 111 112 113)] [56 57] [45 46 47 48 58 59 60 61 62 63 64 107 (108 109 110 111 112 113)] [45 46 47 48 59 60 61 62 63 64 107 (108 109 110 111 112 113)] [45 46 47 48 60 61 62 63 64 107 (108 109 110 111 112 113)] [45 46 47 48 107 (108 109 110 111 112 113)] [45 46] [47 48 107 (108 109 110 111 112 113)] [61 62 63 64] [62 63 64] [63 64] [65 66 67 68 69 70 71 72 73 74 75 76 77 78 79 80 81 82 83 84 85 86 87 88 89 90 91 92 93 94 95 96 97 98 99 100 101 102 103 104 105 106] [65 66 67] [68 69 70 71 72 73 74 75 76 77 78 79 80 81 82 83 84 85 86 87 88 89 90 91 92 93 94 95 96 97 98 99 100 101 102 103 104 105 106] [69 70 71 72 73 74 75 76 77 78 79 80 81 82 83 84 85 86 87 88 89 90 91 92 93 94 95 96 97 98 99 100 101 102 103 104 105 106] [69 70 71 72 73] [70 71 72 73] [71 72 73] [72 73] [74 75 76 77 78 79 80 81 82 83 84 85 86 87 88 89 90 91 92 93 94 95 96 97 98 99 100 101 102 103 104 105 106] [74 75 76 77] [75 76 77] [76 77] [78 79 80 81 82 83 84 85 86 87 88 89 90 91 92 93 94 95 96 97 98 99 100 101 102 103 104 105 106] [78 79 80 81 82] [79 80 81 82] [80 81 82] [81 82] [83 84 85 86 87 88 89 90 91 92 93 94 95 96 97 98 99 100 101 102 103 104 105 106] [83 84 85 86] [84 85 86] [85 86] [87 88 89 90 91 92 93 94 95 96 97 98 99 100 101 102 103 104 105 106] [87 88 89 90 91 92 93 94 95 96 97 98] [88 89 90 91 92 93 94 95 96 97 98] [89 90 91 92 93 94 95 96 97 98] [89 90] [91 92 93 94 95 96 97 98] [92 93 94 95 96 97 98] [93 94 95 96 97 98] [94 95 96 97 98] [94 95] [96 97 98] [97 98] [99 100 101 102 103 104 105 106] [99 100 101 102 103 104] [99 100] [101 102 103 104] [102 103 104] [103 104] [105 106]

proc /;

comments 0

;

### Crown-based data matrix

xread

91 109

Herrerasaurus 0-11[2 3]10000-0--1-0-01-0-?000010-1[0 1 2][0 1][0 1]000[0 2]00000?00000111000[0 1]100000-???0?10000-200---000-??????

Daemonosaurus 0-1?[2 3]000011000010??110?0000000-1?[1 2]0000?00000000000??11000010?000000?0?0000-0-0---000-00????

Eodromaeus ?????????????????????????????0-0[0 1]200011[0 1]0000100000001[0 1]000[0 1][0 1]000020000?00000-100---0010??????

Eoraptor 100[1 2][1 2]100[0 1][0 1]10001101012[1 2]1100000[1 2]00[0 1][0 1]100[1 3]?00000000?001[0 1]1[1 2][1 2]00000001--001000000-0-0---000-??????

Dracovenator 0-1021010110000???21?020000000-10?000010000010010012100000100000?0?2?10000-100---0010????0?

Coelophysis 0-0[0 1][0 1 2]1000[0 1]1000110-01-0-0030000-1[0 1][0 1]000[1 3]100000[0 1]000??00100[0 1]0[0 1]00000-000000000[0 3]00-0---0010??????

Liliensternus 0-01?1[0 1]0011000010001?00?000000-1[0 1]1[0 1]000100000000000[0 1][0 1]100[0 1]0[0 1][0 1]0?00-0000010000-0-0---000-001?0?

Dilophosaurus 0-11[0 1]0000110001??1[1 2]10020000000-11100[1 2][3 4]1000001000001210000[0 1]100000?0?20?0000-0-0---0010001000

Ceratosaurus 0-1[1 2]3[0 1][0 1]0011000010[2 3][2 3]10020[0 1]10000-2[0 1]1[0 1 2]0[1 2][0 3 4]10000010000[0 1]22100[0 1]01[0 1 2]001[0 1]0[1 2]0[0 2]001[0 1][0 1 2][0 1 2]0-[1 2]01[0 1][0 1][0 1]001000?000

Genyodectes 0-2[0 1][0 1 2]000011000000221000?000000-2[0 1]1[0 1]0[1 2][2 3 4]2000001000[0 1]12210[0 1]0010001[0 1]-2020010[0 1]00-210---0010??????

Berberosaurus ?????????????????????????????0-10100[1 2]2300000100?01221[0 1][0 1]???1001?-1020?10000-200---0010??????

'Limusaurus_juvenile' 0-0??[0 1]???0-1-------------00000-0??[0 1]0???1-1-------------------------------0-0-0---00??0[0 1]???0

Noasaurus ?????????????????????????????0-01[0 1]100010000010000001100000000000?1?2?00000-0-0---000-??????

Masiakasaurus 210[2 3][0 1]10231[0 1]02112000100[0 2]0010000-1[0 1][1 2]0000100000100001[0 1][1 2]10[0 1 2][0 1]000000[0 1]000020000010[1 2]00---0010001100

Kryptops ?????????????????????????????0-1?1100010000010000?121[0 1]2[0 1]0000001?0?000022?0-0-0---000-0?????

Rugops ???1???2?11?0?010????????????0-1[0 1]?[1 2]000100000100000[1 2]210200000001?0?000000?0-0-0---000-??????

Abelisaurus 0-113[1 2][1 2]231100[1 2]0??2210[1 2]00200000-1[0 1][0 1]1000100000100?00221[0 1][1 2]0010011100000000100-101010000-??????

Aucasaurus ?????????????????????????????0-1[0 1][0 1]1000100000100000221[0 1][1 2][0 1]01[0 1]011100?00000000-200---000-0?0?1?

Arcovenator ?????????????????????????????0-1[0 1]10000100000100101[1 2]21[0 1][0 1]0[0 2][0 1]1000100012000000-0-0---000-0?????

Chenanisaurus 1?1[1 2]2[0 1][1 2]2311002010331[0 1][0 1]00200001?1[0 1]110??100000200110221[0 1][0 1]00110?01000000012?0-?00---000-00???0

Indosuchus 0-1[1 2]3[1 2][1 2][2 3][2 3]110[0 1][1 2]010[2 3][2 3]1??0?000000-1[0 1]?[1 2]0001000001000?0221??????????0???0??0110-0-0---000-??????

Majungasaurus [0 1]01[1 2]3[1 2][1 2][2 3][2 3]110[0 1]2010[2 3][2 3]1[1 2]200200000-1[0 1][0 1][1 2]000100000100000[2 3]21[1 2]2000[0 1]000100000001220-201010000-00?1??

Skorpiovenator 0-11222?3110020??211???0000000-10[0 1][1 2]000100000100010221000011001[0 1]0?0?0?00000-0-2020000-00?1?0

Chilesaurus 210?122?00-1-------------0000110?12000?11[0 1][0 1]----200?0??0?0?2?0?0??--?00?0-0-0-0---00??10??00

Piatnitzkysaurus ?????????????????????????????0-11[1 2]0010100000[0 1]10[0 1]10[1 2][1 2]10[0 1 2]0[0 2][0 1]1000020002000[1 2][1 2]0-[1 2]1201000[0 1]0001100

Marshosaurus 0-1121[0 1]10110000100110020100000-1[0 1][0 1]0000100000000?0[0 1]1[1 2]001[0 1]00[0 1]000000002010100-[1 2][1 2]0---001000?1?0

Monolophosaurus 0-11[2 3][0 1]0101?000110?11?0??000000-1[0 1][0 1][0 1]000100000000[0 1]0[0 1][1 2][1 2]100[0 1][0 2]0[0 1]0000?0?00010000-10101100110??1??

Sciuruminus 0-0?[1 2][0 1]0??0-[0 1]--?-0-0?-?-100000100?100???1-00---00??-??-[1 2]-0-[0 2]0--0--00-[0 1]1-000-0-0---000-1???0?

Eustreptospondylus ??1??10?01100?00122100?0100000-11?0000100000000???[2 3]2100[0 1]0000000-??00000??0-0-0---0010??????

Afrovenator ?????????????????????????????0-2010001100000000110321[0 1][0 1][0 1]2010?00-0011001110-0-100000100?1?0?

Dubreuillosaurus 0-11210101100000[0 1]221000?000000-1[0 1][0 1]0000100000000100111[0 1][0 1][0 1]0000000-0010000110-0-0---0010000?00

Duriavenator 0-?12??1?110??0?1[2 3]310000?00000-1[0 1]100001000000001??[2 3]210[0 1][0 1]00101[0 1]0-101[0 1]000110-[0 1]00---001000?100

Megalosaurus ?????????????????????????????0-2[0 1]100001000000001[0 1]0[2 3][2 3]1[0 1][0 1][0 1][0 2]010100-100[0 1]001[1 2][1 2]0-2110[1 2][0 1]0020??????

Torvosaurus ??21???1011000000331???0?00000-2[0 1][1 2]0000100000[0 1]001[0 1]0[2 3][2 3]1[0 1][0 1][0 1]0110?00-101[0 1]0[0 1]1220-2[0 1]10100020002100

Baryonyx 0-12[1 2]1000110020??0000000010000-121[0 1]000000000[1 2]001[0 1]000[0 1]00[0 1][0 1]2111000202[0 2]00000[1 2]00-10100031010111

Suchomimus 0-???1000110020??00000000[1 2]0000-1[1 2][1 2][0 1]000000000[1 2]001[0 1]0[0 1][0 1][0 1]00[0 1][0 1]1010002202[0 2]00000[1 2]00-10[1 2][0 1]0031??????

'Irritator_Angaturama' ?????????????????????????????0-12[1 2]2100001010100100-----------------------210-101[0 1]000-??????

Spinosaurus 0-?[1 2]??1001010[1 2]001--------20000-2[1 2][1 2][1 2]100001010[0 1]00100-----------------------220-0---00310101?[0 1]

Erectopus ?????????????????????????????0-10[1 2]001010000010000022100[0 1]011010000000000100-1[0 1]0---000-??????

Yangchuanosaurus 0-1?[1 2]11??110[1 2]?0?0?21?0???????0-2?2[0 1]0??20000???????22???????????????????????????????????????

Sinraptor_dongi 0-1[1 2 3]?11[2 3][1 3]1101[1 2]0102210[0 1]00[1 2]02000-2[0 1][0 1][0 1]0[1 2][0 1 2][2 3]00000[1 2]00[0 1][0 1][0 1]221[0 1][0 1]00[1 2]1000000000011[1 2][0 1]0-211010000-001[0 1]01

Sinraptor_hepingensis 0-1?1[1 2][1 2]??110???10????????????0-201[0 1]0[1 2]0?00000[1 2]0?1?12210[0 1]00110?00?000001???0-????????0-00?0?1

Allosaurus 0-1[2 3][0 1 2][0 1][0 1][2 3][2 3]110[1 2]10102[2 3]1[0 1][0 1]00[0 1 2]0[0 2][0 1]00-2[0 1 2][1 2][0 1]000[0 1 2]00000[1 2]11[0 1][0 1]1[1 2][1 2]1[0 1][0 1][0 1]011000[0 1]00000011[1 2][1 2]0-2[0 1]2[0 1]1[0 1]00[0 1]00[0 1][0 1][0 1]1[0 1]

Neovenator ?????????????????????????????0-111001[1 2]20000000011[0 1]2[1 2]11100110010-0000011110-2[0 1]2010002010211[0 1]

Fukuiraptor 0-1[1 2]?0030110210??[0 1]110[0 1]0??00000-1[0 1][0 1 2]00??100000[0 1]00010[1 2][1 2]1[0 1][0 1]000[0 1]0?0000000002220-[1 2]020?000????????

Australovenator 0-1[1 2][2 3]00?3110210??1[1 2]1?????00000-1[0 1]100[2 3][1 2]?000000000?[0 1]221?[0 1]?00[0 1]0??0-000001???0-??????0020??????

Megaraptor ?????10?3100200??-11-0-00000?0-1[0 1][0 1]002231-00---000[0 1]-11-0-0-100-0--00-01-000-100---0010100100

Orkoraptor ?????????????????????????????0-1??003231-00---0[0 1][0 1][0 1]-21-0-0-100-0--0?-?1-110-??0---0[0 2]100?1???

Acrocanthosaurus 0-2[1 2][1 2]100011000[0 1]00[1 2]210020000000-2[0 1][0 1 2]0000?00000[0 1]0011[0 1][1 2][1 2][0 1]001[0 1]110111010[1 2][0 1]010[0 1]00-2[0 1][1 2][0 1]100020??????

Eocarcharia ?????????????????????????????0-101[0 3]00020000000011[0 1]211[0 1][0 1]0[0 2][1 2]10110-1011001110-100---0010??????

Carcharodontosaurus ?????????????????????????????102[0 1]1[1 2 3]000100001200110[2 3][2 3]1110010011[0 1]00001001[0 1][0 1]0-[0 1]021200010001100

Giganotosaurus 0-02?110011001000221?00?10000102[0 1][1 2][0 3]000100000[1 2]00110[2 3][2 3]1[0 1][0 1]001[0 1]00000000101[1 2]220-[1 2]0[1 2]020001000[1 2]100

Mapusaurus 0-12[2 3]10001101?1??221??0000000102[0 1][0 1][0 1 3]000100000[1 2]00110[2 3][2 3]1[0 1][0 1]001100000000[0 1]01[1 2]220-0-[1 2][0 1]200010[0 1]0[1 2]100

Bicentenaria 0-?[1 2]???00??0??0?0?0??0??000000?0?100?0100000100000001000001000000002000000-0-0---00200000??

Aorun 0-0?[0 2]00[0 1]?0-1-------------00000-0[0 1]?000?11-[0 1]1---0???-01-0-0-[0 2]0--0--?--10-0?0-0-0---0020??????

Zuolong 0?1?[2 3]2140110301??0010020000000??[0 1]?[1 2]0?0?00000000[0 1]??1[0 1]10000?10?01-?0???1?000-1?0---001000[1 2]?10

Proceratosaurus 100?[2 3]103[0 3]1102011[1 2]0010020000010-1[0 1][0 1]00[1 2]0[1 3]00000[0 1]100[0 1]10[0 1]10000000000-0002000[0 1][0 1]0-0-0---0010??????

Guanlong 0-1[1 2 3]3[1 2]13[0 3]11021121[1 2]11[0 1][0 1]00000000-1[0 1][0 1]00001[0 1]0000[0 1][0 1]00011110[0 1]1[0 2]010000-0?02010[0 1]?0-100---0010??????

Dilong 0-0[2 3][0 1 2 3][1 2][0 1]4[0 3][0 1]11[1 3]012000100[0 2]0000100-1[0 1 2][0 1 2][0 1]0[0 2]0?000000000011110[0 1][0 1][0 2]0[0 1 2]0000-0002010[0 1]0[0 1]2100---00[1 2]011[1 2]10[0 1]

Eotyrannus 0-1311[0 1]43110311??[1 2][1 2]10000100000-111[0 1]0001000000001001[1 2]10000000000-0002000110-0-0---0010??1???

Raptorex 0-03[0 1]104211031121??10020000100-?[0 1][0 1]00[0 1]0100000[0 1]10[0 1][0 1]1??100[0 1][0 2]1[0 1]000000000000100-100---00100?1000

Gorgosaurus 0-1[2 3][0 1 2 3]1142110[2 3]1[0 1][1 2][0 1]1[1 2]1?000000[0 1]00-1[0 1 2][1 2][0 1]000[0 1]00000[0 1]10[0 1]01[1 2][1 2]100[0 1]01[0 1]00000[0 2]0[0 2]00111[0 1]0-1[0 1]0---0010001000

Alioramus ?????????????????????????????0-?[0 1][0 1]00[1 2]0[1 3]00000[0 1]10[0 1]01??100001[0 1]000[0 1]00000000100-210---0010001000

Daspletosaurus 0-13[0 1]11421[0 1][0 1][2 3][0 1][0 1]2[0 1]221[0 1]0[0 2]0100100-2[0 1 2][0 1 2][0 1]000[0 1]00000111[0 1]0[0 1][2 3]2111[0 1]01[0 1]00000[0 2]0[0 2]0011[1 2]10-[1 2]110000010[0 1][0 1][0 1][0 1]1[0 1]

Tyrannosaurus 0-233[0 1]04[0 2]11031[0 1][1 2][0 1][2 3][2 3]10000000[0 1]00-2[1 2][0 1 2][0 1]000000000[0 1]11001[2 3][2 3]100001[0 1]0100020200122?0-2[0 1]112[0 1]00[0 1]0001[0 1]0[0 1]

Compsognathus 0-0?3[0 1 2]0?00-1-------------00000-0[0 1][0 1]0000?1-0[0 1]---02??-01-0-[0 1]-20--0--00-10-000-0-0---000-10?000

Juravenator 0-0?[0 1 2]00??0-[0 1]--?-0-0--0-1000000-0?[1 2]00?011-01---02??-01-0-0-[0 2]0--?--[0 1]0-[0 1]0-000-0-0---000-10??00

Scipionyx 0-??2000?0-1-------------10000-??[0 1 2][0 1]02011-00---0[0 2]??-?--0-0-[0 2]0--0--?0-10-000-0-0---000-??????

Ornitholestes 100[1 2 3]21[0 1][3 4][0 3][0 1]0111010--------00000-0[1 2][0 1]0[0 1]001[0 1]10[0 1]?100201-01-0-0-00--0--?0-1?-0-0-[0 1]00---00100?[0 1]??1

Nqwebasaurus ?????????????????????????????0-022210001-1-------------------------------0-0-0---000-??????

Pelecanimimus 110??1[0 1]3?0-1-------------0000[1 2]10?0[0 1][0 1]00?1-1-------------------------------0-0-0---000-0?0?0?

Shenzhousaurus 0-02?22000-1-------------00000-021210001-1-------------------------------0-0-0---000-??????

Haplocheirus 000?210000-1--0-0--------00000-0[1 2][1 2]0000?[0 1]00000000??0[0 1]1000000000[0 1]-??0?[0 1]10000-0-0---030-??????

Shuvuuia 1102?22000-1-------------00?011021210001-1-------------------------------0-0-0---000-0?0???

Mononykus ?????????????????????????????1?021210001-1-------------------------------0-0-0---030-0?0?0?

Eshanosaurus ?????????????????????????????21021[1 2]000000000000200112[0 1][0 1 2]00[0 2][0 2]0000-[1 2]-20010000-0-0---0[0 1]0-1?0?0?

Falcarius 1002[2 3]220[0 5]001-------------0000210[1 2][0 1][2 4]00[0 2 3]0000000102000010000[0 1][0 2]0000-1-1[0 2]00[0 2][0 2]00-100---0[0 1]0-[0 1][0 1]010[0 1]

Jianchangosaurus 211221004110201201?2220000?0021021[2 4]00[0 3]0000000002002222200000011-2-20010000-0-0---000-110?01

Segnosaurus ?????????????????????????????2112[1 2][1 2][0 1][0 2][0 4]0000000[0 1]22[0 2]0222[0 1 2][0 1 2]00[0 1]2[0 1]0[0 1][0 1]-0-00[0 1]10000-100---[0 3][0 1]1-0?????

Erlikosaurus 1002222000-0--0-0-02-1-?00000210212000000000000200??2[0 2][0 2]00020011-2-20010000-0-0---000-110??1

Incisivosaurus 001?22[1 2]001010?010--------00001?0?021000[0 1]?[0 1][0 1]??00200-?01?0?0?10??0??-???0?0--0-0---00[0 3]0??????

Caudipteryx 0-0?0[0 2][0 1]?-0-1-------------0000------------------------------------------------------0-10[1 2]00[0 1]

Halszkaraptor 1?0??003-0-1-------------00?00-0??000011-1-------------------------------0-??????00??0[0 1]?0?0

Buitreraptor ?????????????????????????????0-0[0 1][0 1]003[0 1]31-1-------------------------------0-0-0---[1 3][1 2]0-1?1?00

Microraptor 0-0?[1 2][0 1]0?[0 3]0-1--0-0--------00[0 1 2]0100?1[0 1]03131-0[0 1]---0[0 2]??-0[1 2]-0-0-[0 2]0--0--1--[0 1]0-00[0 2]00-0---0[0 2][0 1]00[0 1]??00

Sinornithosaurus 0-0?[2 3][0 1]0?[2 3]1[0 1][0 1][0 1][0 1][0 1]10001[0 1][0 1]?1002[0 1 2]00-[0 1]?1003[0 1]30[0 1]0000000??00100[0 1][0 1]0[0 2]000000002[0 1]0000210-0---0010[0 1]01[0 1]00

Graciliraptor ?????????????????????????????000?100[2 3]?[2 3]0000000000?00100[0 1][0 1]0[0 2]0?00-??02000000-0-0---0010??[1 2]???

Dromaeosaurus 0-1[1 2 3][1 2][0 1]03[1 3]110210201111[0 1]00100000-1[0 1]100[2 3][0 1]300000[0 1]11001[1 2][1 2]100000[0 1]00010000[0 1]000100-100---0010??????

Atrociraptor 0-[0 1]1[2 3]113[1 3]110[1 2]1010[0 1 2][0 1]1[1 2][1 2]20[0 1]00000-1[0 1][0 1][0 1]020?00000[0 1]00000[0 1][1 2]102[0 1]00[0 1]0001000020001[0 1]0-100---001000?00[0 1]

Bambiraptor ?????????????????????????????0-0[0 1]1003[0 1]3[0 1]00000000000010[0 1]0001000[0 1]-?002010000-0-0---0[0 2]10??????

Tsaagan 0-1?3[0 1][0 1]000-0--0-0-01-0-0000?00-1?1[0 1]02031-00---0000-11-0-0-10--0--00-00-000-0-0---000-??????

Velociraptor 0-00310300-0--0-0-01-0-?030?00-00[0 1][0 1]021?[0 1]000000000001100000[0 1]0000-0022000000-0-0---0[0 1 2]0-??????

Deinonychus 0-01[2 3]1[0 1][1 3]3110210?0011[0 1]120002000-1[0 1][0 1]0020200000[0 1]00000[0 1][1 2]1011001000[0 1][0 2]0[0 2][0 2]20101[0 1]0-1[0 1]0---0010001100

Saurornitholestes 0-002[0 1][0 1]331[0 1]021[0 1]200[1 2]1[0 1][1 2]20100000-1[0 1]1002[1 2][2 3]0[0 1]000[0 1]10[0 1]00[0 1][0 1]1[0 1][1 2]0001000000002010110-110---0010001000

'MPC_D100_1128' 1102222000-1-------------0000[1 2]1021[0 1 2][0 1]0001-1-------------------------------0-0-0---000-1?0?0?

Almas 110?222000-1-------------0000[1 2]10?[0 1][1 2]00001-1-------------------------------0-0-0---000-1???0?

Sinusonasus [1 2]10?3[1 2][0 2]??0-1-------------0000[1 2]00?0[0 1 2]00??1-[0 1][0 1]---02?0-02-0-0-[0 2]0--0--1--0[0 1]-000-0-0---000-??????

Byronosaurus 100?211010-1----1--------0000[1 2]00[1 2][1 2][0 1]0[0 1 2 3][0 1][2 4]1-1-------------------------------0-0-0---[1 3][1 2]0-??????

Zanabazar 110?3112?1102?010222??0--0000[1 2]10[1 2][0 1][0 1]001[1 3][0 1]1000?00000[1 2][1 2]2?2?0?000?1??1221[0 1]0000-0-0---200-??????

Troodon 210221050[0 1][0 1]0010??332220000000[1 2]111[0 1][0 1]0[0 2][1 3 4]1[0 1][0 1]000100000[2 3][2 3][1 2]22[1 2]00[0 1]001100000010000-100---000-1[0 1]1101

Epidexipteryx 0-0?0[0 1]0?00-1-------------00000-0?[0 1]10?0?1-1-------------------------------0-0-0---000-00??0[0 1]

Archaeopteryx 100?310?00-1-------------0000100?[0 1][0 1]000?1-1-------------------------------0-0-0---000-10?00?

MorphoI [0 1]?1[1 2]2[0 1][1 2][2 3][2 3]1100[1 2]0102210[1 2][0 1]0[1 2]0000??????????????????????????????????????????????????????0-??????

MorphoII 1?103212[2 3]1100[1 2]010[1 2]110[0 1][0 1]0[1 2]0000??????????????????????????????????????????????????????0-??????

MorphoIII ?????????????????????????????0?1[0 1][0 1][0 1]000100000[1 2]001[0 1]0[1 2]110[1 2]000[0 1]000[0 1]0?0[0 2]00001?0-0-0-----0-??????

Murusraptor ??1[1 2][2 3]10?3100200???11???00000?0-1[0 1][1 2]003231-00---000[0 1]-21-0-0-100-0--02-01-000-0-0---0210101100

Morphotype_2 ?????????????????????????????0?1[1 2]1003231-00---000[0 1]-21-0-0-100-0--02-01-[0 1][0 1]0-[0 2]10---001?1?????

Morphotype_4 ?????????????????????????????1100121000[0 1]0000000???0[0 1]1000000000[0 1]-??0?[0 1]10000-0-0---030???????

Morphotype_5 ?????????????????????????????0-1[0 1]?1001100000100?001[1 2]112001100110200[0 2]000110-0-0---000???????

Morphotype_3 0?112212?1100[1 2]0102110100200000-101100010000010000022112001100110[0 2]010001[1 2][1 2]0-0-0---000???????

Morphotype_6 ???????????????????????????????10?100?10000?[1 2]00?00331??????001?????0?0???0-0-0---000???????

Morphotype_1 ?????????????????????????????0-1100032311000110101-21-0-0-100-0--02-01-110-111010001?1?????

;

cnames

{0 Mesial_teeth,_constriction_between_root_and_crown_in_most_crowns: absent constriction_weak,_base_of_crown_occupying_more_than_85%_of_largest_crown_width constriction_important,_base_of_crown_occupying_85%_or_less_of_largest_crown_width;

{1 Mesial_teeth,_constriction_between_root_and_crown_along_the_tooth_row: present_in_some_teeth present_in_all_teeth;

{2 Mesial_teeth,_height_of_the_largest_crown_(CH_in_centimetres)_in_subadult/adults: CH_?_1 1_<_CH_?_6 CH_>_6;

{3 Mesial_teeth,_labiolingual_compression_of_the_widest_crown_(CBR_=_CBW/CBL): CBR_<_0.5,_lenticular_and_strongly_labiolingually_compressed 0.5<CBR_?_0.75,_oval_to_lenticular weak,_0.75_<_CBR_<_1.2,_tooth_subcircular CBR_?_1.2,_teeth_labiolingually_elongated;

{4 Mesial_teeth,_baso-apical_elongation_of_the_most_elongated_crown_(CHR_=_CH/CBL): strongly_elongated,_CHR_>_3 important,_2.5_<_CHR_?_3 normal,_2_<_CHR_?_2.5 weak,_CHR_?_2;

{5 Mesial_teeth,_crown_recurvature_(lingually_or_distally) present,_strongly_recurved present,_slightly_recurved absent,_tooth_crown_straight_;

{6 Mesial_teeth,_distal_margin_of_the_crown_in_lateral_view mainly_concave straight mainly_convex,_apex_centrally_positioned_or_almost_centrally_positioned;

{7 Mesial_teeth,_outline_of_basal_cross-section_of_the_crown_in_the_mesialmost_tooth subcircular,_ovoid_or_elliptical lanceolate,_with_acute_and_well-developed_distal_carina Salinon_shape,_with_labial_margin_convex_and_lingual_margin_biconcave D-shaped_or_J-shaped,_with_lingual_margins_strongly_convex_and_labial_margin_convex_or_sigmoid U-shaped,_with_mesial_and_distal_margin_subparalell lenticular,_with_acute_and_well-developed_distal_and_mesial_carinae;

{8 Mesial_teeth,_concave_surface_adjacent_to_the_carina absent on_the_labial_surface_and_adjacent_to_the_distal_carina on_the_lingual_surface_and_adjacent_to_both_carinae on_the_lingual_surface_and_adjacent_to_the_mesial_carina_only on_the_lingual_surface_and_adjacent_to_the_distal_carina_only one_main_concave_surface_centrally_positioned_on_the_lingual_side_of_the_crown;

{9 Mesial_teeth,_mesial_carina absent present;

{10 Mesial_teeth,_mesial_carina non-denticulated denticulated;

{11 Mesial_teeth,_distal_carina denticulated non-denticulated;

{12 Mesial_teeth,_mesial_carina straight_and_centrally_positioned_on_the_crown slightly_twisted,_curves_onto_the_mesiolingual_surface strongly_twisted,_curves_onto_the_lingual_surface almost_straight_and_strongly_lingually_deflected;

{13 Mesial_teeth,_mesial_carina,_and_if_denticulated,_mesial_serration terminates_well-above_the_cervix extends_to_the_cervix_or_just_above_it terminates_well_beneath_the_cervix;

{14 Mesial_teeth,_distal_carina centrally_positioned_or_slightly_displaced strongly_labially_deflected;

{15 Mesial_teeth,_position_of_mesial_carina_on_the_crown_in_articulation_in_mesialmost_teeth facing_mostly_labially facing_mostly_mesially facing_mostly_lingually;

{16 Mesial_teeth,_position_of_distal_carina_on_the_crown_in_articulation_in_mesialmost_teeth facing_mostly_distally_or_labiodistally facing_mostly_lingually;

{17 Mesial_teeth,_average_number_of_denticles_per_five_millimetres_on_mesial_carina_at_two-thirds_height_of_the_crown_(MCA)_in_subadults/adults ?_20 14-19 9-13 ?_8;

{18 Mesial_teeth,_average_number_of_mid-crown_denticles_per_five_millimetres_on_distal_carina_(DC)_in_subadults/adults ?_20 14-19 9-13 ?_8;

{19 Mesial_teeth,_denticle_size_(except_in_embryos_and_hatchlings) minute_denticles,_more_than_250_denticles_on_the_distal_carina normal_in_height,_between_15_to_250_denticles_on_the_distal_carina very_larges_denticles,_less_than_15_denticles_on_the_distal_carina;

{20 Mesial_teeth,_denticles_on_mesial_carina rounded_and_symmetrically_convex_ rounded_and_asymmetrically_convex strongly_hooked/pointed,_denticles_with_a_tip_pointing_apically;

{21 Mesial_teeth,_denticles_on_distal_carina rounded_and_symmetrically_convex_ rounded_and_asymmetrically_convex strongly_hooked/pointed,_denticles_with_a_tip_pointing_apically;

{22 Mesial_teeth,_size_of_mesial_denticles_relative_to_distal_denticles_(DSDI) mesial_and_distal_denticles_of_same_size,_0.8_<_DSDI_<1.2 mesial_denticles_larger_than_distal_ones,_DSDI_<_0.8 distal_denticles_larger_than_mesial_ones,_DSDI_>_1.2;

{23 Mesial_teeth,_denticles_contiguous_over_tip_or_very_close_to_the_apex present absent_;

{24 Mesial_teeth,_interdenticular_sulci absent present,_short present,_long_and_well-developed;

{25 Mesial_teeth,_flutes_(i.e.,_subparallel_longitudinal_grooves_separated_by_acute_ridges)_on_the_crown absent present_on_the_lingual_surface_only present_on_both_labial_and_lingual_surfaces present_on_the_labial_surface_only;

{26 Mesial_teeth,_longitudinal_groove_on_the_labial_and/or_lingual_side_of_the_crown absent present,_a_single_groove_centrally_positioned present,_a_single_groove_mesially_positioned;

{27 Mesial_teeth,_longitudinal_ridge,_different_of_flutes,_on_the_lingual_side_of_the_crown absent present,_a_single_ridge_centrally_positioned present,_more_than_one_ridge;

{28 Mesial_teeth,_basal_striations,_different_of_flutes,_on_both_lingual_and_labial_sides_of_the_crown absent present;

{29 Lateral_teeth,_constriction_between_root_and_crown absent constriction_weak,_base_of_crown_base_occupying_more_than_85%_of_largest_crown_width_mesiodistally constriction_important,_base_of_crown_base_occupying_85%_or_less_of_largest_crown_width_mesiodistally;

{30 Lateral_teeth,_constriction_between_root_and_crown_along_the_tooth_row present_in_some_teeth present_in_all_teeth;

{31 Lateral_teeth,_height_of_the_largest_crown_(CH_in_centimetres)_in_subadults/adults CH_?_1 1_<_CH_?_6 CH_>_6;

{32 Lateral_teeth,_labiolingual_compression_of_the_crown_(CBR_=_CBW/CBL) important,_CBR_?_0.5,_tooth_strongly_flattened normal,_0.5_<_CBR_?_0.75 weak,_CBR_>_0.75,_tooth_incrassate_or_subcircular;

{33 Lateral_teeth,_baso-apical_elongation_of_the_crown_(CHR_=_CH/CBL) weak,_CHR_?_1.5 normal,_1.5_<_CHR_?_2.5 important,_CHR_>_2.5;

{34 Lateral_teeth,_distal_margin_of_crown_in_lateral_view strongly_concave slightly_concave,_roughly_straight,_or_straight,_apex_positioned_at_the_same_level_as_distal_profile convex,_apex_positioned_mesial_to_mesial_profile sigmoid,_basal_half_concave_and_apical_half_convex sigmoid,_basal_half_convex_and_apical_half_concave;

{35 Lateral_teeth,_mesial_margin_of_crown_in_lateral_view strongly_convex slightly_convex,_almost_straight;

{36 Lateral_teeth,_mesiodistal_curvature_of_the_labial_surface_of_the_crown_at_one_third_of_the_crown convex surface_centrally_positioned_on_the_crown_roughly_flattened surface_centrally_positioned_on_the_crown_concave,_labial_depression_restricted_to_the_crown_base surface_centrally_positioned_on_the_crown_concave,_labial_depression_extends_along_the_basal_half_of_the_crown_or_more_apically;

{37 Lateral_teeth,_concave_surface_adjacent_to_carinae_all_along_the_crown absent present_on_labial_surface_and_adjacent_to_distal_carina present_on_lingual_surface_and_adjacent_to_distal_carina present_on_labial_surface_and_adjacent_to_both_mesial_and_distal_carinae present_on_lingual_surface_and_adjacent_to_both_mesial_and_distal_carinae;

{38 Lateral_teeth,_outline_of_basal_cross-section_of_the_crown subcircular lenticular_or_lanceolate elliptical_or_bean-shaped_(i.e.,_longitudinal_depression_centrally_positioned_on_one_side_only) 8-shaped_(i.e.,_longitudinal_depression_centrally_positioned_on_both_lingual_and_labial_margins) Subrectangular;

{39 Lateral_teeth,_mesial_carina present absent;

{40 Lateral_teeth,_mesial_carina denticulated non-denticulated;

{41 Lateral_teeth,_distal_carina present absent;

{42 Lateral_teeth,_distal_carina denticulated non-denticulated;

{43 Lateral_teeth,_extension_of_mesial_carina_relative_to_distal_carina mesial_carina_extends_at_the_same_level_or_terminates_more_apically_than_the_distal_carina mesial_carina_extends_more_basally_than_the_distal_carina;

{44 Lateral_teeth,_mesial_carina,_and_if_denticulated,_basalmost_serration_of_the_mesial_carina terminates_around_mid-height_of_crown_or_more_apically extends_to_base_of_crown_or_slightly_above_the_cervix terminates_well_beneath_the_cervix;

{45 Lateral_teeth,_twisted_mesial_carina_in_some_crowns absent,_mesial_carina_centrally_positioned_on_mesial_margin_or_weakly_curved_lingually_towards_the_base_in_all_teeth present,_mesial_carina_strongly_twisting_onto_the_mesiolingual_surface_in_some_teeth;

{46 Lateral_teeth,_split_carina_in_some_teeth: absent present_in_the_mesial_carina present_in_the_distal_carina;

{47 Lateral_teeth,_distal_carina,_and_if_denticulated,_basalmost_serration_of_the_distal_carina extends_to_the_cervix_or_just_above_it terminates_well_beneath_the_cervix terminates_well_above_the_cervix;

{48 Lateral_teeth,_profile_of_the_distal_carina_on_the_crown_in_distal_view straight_or_very_slightly_bowed strongly_bowed_or_sigmoid;

{49 Lateral_teeth,_position_of_distal_carina_on_the_crown_in_distal_view centrally_positioned_or_slightly_displaced,_crown_subsymmetrical strongly_labially_deflected,_crown_asymmetrical;

{50 Lateral_teeth,_average_number_of_denticles_per_five_millimeters_on_mesial_carina_at_two-thirds_height_of_the_crown_(MCA)_in_subadults/adults: ?_30 16-29 9-15 ?_8;

{51 Lateral_teeth,_average_number_of_mid-crown_denticles_per_five_millimetres_on_distal_carina_(DC)_in_subadults/adults ?_30 16-29 9-15 ?_8;

{52 Lateral_teeth,_denticle_number_on_both_mesial_and_distal_carinae_(except_in_embryos_and_hatchlings) more_than_250_denticles_(minute_denticles_or_very_large_number_of_denticles_of_normal_size) between_15_to_250_denticles_(denticles_of_average_size)_ less_than_15_denticles_(very_large_denticles_or_very_small_number_of_small_denticles);

{53 Lateral_teeth,_shape_of_denticles_on_mesial_carina_in_lateral_view symmetrically_convex_ asymmetrically_convex hooked/pointed;

{54 Lateral_teeth,_shape_of_denticles_on_distal_carina_in_lateral_view symmetrically_convex_ asymmetrically_convex hooked/pointed;

{55 Lateral_teeth,_shape_of_mesial_margin_of_rounded_denticles_on_mesial_carina_in_lateral_view parabolic subrectangular,_with_flattened_surface;

{56 Lateral_teeth,_shape_of_distal_margin_of_rounded_denticles_on_distal_carina_in_lateral_view parabolic subrectangular,_with_flattened_surface semi-circular;

{57 Lateral_teeth,_shape_of_denticles_at_two-thirds_height_of_the_crown_(MC-MA)_on_mesial_carina_in_lateral_view longer_apicobasally_than_mesiodistally,_vertical_subrectangular_ as_long_mediodistally_as_apicobasally,_subquadrangular longer_mediodistally_than_apicobasally,_horizontal_subrectangular;

{58 Lateral_teeth,_shape_of_mid-crown_denticles_(DC)_on_distal_carina_in_lateral_view as_long_mediodistally_as_apicobasally,_subquadrangular longer_mediodistally_than_apicobasally,_horizontal_subrectangular longer_apicobasally_than_mesiodistally,_vertical_subrectangular_;

{59 Lateral_teeth,_denticle_size_along_the_carinae regular,_gradual_change_in_denticle_size irregular,_sporadic_change_in_denticle_size;

{60 Lateral_teeth,_biconvex_apical_denticles_(i.e.,_biconvex_external_margin_of_denticle)_on_mesial_carina_in_lateral_view absent present;

{61 Lateral_teeth,_orientation_of_mesiodistal_axis_of_apical_denticles_on_mesial_carina_in_lateral_view perpendicular_to_mesial_margin inclined_apically_from_mesial_margin;

{62 Lateral_teeth,_orientation_of_mesiodistal_axis_of_mid-crown_denticles_on_distal_carina_in_lateral_view perpendicular_to_distal_margin inclined_apically_from_distal_margin;

{63 Lateral_teeth,_average_number_of_denticles_on_mesial_carina higher_number_of_denticles_basally_than_at_the_mid-crown lower_number_of_denticles_basally_than_at_the_mid-crown subequal_number_of_denticles_basally_than_at_the_mid-crown;

{64 Lateral_teeth,_average_number_of_denticles_on_mesial_carina higher_number_of_denticles_apically_than_at_the_mid-crown lower_number_of_denticles_apically_than_at_the_mid-crown subequal_number_of_denticles_apically_than_at_the_mid-crown;

{65 Lateral_teeth,_average_number_of_denticles_on_distal_carina_(except_in_embryos_and_hatchlings) higher_number_of_denticles_basally_than_at_the_mid-crown subequal_or_lower_number_of_denticles_basally_than_at_the_mid-crown;

{66 Lateral_teeth,_average_number_of_denticles_on_distal_carina higher_number_of_denticles_apically_than_at_the_mid-crown lower_number_of_denticles_apically_than_at_the_mid-crown subequal_number_of_denticles_apically_than_at_the_mid-crown;

{67 Lateral_teeth,_size_of_mesial_denticles_relative_to_distal_denticles_(DSDI) mesial_and_distal_denticles_of_same_size,_0.8_<_DSDI_<1.2 mesial_denticles_larger_than_distal_ones,_DSDI_<_0.8 distal_denticles_larger_than_mesial_ones,_DSDI_>_1.2;

{68 Lateral_teeth,_distal_denticles_on_the_apex contiguous_over_tip,_or_very_close_to_the_apex distal_denticles_disappear_well_beneath_apex;

{69 Lateral_teeth,_interdenticular_space_between_mid-crown_denticles_on_the_distal_carina narrow,_less_than_one_third_of_the_denticle_width broad,_more_than_one_third_of_the_denticle_width;

{70 Lateral_teeth,_interdenticular_sulci_between_apical_denticles_on_the_mesial_carina absent present,_short_and_poorly_developed,_shorter_than_proximodistal_denticle_height_ present,_long_and_well-developed,_equal_or_longer_than_proximodistal_denticle_;

{71 Lateral_teeth,_interdenticular_sulci_between_mid-crown_denticles_on_the_distal_carina absent present,_short_and_poorly_developed,_shorter_than_proximodistal_denticle_height_ present,_long_and_well-developed,_equal_or_longer_than_proximodistal_denticle_;

{72 Lateral_teeth,_interdenticular_sulci_between_basalmost_denticles_on_the_distal_carina absent present,_short_and_poorly_developed,_shorter_than_proximodistal_denticle_height_ present,_long_and_well-developed,_equal_or_longer_than_proximodistal_denticle_;

{73 Lateral_teeth,_flutes_(i.e.,_subparallel_longitudinal_grooves_separated_by_acute_ridges)_on_the_crown absent present_on_the_lingual_surface present_on_labial_surface_or_both_labial_and_lingual_surfaces;

{74 Lateral_teeth,_average_number_of_flutes_on_the_crown 1-7 7-8 >8;

{75 Lateral_teeth,_large_transverse_undulations_on_the_crown_in_some_teeth absent present,_tenuous_and_barely_visible_with_light present,_pronounced_and_well_visible_with_light;

{76 Lateral_teeth,_large_transverse_undulations_on_the_crown_in_some_teeth_when_present just_a_few numerous_and_closely_packed;

{77 Lateral_teeth,_marginal_undulations_(i.e.,_short_undulations_adjacent_to_carinae)_in_some_teeth absent_ present_and_short,_the_mesiodistal_elongation_is_less_than_four_times_the_space_separating_each_undulation present_and_elongated,_the_mesiodistal_elongation_is_longer_than_four_times_the_space_separating_each_undulation;

{78 Lateral_teeth,_marginal_undulations_in_some_teeth present_and_shallow,_only_visible_with_light present_and_pronounced,_well_visible_in_lateral_view;

{79 Lateral_teeth,_marginal_undulations_in_some_teeth present_only_on_the_mesial_side_of_the_crown present_only_on_the_distal_side_of_the_crown present_on_both_mesial_and_distal_sides;

{80 Lateral_teeth,_marginal_undulations_in_some_teeth present_and_mesio-distally_oriented present_and_diagonally_oriented;

{81 Lateral_teeth,_longitudinal_groove_on_the_labial_and/or_lingual_surface_of_the_crown absent present,_a_single_groove_centrally_positioned present,_a_single_groove_adjacent_to_mesial_carina present,_two_grooves_or_more;

{82 Lateral_teeth,_elongated_longitudinal_and_rounded_ridge_(differing_from_flutes)_on_the_lingual_surface_of_the_crown absent present,_a_single_ridge_centrally_positioned present,_two_or_three_ridges present,_several_fainted_ridges;

{83 Enamel_surface_texture smooth_or_irregular_(non-oriented)_texture_ braided_(oriented)_texture_not_clearly_visible_with_light braided_(oriented)_texture_clearly_visible_with_or_without_light deeply_veined/anastomosed_(oriented)_texture;

{84 Coarse_enamel_surface_texture remains_baso-apically/diagonally_oriented_or_slightly_curved_basally_close_to_the_carinae strongly_curved_basally_close_to_the_carinae;

{85 Root,_shape_in_lateral_view with_subparallel_mesial_and_distal_margins with_convex_margins,_root_significantly_larger_than_base_crown;

{86 Root,_distal_shape_in_lateral_view broad strongly_tapered_apically;

{87 Root,_outline_of_mid-root_in_cross_section oval_to_subcircular 8-shape_(i.e.,_longitudinal_depression_centrally_positioned_on_both_lingual_and_labial_margins) bean-shaped_(i.e.,_longitudinal_depression_centrally_positioned_on_one_side_only);

{88 Root,_form_of_the_resorption_pit_in_lingual_view deep_and_well-delimited_depression shallow_concavity_or_absent;

{89 Root,_transversal_undulations_below_the_cervix_in_some_crowns: absent present;

{90 Root,_apicobasal_height_in_lateral_view less_than_twice_the_apicobasal_height_of_the_crown twice_or_more_the_apicobasal_height_of_the_crown;

;

ccode + 0 2 12 19 29 31 52 *;

proc /;

comments 0

;
